# Triblock Glycopolymers with Two 10-mer Blocks of Activating Sugars Enhance the Activation of Acrosomal Exocytosis in Mouse Sperm

**DOI:** 10.1021/acsbiomedchemau.4c00012

**Published:** 2024-04-29

**Authors:** Luz C. Mendez, Mitchell Kennedy, Surita R. Bhatia, Nicole S. Sampson

**Affiliations:** † Department of Chemistry, Stony Brook University, Stony Brook, New York 11794-3400, United States; ‡ Department of Chemistry, University of Rochester, Rochester, New York 14627-0216, United States

**Keywords:** ring-opening, metathesis, polymerization, small-angle X-ray scattering, polyvalent, norbornene

## Abstract

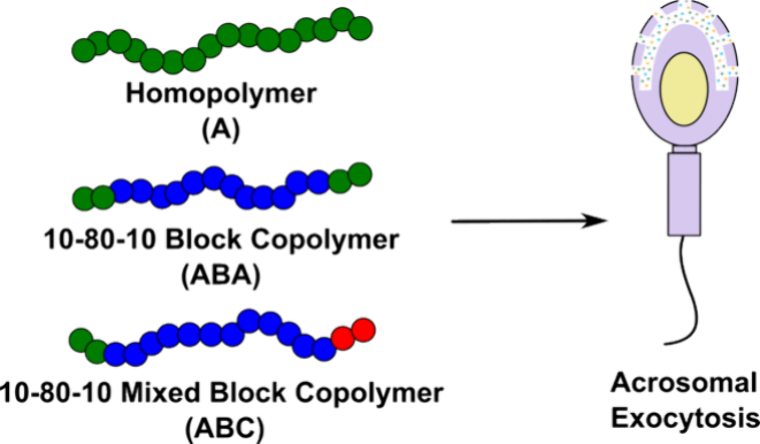

Carbohydrate recognition is imperative for the induction of sperm acrosomal exocytosis (AE), an essential phenomenon in mammalian fertilization. In mouse sperm, polynorbornene 100-mers displaying fucose or mannose moieties were effective at inducing AE. In contrast, glycopolymers exhibiting glucose sugars resulted in no AE activation. To further elucidate the role of ligand density on the activation of AE in mouse sperm, a triple-stain flow cytometry assay was employed to determine the efficacy of polynorbornene block copolymers with barbell-like sequences as initiators of AE. Triblock (ABA or ABC) copolymers were synthesized by ring-opening metathesis polymerization (ROMP) with one or two activating sugars, mannose or fucose, and one nonactivating sugar, glucose. The active ligand fractions in the polymers varied from 10, 20, or 40%. Simultaneously, random copolymers comprising 20% activating ligands were prepared to confirm the importance of ligand positionality in AE activation in mouse sperm. Polynorbornene 100-mers possessing two 10-mer blocks of activating sugars were the most effective copolymers at inducing AE with levels of AE comparable to their homopolymer counterparts and more effective than their random analogues. Small-angle X-ray scattering (SAXS) was then performed to verify that there were no differences in the conformations of the glycopolymers contributing to their varying AE activity. SAXS data analysis confirmed that all of the glycopolymers assumed semiflexible cylindrical structures with similar radii and Kuhn lengths. These findings suggest that the overall ligand density of the sugar moieties in the polymer is less important than the positionality of short blocks of high-density ligands for AE activation in mouse sperm.

Protein–carbohydrate interactions are the crux of two key processes required for successful mammalian fertilization: adherence of spermatozoa to the oviduct and binding of spermatozoa to the egg cell’s zona pellucida (ZP). During fertilization, sperm that enters the female reproductive tract and reaches the oviduct are stored in a reservoir.^1,2^ Lectins on the sperm head bind to oligosaccharides on the surface of the oviductal epithelium, maximizing the probability of fertilization by maintaining sperm viability and regulating the rate of sperm capacitation and motility.^3−5^ Carbohydrate recognition then plays a vital role in the induction of acrosomal exocytosis (AE) in sperm. AE is a calcium-dependent event in which enzymes stored in a cap-like granule, known as the acrosome, are released and render the sperm capable of sperm–egg fusion.^6,7^ Although the activation mechanism remains elusive, it is hypothesized that receptors on the sperm can bind to glycoproteins coating the egg cell’s ZP, contributing to the induction of AE.^8−10^

A previous study conducted by our group determined that glycopolymers designed to mimic the terminal sugar residues on the ZP could induce AE in mouse sperm.^11^ Specifically, polynorbornene glycopolymers displaying 100 mannose, fucose, or *N*-acetylglucosamine moieties were able to induce AE in a concentration-dependent manner. Furthermore, these glycopolymers were found to initiate AE via different receptors but eventually converged into the same signaling pathways as that of the mouse ZP.^11^ However, there is still no definitive information about the identities or locations of the receptors activated by glycopolymers. In addition, the scope of the previous study was limited to homopolymers and did not necessarily address the potential combinations of sugars that could contribute to the initiation of AE or the complexity of carbohydrate structures on the ZP. Thus, we sought to elucidate the effects of ligand density on AE induction in mouse sperm.

In this study, ring-opening metathesis polymerization (ROMP) of norbornene-conjugated sugars was employed to synthesize random copolymers and block copolymers comprised of two or three different sugars: mannose, fucose, and/or glucose. As demonstrated by our previous fluorescence microscopy and flow cytometry studies, mannose and fucose ligands can initiate AE in mouse sperm, while glucose moieties do not.^11,12^ The block copolymers were designed to mimic a barbell; this triblock sequence (ABA or ABC) resulted in polymers where the first and third block displayed AE activating sugars, mannose or fucose, and the second block of the polymer consisted of the nonactivating sugar, glucose. Different iterations of these barbell sequences were synthesized with varying lengths of blocks of the activating sugars: 5 blocks, 10 blocks, or 20 blocks. In addition, random copolymers composed of 20% activating sugars were prepared as a comparison to the sequence-specific triblock copolymers (Figure 1). The potencies of random and block copolymers to activate AE were compared to previously synthesized homopolymers.^13^

**Figure 1:**
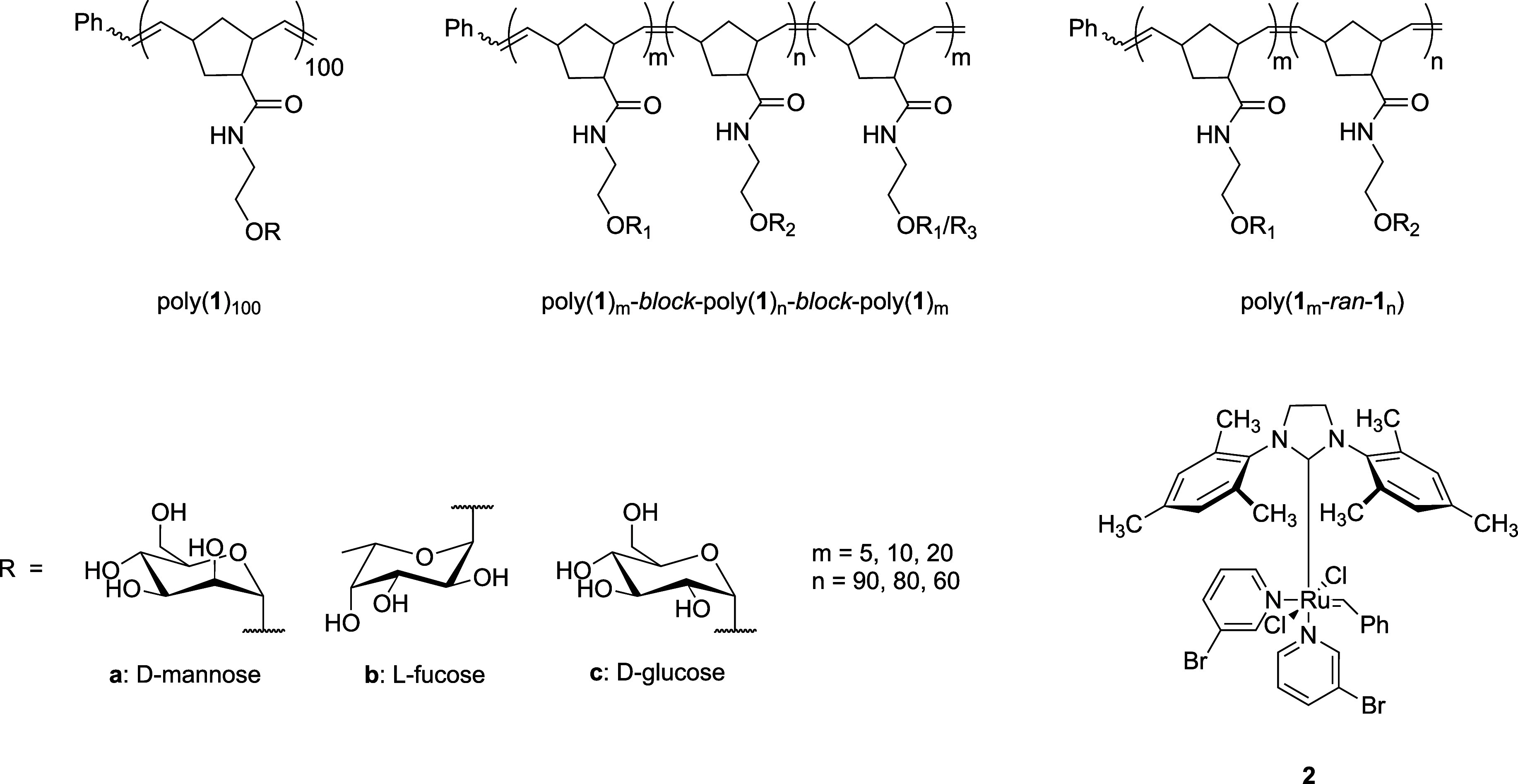
Backbone structures of norbornene homopolymers, triblocks, or random copolymers displaying fucose, mannose, and/or glucose ligands. Grubbs’ third-generation catalyst, **2**, was used in the polymerization of norbornene sugar monomers.

Small-angle X-ray scattering (SAXS) was then utilized to quantify the conformations, rigidities, and lengths of the polymers to determine whether the arrangement of sugar ligands resulted in differences among the structures. By comparing the random copolymers and block copolymers to previously synthesized homopolymers consisting of fucose, mannose, and glucose, we were able to ascertain, which arrangements of ligands were the most effective at inducing AE in mouse sperm.

## Materials and Methods

### Materials

All experiments performed on mice were approved by Stony Brook University IACUC (Protocol 252156) and were conducted in accordance with the National Institute of Health and the United States Department of Agriculture guidelines. Anhydrous dimethyl sulfoxide (DMSO), soybean trypsin inhibitor (SBTI), propidium iodide (PI), and bovine serum albumin (BSA), fraction V, were purchased from Sigma-Aldrich (St. Louis, MO). Alexa Fluor 488 tetrafluorophenyl (TFP) ester, SYTO 17, and Dulbecco’s phosphate-buffered saline (DPBS) were purchased from Life Technologies (Carlsbad, CA). All other chemicals and supplies were purchased from Sigma-Aldrich, Fisher Scientific (Hampton, NH) or VWR (Radnor, PA).

### Fluorescent Stain Preparation and Glycopolymer Solution Storage

PI was purchased as a stock solution dissolved in water (2.4 mM) and stored at 4 °C. A 5 mM SYTO 17 solution was diluted in anhydrous DMSO (1 mM) and stored at −20 °C as aliquots. Alexa Fluor 488 soybean trypsin inhibitor conjugates (SBTI-Alexa 488) were prepared by previously reported methods^13^ and stored at −20 °C as aliquots. Stock solutions of all polymers were prepared by dissolving deacetylated, purified polymers in distilled deionized (ddI) water. The stock solutions were then aliquoted and stored at −20 °C at a polymer concentration of 100 μM.

### Sperm Treatment, Flow Cytometry Assays, and Data Analysis

Preparation of cell media, treatment of mouse sperm, and all flow cytometry experiments were conducted following previously published methods.^12,13^ 50,000 events were recorded for each sample. When treated with the negative control, DPBS, the average AE% of mouse sperm was 7.2%. The AE% of mouse sperm induced by each polymer at different concentrations was compared to the AE% of mouse sperm treated with DPBS. In addition, the AE% of two consecutive concentrations of polymer were compared. Significant differences in AE% were calculated using one-way analysis of variance (ANOVA) with GraphPad Prism 10. The data represent the mean ± standard error of the mean of at least three independent experiments using at least two separate batches of polymer. For any statistical significance, *p < 0.05, **p < 0.01, ***p < 0.001, ****p < 0.0001.

### General Methods

Air and moisture-sensitive reactions were performed in a glovebox under a nitrogen atmosphere. Analytical thin-layer chromatography (TLC) was performed on aluminum-backed sheets coated with silica gel 60F254. Non-UV active compounds were detected on the plates by staining with 10% (w/v) phosphomolybdic acid in ethanol. Flash column chromatography was performed using a CombiFlash system with RediSep normal phase silica columns (Teledyne ISCO, silica gel 60, 230–400 mesh). Chloroform-*d* (CDCl_3_) and deuterium oxide (D_2_O) were purchased from Cambridge Isotope Laboratories (Tewksbury, MA) and used for the collection of nuclear magnetic resonance (NMR) spectra. NMR spectra were recorded on a Bruker Ascend 700 spectrometer (^1^H-700 MHz, ^13^C-176 MHz) and a Bruker 400 Nanobay spectrometer (^1^H-400 MHz, ^13^C-100 MHz). Chemical shifts reported are given in parts per million relative to the residual solvent peaks.

### Gel Permeation Chromatography (GPC)

Analysis was performed on a system comprising a Shimadzu SCL-10A controller, a Shimadzu LC-20AT pump, and a Shimadzu CTO-10AS column oven equipped with two combined Phenogel columns: 5 μm 50 Å (300 × 4.6 mm, 100–3k) and 5 μm 10E3 Å (300 × 4.6 mm, 1k–75k). This was coupled to a Brookhaven Instruments BI-DNDC refractometer. The mobile phase used was HPLC-grade tetrahydrofuran (THF) filtered through a 0.2 μm nylon membrane. The protected polymers were dissolved in the same mobile phase and filtered through a 0.45 μm polytetrafluoroethylene (PTFE) membrane before 100 μL of the sample was injected into the system. Analysis was then performed at 30 °C and with a flow rate of 0.35 mL min^–1^. Polystyrene was used as a standard for the calibration.

### Preparation of Homopolymers

Grubbs’ third-generation catalyst, **2**, was prepared as described in the literature.^14^d-Mannose (**1a′**), l-fucose (**1b**′****), and d-glucose (**1c**′****) norbornene monomers, poly(**1a**)_100_, poly(**1b**)_100_, poly(**1c**)_100_, and acetylated precursors were prepared as previously described.^11,15^ Poly(**1a**)_100_ and poly(**1b**)_100_ were the same polymers from a previously reported study.^13^

### General Preparation of Acetylated Random Copolymers

To a septum-sealed vial containing nitrogen and a stir bar was added a solution of **2** (1.7 mM, 1 equiv) in dichloromethane (146 μL) and chilled to 0 °C for 10 min. A solution of **1a**′**** or **1b**′**** (34.6 mM, 20 equiv) in dichloromethane (300 μL) was then combined with a solution of **1c**′**** (138.1 mM, 80 equiv) in dichloromethane (300 μL). The mixture was then added to the vial containing **2**, and the reaction was allowed to initiate at 0 °C for 10 min. The reaction mixture was then stirred at 25 °C for an additional 90 min, and TLC was used to monitor the disappearance of monomers. An excess of ethyl vinyl ether (0.1 mL) was used to terminate the reaction, and the solution was allowed to stir for 30 min at 25 °C. Ethyl ether chilled to −20 °C was used to precipitate the polymer, resulting in an off-white solid. After drying in vacuo to remove residual solvents, the polymers were analyzed by GPC and NMR to determine their dispersities and purity.

Poly(**1a**′****_20_-*ran*-**1c**′****_80_): (Yields: 64–87%) ^1^H NMR (700 MHz, CDCl_3_) δ 7.37–7.33 (m), 5.92 (br, s), 5.51–5.13 (m), 5.10 (br, s), 4.99 (br, s), 4.84 (br, s), 4.56 (br, s), 4.29 (br, s), 4.15 (br, s), 3.98 (br, s), 3.91–3.65 (m), 3.54 (br, s), 3.45–3.21 (m), 3.04 (br, s), 2.71 (br, s), 2.28 (br, s), 2.18 (s), 2.15–1.99 (m), 1.98–1.90 (m), 1.74 (s), 1.15 (br, s). ^13^C NMR (176 MHz, CDCl_3_) δ 174.85, 170.56, 170.10, 170.01, 169.62, 169.40, 133.54, 133.13, 131.63, 100.78, 97.39, 72.65, 71.77, 71.26, 69.24, 69.09, 68.55, 68.21, 67.29, 65.98, 62.35, 61.79, 52.16, 52.10, 48.12, 43.31, 42.03, 39.10, 38.86, 36.95, 20.83, 20.73, 20.67, 20.54.

Poly(**1b**′****_20_-*ran*-**1c**′****_80_): (Yields: 53–56%) ^1^H NMR (700 MHz, CDCl_3_) δ 7.37–7.33 (m), 5.91 (br, s), 5.50–5.02 (m), 4.97 (br, s), 4.55 (br, s), 4.29 (br, s), 4.15 (br, s), 3.83 (br, s), 3.80–3.64 (m), 3.54 (br, s), 3.43–3.23 (m), 3.03 (br, s), 2.70 (br, s), 2.27 (br, s), 2.18 (s), 2.12–1.99 (m), 1.98–1.89 (m), 1.78 (br, s), 1.15 (br, s). ^13^C NMR (176 MHz, CDCl_3_) δ: 174.73, 174.38, 170.55, 170.09, 169.39, 133.66, 132.86, 131.55, 100.78, 96.23, 72.63, 71.75, 71.24, 70.96, 69.26, 68.19, 68.04, 67.87, 67.58, 64.51, 61.77, 52.22, 51.89, 47.90, 43.33, 41.97, 39.08, 36.83, 20.82, 20.72, 20.67, 20.59, 20.53, 15.85.

### Monomer Incorporation Rates into Norbornene Random Copolymers

For the preparation of the 20:80 random copolymers, a solution of **2** (2.1 mM, 1 equiv) in dichloromethane (122 μL) was added to a septum-sealed vial containing nitrogen and a stir bar. The vial and its contents were chilled to 0 °C for 10 min. A solution of **1a**′**** or **1b**′**** (42.1 mM, 20 equiv) in dichloromethane (300 μL) was then combined with a solution of **1c**′**** (169 mM, 80 equiv) in dichloromethane (300 μL). The mixture was then added to the vial containing **2**, and the reaction was run at 0 °C for 90 s. An excess of ethyl vinyl ether (0.1 mL) was used to terminate the reaction and allowed to stir for 30 min at 25 °C. Ethyl ether chilled to −20 °C was used to precipitate the polymer, resulting in an off-white solid. After being dried in vacuo to remove residual solvents, the polymers were analyzed by NMR to determine the proton integration of the monomers. To synthesize the 50:50 random copolymers, the ratios of [**2**]/[**1a**′****/**1b**′****]/[**1c**′****] was 1:50:50.

### General Preparation of Acetylated Block Copolymers

For preparation of the 10:80:10 block copolymers, a solution of **2** (1.6 mM, 1 equiv) in dichloromethane (230 μL) was added to a septum-sealed vial containing nitrogen and a stir bar. The vial and its contents were chilled to 0 °C for 10 min. A solution of **1a**′**** or **1b**′**** (16.3 mM, 10 equiv) in dichloromethane (300 μL) was then added to the vial, and the reaction was allowed to initiate at 0 °C for 10 min. The reaction mixture was stirred at 25 °C for an additional 25 min and monitored by TLC to ensure that all of the monomer had reacted. A solution of **1c’** (130.1 mM, 80 equiv) in dichloromethane (400 μL) was then added to the reaction vial and allowed to stir for 90 min at 25 °C. The reaction was monitored by TLC once again. Finally, a solution of **1a**′**** or **1b**′**** (16.3 mM, 10 equiv) in dichloromethane (300 μL) was added to the vial, stirred for 35 min at 25 °C, and monitored by TLC. The reaction was then terminated with an excess of ethyl vinyl ether (0.1 mL) and stirred for 30 min at 25 °C. Ethyl ether chilled to −20 °C was used to precipitate the polymer, resulting in a light brown solid. After drying in vacuo to remove residual solvents, the polymers were analyzed by GPC and NMR to determine dispersities and purity. To synthesize the 5:90:5 and 20:60:20 block copolymers, the ratios of [**2**]/[**1a**′****/**1b**′****]/[**1c**′****]/[**1a**′****/**1b**′****] were 1:5:90:5 and 1:20:60:20, respectively.

Poly(**1a**′****)_5_-*block*-poly(**1c**′****)_90_-*block*-poly(**1a**′****)_5_: (Yields: 64–83%) ^1^H NMR (700 MHz, CDCl_3_) δ 7.37–7.33 (m), 5.94 (br, s), 5.50–5.13 (m), 5.09 (br, s), 4.98 (br, s), 4.83 (br, s), 4.55 (br, s), 4.29 (br, s), 4.15 (br, s), 3.98 (br, s), 3.91–3.65 (m), 3.53 (br, s), 3.45–3.23 (m), 3.03 (br, s), 2.70 (br, s), 2.27 (br, s), 2.17 (s), 2.15–2.00 (m), 1.99–1.90 (m), 1.80 (s), 1.15 (br, s). ^13^C NMR (176 MHz, CDCl_3_) δ: 174.83, 174.43, 170.68, 170.22, 170.16, 169.75, 169.52, 133.75, 133.00, 131.79, 100.94, 97.61, 72.78, 71.90, 71.39, 69.37, 69.19, 68.70, 68.34, 67.48, 66.16, 62.47, 61.90, 52.39, 51.99, 48.15, 43.56, 42.09, 39.21, 39.02, 36.86, 20.95, 20.87, 20.80, 20.67.

Poly(**1a**′****)_10_-*block*-poly(**1c**′****)_80_-*block*-poly(**1a**′****)_10_: (Yields: 73–80%) ^1^H NMR (700 MHz, CDCl_3_) δ 7.37–7.33 (m), 5.92 (br, s), 5.50–5.13 (m), 5.09 (br, s), 4.98 (br, s), 4.83 (br, s), 4.55 (br, s), 4.29 (br, s), 4.15 (br, s), 3.98 (br, s), 3.90–3.65 (m), 3.53 (br, s), 3.43–3.20 (m), 3.03 (br, s), 2.70 (br, s), 2.27 (br, s), 2.17 (s), 2.14–2.00 (m), 1.98–1.89 (m), 1.84 (br, s), 1.16 (br, s). ^13^C NMR (176 MHz, CDCl_3_) δ: 174.78, 174.25, 170.66, 170.21, 170.11, 169.73, 169.51, 133.72, 133.13, 131.73, 100.90, 97.81, 97.62, 72.77, 71.90, 71.38, 69.37, 69.20, 68.70, 68.34, 67.46, 66.08, 62.48, 61.91, 52.32, 52.06, 48.05, 43.63, 42.23, 39.21, 39.01, 36.74, 20.95, 20.87, 20.81, 20.68.

Poly(**1a**′****)_10_-*block*-poly(**1c**′****)_80_-*block*-poly(**1b**′****)_10_: (Yields: 84–87%) ^1^H NMR (700 MHz, CDCl_3_) δ 7.37–7.33 (m), 5.97 (br, s), 5.49–5.02 (m), 4.97 (br, s), 4.82 (br, s), 4.55 (br, s), 4.28 (br, s), 4.14 (br, s), 3.96 (br, s), 3.83 (br, s), 3.80–3.63 (m), 3.52 (br, s), 3.43–3.21 (m), 3.03 (br, s), 2.70 (br, s), 2.26 (br, s), 2.21–2.15 (m), 2.14–1.99 (m), 1.98–1.90 (m), 1.85 (br, s), 1.14 (br, s). ^13^C NMR (176 MHz, CDCl_3_) δ: 174.67, 174.41, 170.64, 170.17, 170.10, 169.73, 169.47, 133.65, 132.97, 131.57, 100.89, 97.59, 96.41, 72.72, 71.84, 71.34, 71.05, 69.32, 69.12, 68.61, 68.28, 68.15, 67.96, 67.45, 65.99, 64.59, 62.45, 61.85, 52.36, 52.06, 48.20, 43.54, 42.06, 39.17, 39.00, 37.01, 20.89, 20.81, 20.76, 20.67, 20.62, 15.93.

Poly(**1a**′****)_20_-*block*-poly(**1c**′****)_60_-*block*-poly(**1a**′****)_20_: (Yields: 91–99%) ^1^H NMR (700 MHz, CDCl_3_) δ 7.37–7.33 (m), 5.92 (br, s), 5.50–5.14 (m), 5.09 (br, s), 4.98 (br, s), 4.83 (br, s), 4.55 (br, s), 4.29 (br, s), 4.15 (br, s), 3.98 (br, s), 3.90–3.64 (m), 3.53 (br, s), 3.44–3.21 (m), 3.04 (br, s), 2.70 (br, s), 2.42–2.23 (m), 2.17 (s), 2.15–1.99 (m), 1.98–1.90 (m), 1.77 (br, s), 1.17 (br, s). ^13^C NMR (176 MHz, CDCl_3_) δ: 174.75, 174.30, 170.61, 170.16, 170.06, 169.68, 169.46, 133.63, 133.16, 131.70, 100.90, 97.76, 97.54, 72.73, 71.85, 71.33, 69.31, 69.10, 68.65, 68.28, 67.35, 66.03, 62.41, 61.85, 52.34, 51.89, 48.16, 43.53, 42.15, 39.14, 38.94, 36.71, 20.90, 20.81, 20.75, 20.62.

Poly(**1b**′****)_5_-*block*-poly(**1c**′****)_90_-*block*-poly(**1b**′****)_5_: (Yields: 69–78%) ^1^H NMR (700 MHz, CDCl_3_) δ 7.37–7.33 (m), 5.94 (br, s), 5.48–5.02 (m), 4.98 (br, s), 4.55 (br, s), 4.29 (br, s), 4.15 (br, s), 3.83 (br, s), 3.79–3.64 (m), 3.53 (br, s), 3.43–3.22 (m), 3.03 (br, s), 2.70 (br, s), 2.27 (br, s), 2.18 (s), 2.16–1.99 (m), 1.98–1.92 (m), 1.88 (br, s), 1.15 (br, s). ^13^C NMR (176 MHz, CDCl_3_) δ: 174.85, 174.38, 170.62, 170.16, 169.46, 133.67, 132.81, 131.75, 100.89, 96.42, 72.71, 71.84, 71.33, 71.01, 69.32, 68.28, 68.13, 67.94, 67.50, 64.61, 61.85, 52.42, 52.02, 48.02, 43.48, 42.10, 39.16, 37.05, 20.81, 20.75, 20.67, 20.62, 15.93.

Poly(**1b**′****)_10_-*block*-poly(**1c**′****)_80_-*block*-poly(**1b**′****)_10_: (Yields: 77–79%) ^1^H NMR (700 MHz, CDCl_3_) δ 7.37–7.33 (m), 5.91 (br, s), 5.49–5.01 (m), 4.97 (br, s), 4.55 (br, s), 4.28 (br, s), 4.14 (br, s), 3.83 (br, s), 3.79–3.63 (m), 3.53 (br, s), 3.43–3.22 (m), 3.03 (br, s), 2.70 (br, s), 2.27 (br, s), 2.17 (s), 2.16–1.98 (m), 1.97–1.87 (m), 1.81 (s), 1.14 (br, s). ^13^C NMR (176 MHz, CDCl_3_) δ 174.91, 174.36, 170.73, 170.28, 169.57, 133.96, 132.93, 131.81, 100.99, 96.57, 72.83, 71.96, 71.43, 71.17, 69.45, 68.39, 68.28, 68.05, 67.65, 64.70, 61.97, 52.48, 52.10, 48.13, 43.57, 42.22, 39.25, 36.83, 21.02, 20.92, 20.87, 20.79, 20.73, 16.04.

Poly(**1b**′****)_10_-*block*-poly(**1c**′****)_80_-*block*-poly(**1a**′****)_10_: (Yields: 84–89%) ^1^H NMR (700 MHz, CDCl_3_) δ 7.37–7.33 (m), 5.98 (br, s), 5.50–5.02 (m), 4.98 (br, s), 4.83 (br, s), 4.55 (br, s), 4.29 (br, s), 4.15 (br, s), 3.97 (br, s), 3.84 (br, s), 3.80–3.64 (m), 3.53 (br, s), 3.44–3.22 (m), 3.04 (br, s), 2.70 (br, s), 2.27 (br, s), 2.21–2.13 (m), 2.12–1.99 (m), 1.98–1.90 (m), 1.86 (br, s), 1.15 (br, s). ^13^C NMR (176 MHz, CDCl_3_) δ: 175.31, 174.58, 170.75, 170.29, 170.21, 169.83, 169.59, 133.84, 133.11, 131.93, 101.00, 97.68, 96.51, 72.83, 71.95, 71.44, 71.12, 69.44, 68.75, 68.39, 68.25, 68.08, 67.54, 66.12, 64.70, 62.53, 61.96, 52.48, 52.05, 48.14, 43.64, 42.25, 39.28, 39.06, 37.22, 21.01, 20.92, 20.86, 20.78, 20.73, 16.03.

Poly(**1b**′****)_20_-*block*-poly(**1c**′****)_60_-*block*-poly(**1b**′****)_20_: (Yields: 55–69%) ^1^H NMR (700 MHz, CDCl_3_) δ 7.37–7.33 (m), 5.94 (br, s), 5.49–5.01 (m), 4.98 (br, s), 4.55 (br, s), 4.29 (br, s), 4.15 (br, s), 3.83 (br, s), 3.80–3.65 (m), 3.53 (br, s), 3.44–3.21 (m), 3.04 (br, s), 2.70 (br, s), 2.27 (br, s), 2.18 (s), 2.13–1.98 (m), 1.97–1.90 (m), 1.85 (br, s), 1.15 (br, s). ^13^C NMR (176 MHz, CDCl_3_) δ 174.86, 174.42, 170.71, 170.25, 169.55, 133.82, 133.04, 131.85, 100.98, 96.47, 72.80, 71.93, 71.42, 71.13, 69.40, 68.37, 68.20, 68.04, 67.72, 64.68, 61.94, 52.40, 52.08, 48.24, 43.60, 42.25, 39.24, 37.06, 21.00, 20.90, 20.84, 20.76, 20.70, 16.02.

### General Deprotection of Random and Block Copolymers

To a reaction vial containing the acetylated polymer (50 mg) was added K_2_CO_3_ in excess. An anhydrous mixture of MeOH:THF (2:1 v/v, 4.0 mL) was added to the vial and the reaction was left to stir at 25 °C for 90 min. The reaction mixture was concentrated in vacuo, neutralized using 5.0 mL of 1 N HCl in H_2_O:THF (1:1 v/v), and then stirred for 90 min. The reaction mixture was then transferred to a prewetted cellulose ester Spectra/Por Float-A-Lyzer G2 dialysis device (MWCO 3.5–5kD, 5 mL) and dialyzed against deionized water for at least 3 d. The mixture was then lyophilized for 2 d to afford an off-white solid.

Poly(**1a**_20_-*ran*-**1c**_80_): (Yields: 17–93%) ^1^H NMR (700 MHz, D_2_O) δ 7.50–7.28 (m), 5.56–5.19 (m), 4.94–4.83 (m), 4.44 (br, s), 4.01–3.25 (m), 3.19–2.86 (m), 2.81–2.57 (m), 2.50 (br, s), 2.17–1.77 (m), 1.67 (br, s), 1.21 (br, s).

Poly(**1b**_20_-*ran*-**1c**_80_): (Yields: 86–99%) ^1^H NMR (700 MHz, D_2_O) δ 7.50–7.25 (m), 5.54–5.17 (m), 4.86 (br, s), 4.42 (br, s), 4.02–3.60 (m), 3.58–3.23 (m), 3.17–2.84 (m), 2.80–2.56 (m), 2.49 (br, s), 2.14–1.76 (m), 1.65 (br, s), 1.20 (br, s).

Poly(**1a**)_5_-*block*-poly(**1c**)_90_-*block*-poly(**1a**)_5_: (Yields: 50%) ^1^H NMR (400 MHz, D_2_O) δ 7.49–7.26 (m), 5.58–5.16 (m), 4.96–4.86 (m), 4.44 (br, s), 4.02–3.65 (m), 3.57–3.26 (m), 3.03 (br, s), 2.85–2.62 (m), 2.51 (br, s), 2.04 (br, s), 1.68 (br, s), 1.22 (br, s).

Poly(**1a**)_10_-*block*-poly(**1c**)_80_-*block*-poly(**1a**)_10_: (Yields: 82–99%) ^1^H NMR (400 MHz, D_2_O) δ 7.49–7.23 (m), 5.56–5.18 (m), 4.95–4.85 (m), 4.43 (br, s), 4.02–3.25 (m), 3.03 (br, s), 2.83–2.60 (m), 2.51 (br, s), 2.05 (br, s), 1.68 (br, s), 1.25 (br, s).

Poly(**1a**)_10_-*block*-poly(**1c**)_80_-*block*-poly(**1b**)_10_: (Yields: 84%) ^1^H NMR (700 MHz, D_2_O) δ 7.48–7.25 (m), 5.57–5.16 (m), 4.93–4.83 (m), 4.43 (br, s), 4.02–3.63 (m), 3.57–3.22 (m), 3.20–2.83 (m), 2.81–2.57 (m), 2.49 (br, s), 2.16–1.76 (m), 1.66 (br, s), 1.20 (br, s).

Poly(**1a**)_20_-*block*-poly(**1c**)_60_-*block*-poly(**1a**)_20_: (Yields: 91–99%) ^1^H NMR (400 MHz, D_2_O) δ 7.47–7.26 (m), 5.59–5.16 (m), 4.94–4.83 (m), 4.43 (br, s), 4.03–3.23 (m), 3.03 (br, s), 2.81–2.57 (m), 2.50 (br, s), 2.03 (br, s), 1.67 (br, s), 1.21 (br, s).

Poly(**1b**)_5_-*block*-poly(**1c**)_90_-*block*-poly(**1b**)_5_: (Yields: 37–91%) ^1^H NMR (400 MHz, D_2_O) δ 7.49–7.28 (m), 5.56–5.18 (m), 4.92 (br, s), 4.44 (br, s), 4.03–3.65 (m), 3.59–3.25 (m), 3.05 (br, s), 2.84–2.60 (m), 2.51 (br, s), 2.05 (br, s), 1.69 (br, s), 1.22 (br, s).

Poly(**1b**)_10_-*block*-poly(**1c**)_80_-*block*-poly(**1b**)_10_: (Yields: 5–30%) ^1^H NMR (400 MHz, D_2_O) δ 7.49–7.25 (m), 5.56–5.18 (m), 4.90 (br, s), 4.44 (br, s), 4.04–3.62 (m), 3.60–3.23 (m), 3.03 (br, s), 2.82–2.59 (m), 2.50 (br, s), 2.02 (br, s), 1.67 (br, s), 1.22 (br, s).

Poly(**1b**)_10_-*block*-poly(**1c**)_80_-*block*-poly(**1a**)_10_: (Yields: 90–94%) ^1^H NMR (700 MHz, D_2_O) δ 7.49–7.28 (m), 5.58–5.18 (m), 4.93–4.84 (m), 4.43 (br, s), 3.91–3.60 (m), 3.58–3.24 (m), 3.18–2.84 (m), 2.82–2.57 (m), 2.50 (br, s), 2.16–1.77 (m), 1.67 (br, s), 1.21 (br, s).

Poly(**1b**)_20_-*block*-poly(**1c**)_60_-*block*-poly(**1b**)_20_: (Yields: 94–97%) ^1^H NMR (400 MHz, D_2_O) δ 7.50–7.23 (m), 5.58–5.17 (m), 4.87 (br, s), 4.44 (br, s), 4.05–3.62 (m), 3.60–3.24 (m), 3.05 (br, s), 2.83–2.61 (m), 2.51 (br, s), 2.05 (br, s), 1.69 (br, s), 1.22 (br, s).

### Small-Angle X-ray Scattering (SAXS)

Experiments were conducted on the Life Science X-ray Scattering (LiX) beamline, 16-ID, at the National Synchrotron Light Source II (NSLS II) at Brookhaven National Laboratory in Upton, NY. Stock solutions of the deacetylated glycopolymers were prepared at 1% (w/v) in M16 buffer. Aliquots of the stock solutions (60 μL) were then pipetted into PCR tubes, placed in LiX holders, and measured using the automated data collection procedure at the beamline.^16^ SAXS and wide-angle X-ray scattering (WAXS) data were collected simultaneously on Pilatus 1 M (SAXS) and Pilatus 900 K (WAXS) detectors.^17^ Data collected from both detectors were then scaled and merged. Ten frames with 0.5 s exposure were averaged, and outliers were removed automatically. Buffer subtraction from the sample was normalized using the height of the water peak at 2.0 Å^–1^. Data processing and analysis were performed using the py4XS and lixtools Python scripts, as well as the SasView software package.

## Results and Discussion

### Design and Preparation of the Random and Block Copolymers

**Figure 2:**
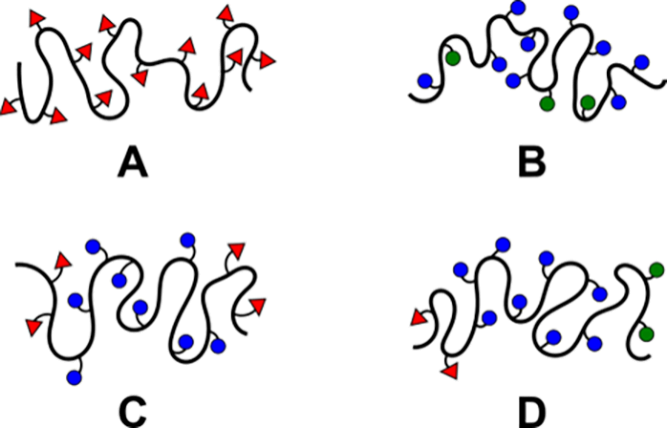
Schematic illustrations of (A) norbornene homopolymers, (B) random copolymers, (C) triblock copolymers with a single activating sugar (ABA), and (D) triblock copolymers with two activating sugars (ABC).

Our initial studies utilizing glycopolymers as mimics of physiological inducers of AE consisted solely of homopolymers with a norbornene backbone (Figure 2A).^11^ Later studies revealed that the semirigid and hydrophobic backbone of norbornene glycopolymers with 100 repeat units was necessary for efficient induction of AE in mouse sperm.^13,18^ When compared to highly flexible or hydrophilic backbones, norbornene homopolymers prepared with mannose or fucose sugars were consistently better inducers of AE. In addition, our studies demonstrated that norbornene homopolymers were less potent if the polymer lengths were shortened to 10-mers or 50-mers; AE activation with shorter polymers decreased significantly or required substantially higher polymer concentrations to be efficacious.^11,13^ For these reasons, polynorbornene 100-mers were the focus of these studies. We hypothesized that the ligands on each end of the 100-mers were likely contributing to AE induction. Moreover, we rationalized that the presence of activating sugars at the ends of the polymers was paramount to the activation of AE because polymers with 100 repeating units exhibited maximal activation compared with lower valency polymers. To test this hypothesis, we sought to synthesize glycopolymer probes that would allow us to determine the roles of ligand density and ligand architecture in inducing AE.

When designing the glycopolymer probes, two important features of the polymers were considered: the identity of the sugars and the arrangement of the ligands. As previously stated, mannose and fucose moieties paired with a polynorbornene backbone were found to be potent inducers of AE in mouse sperm; in particular, when tested over a wide range of polymer concentrations, these sugars were shown to have the highest AE% along with the lowest EC_50_ values.^12^ As a result, mannose and fucose were designated as the activating sugars while glucose was employed as a nonactivating sugar because it does not induce AE in mouse sperm.^11,12,18^ In addition to the identity of the sugars, the placement of these moieties was considered. Our goal was to prepare sequence-specific polymers that would elucidate the polymer architecture necessary for the activation of AE as well as provide additional information about the receptors on the sperm involved in AE. In this case, block copolymers prepared by ROMP would provide appropriate control over the order and number of ligands in the sequence as block copolymers synthesized by ROMP have been shown to have controlled molecular weights and low polydispersity indices.^19−22^ Because we had previously observed a decrease in AE activation when the polymers were shortened to 50-mers, a 50:50 diblock copolymer was not the ideal approach for this study. Our interest was in the contributions of each end of the polymers on AE activation; hence, a barbell-like sequence was the optimal design for our purposes. A triblock (ABA or ABC) design was utilized where an activating sugar (A, C) comprised each end block of the polymer, and the center block (B) was constituted with the nonactivating sugar (Figure 2C,D). By varying the lengths of the end blocks of the polymer, we were able to probe two questions: whether AE was reliant on the simultaneous activation of multiple receptors and the approximate distance required between activating ligands for optimal induction. To confirm our hypothesis that AE activation by glycopolymers was reliant on the block sequence of sugars, random copolymers consisting of 20% of an activating ligand (Figure 2B) were tested.

The purities of the acetylated glycopolymers were confirmed by ^1^H NMR and ^13^C NMR spectroscopy. The degree of polymerization (DP_n_) of each polymer was calculated by end-group analysis of the aromatic protons of the styrene group at δ 7.33 ppm in CDCl_3_. The number-average (*M*_n_), weight-average (*M*_w_) molecular weights, and dispersities (*Đ*_M_) were determined by GPC with polystyrene as a standard (Table S1), which can lead to an underestimation of molecular weights. The glycopolymers each had low to moderate molecular weight dispersities ranging from 1.1 to 1.4. Once characterized, the polymers were deacetylated, and their purity was determined by ^1^H NMR spectroscopy. Finally, conformations of the polymer structures in the buffer solution were determined by SAXS analysis (Table 1).

**Table 1:** SAXS Data Fit Parameters for 1% (w/v) Norbornene Homopolymers, Random Copolymers, and Block Copolymers Fit to a Flexible Cylinder Model

polymer	contour length, *L* (Å)	Kuhn length, 2*l*_p_ (Å)	radius, *R* (Å)
poly(**1a**)_100_ (I)^a^^,^^b^	396.8 ± 11.9	29.4 ± 1.7	8.3 ± 0.1
poly(**1b**)_100_ (II)^a^	448.5 ± 10.9	46.3 ± 2.4	8.9 ± 0.1
poly(**1a**_20_-*ran*-**1c**_80_) (II)	425.3 ± 47.5	54.1 ± 5.6	9.1 ± 0.4
poly(**1b**_20_-*ran*-**1c**_80_) (II)	539.5 ± 51.7	34.5 ± 5.2	10.1 ± 0.8
poly(**1a**)_5_-*block*-poly(**1c**)_90_-*block-*poly(**1a**)_5_ (I)	885.3 ± 64.9	42.6 ± 3.4	9.1 ± 1.0
poly(**1a**)_10_-*block*-poly(**1c**)_80_-*block-*poly(**1a**)_10_ (I)	1198.8 ± 63.3	41.4 ± 3.7	9.7 ± 0.3
poly(**1a**)_10_-*block*-poly(**1c**)_80_-*block-*poly(**1b**)_10_ (I)	1164.3 ± 8.9	37.7 ± 0.4	9.1 ± 0.03
poly(**1a**)_20_-*block*-poly(**1c**)_60_-*block-*poly(**1a**)_20_ (I)	941.8 ± 12.8	34.4 ± 0.6	8.5 ± 0.07
poly(**1b**)_5_-*block*-poly(**1c**)_90_-*block-*poly(**1b**)_5_ (I)	995.5 ± 31.5	42.0 ± 2.5	9.1 ± 0.2
poly(**1b**)_10_-*block*-poly(**1c**)_80_-*block-*poly(**1b**)_10_ (III)	841.1 ± 143.7	43.4 ± 7.7	9.9 ± 1.5
poly(**1b**)_10_-*block*-poly(**1c**)_80_-*block-*poly(**1a**)_10_ (I)	987.0 ± 72.5	40.3 ± 3.2	8.5 ± 0.3
poly(**1b**)_20_-*block*-poly(**1c**)_60_-*block-*poly(**1b**)_20_ (I)	829.1 ± 17.6	33.4 ± 1.0	8.5 ± 0.1

a Data for these homopolymers are from a previous study.^13^

b (I), (II), and (III) denote the polymer batch.

### AE Activation in Mouse Sperm with Homopolymers

Triblock copolymers were prepared with activating ligands, mannose or fucose, and a nonactivating ligand, glucose. The activating end block sizes varied from 5 to 20 repeating units; the nonactivating center block ranged from 60 to 90 repeat units to maintain the polymer degree of polymerization at 100. All glycopolymers were tested at polymer concentrations of 0.1, 1, 5, 10, and 20 μM with capacitated mouse sperm using a triple-stain flow cytometry assay. In the case of some polymers, concentrations as low as 0.0001 μM were tested to determine the lowest concentration at which AE induction was no longer observed. Polymer concentrations above 20 μM were not tested because a decrease in AE% as well as cell viability was observed at higher concentrations. A decrease in AE activity at higher concentrations is likely due to an increase in competing monovalent and multivalent interactions as previously described.^12^

**Figure 3:**
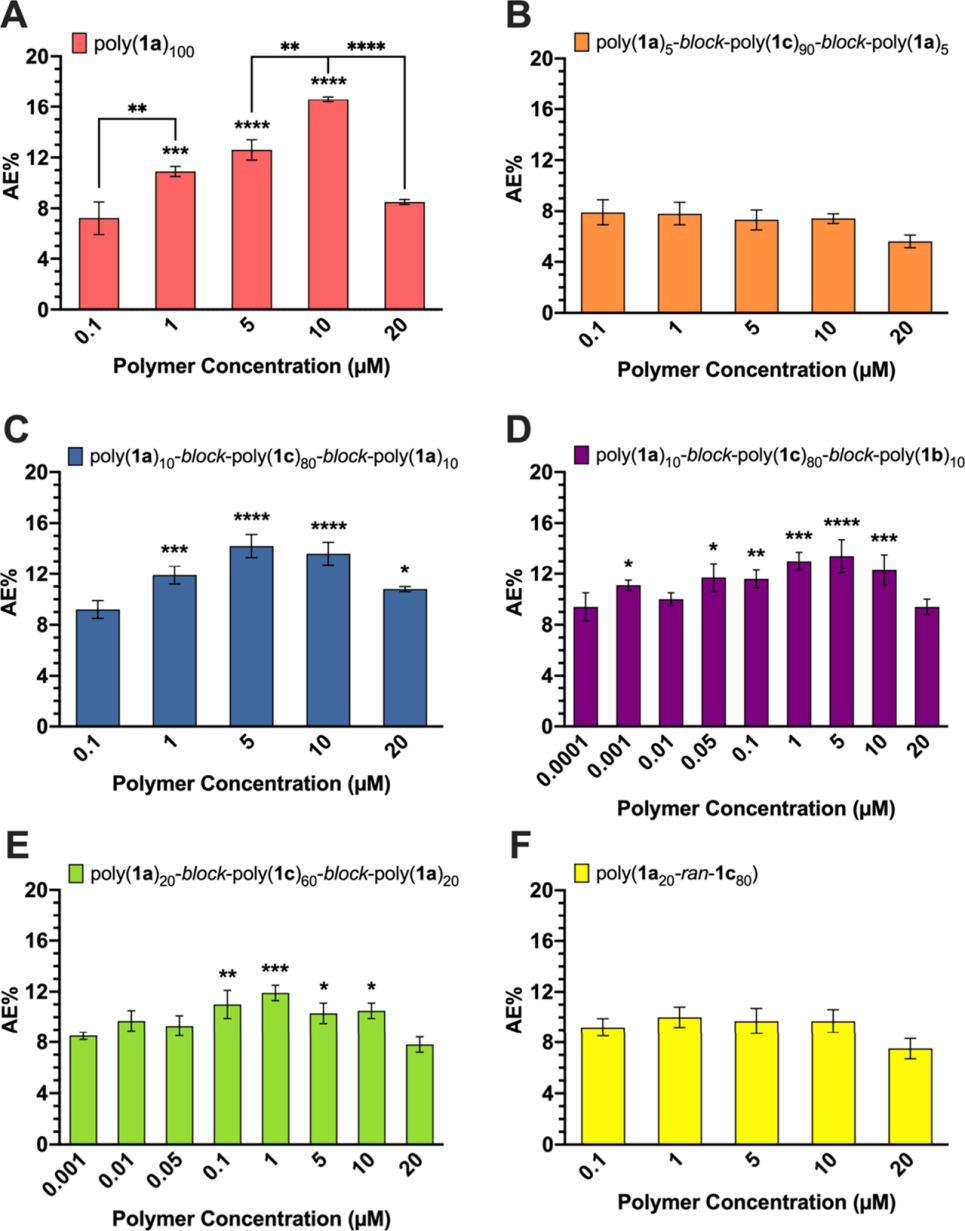
AE induction in mouse sperm by (A) homopolymers, (B–E) triblock copolymers, or (F) random copolymer displaying mannose and glucose moieties. The average AE% for mouse sperm treated with DPBS (negative control) was 7.2%. Data represent mean ± standard error of the mean of at least three independent experiments testing at least two batches of each polymer. One-way ANOVA was used to compare AE% of glycopolymer induction to DPBS and AE% of consecutive polymer concentrations. *p < 0.05, **p < 0.01, ***p < 0.001, ****p < 0.0001 for all comparisons.

**Figure 4:**
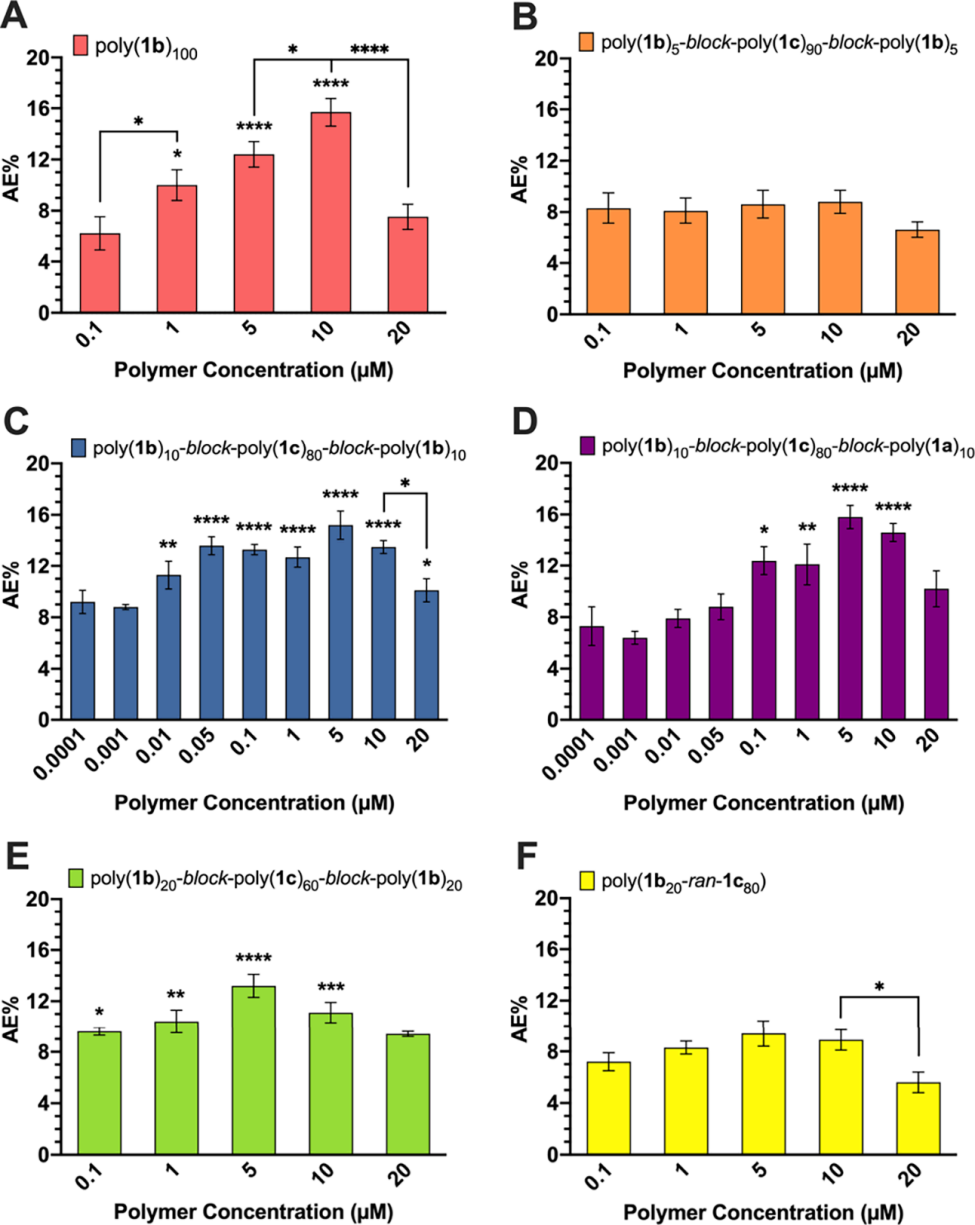
AE induction in mouse sperm by (A) homopolymers, (B–E) triblock copolymers, or (F) random copolymer displaying fucose and glucose moieties. The average AE% for mouse sperm treated with DPBS (negative control) was 7.2%. Data represent mean ± standard error of the mean of at least three independent experiments testing at least two batches of each polymer. One-way ANOVA was used to compare AE% of glycopolymer induction to DPBS and AE% of consecutive polymer concentrations. *p < 0.05, **p < 0.01, ***p < 0.001, ****p < 0.0001 for all comparisons.

Dulbecco’s phosphate-buffered saline (DPBS) was used as a negative control and was found to induce spontaneous AE in only 7.2% of mouse sperm. As a secondary negative control, poly(**1c**)_100_ was prepared and showed no significant difference when compared to DPBS (Figure S1), as expected. As consistently shown in our past studies,^11,12,18^ polynorbornene 100-mers displaying fucose or mannose moieties induced AE in mouse sperm in a concentration-dependent manner; an increase in AE% was observed when the concentration of polymer was increased. Treatment of mouse sperm with the homopolymers poly(**1a**)_100_ and poly(**1b**)_100_ resulted in a maximum AE% of approximately 17% at 10 μM (Figures 3A and 4A) and has been shown to activate AE comparably to well-known chemical inducers such as calcium ionophore A23187.^11,12,18^ These homopolymers were used as positive controls to establish the relative efficacy of the random and block copolymers in inducing AE in mouse sperm.

### Effects of Block Size on AE Activation in Mouse Sperm with a Single Activating Sugar in Triblock Copolymers

Mouse sperm treated with polymers containing two 5-mer blocks of activating sugars, poly(**1a**)_5_-*block*-poly(**1c**)_90_-*block*-poly(**1a**)_5_ and poly(**1b**)_5_-*block*-poly(**1c**)_90_-*block*-poly(**1b**)_5_, did not undergo AE at any polymer concentration. When compared with DPBS, no significant difference in AE% was observed (Figures 3B and 4B). Interestingly, when the block size was increased to 10, for example, poly(**1a**)_10_-*block*-poly(**1c**)_80_-*block*-poly(**1a**)_10_ and poly(**1b**)_10_-*block*-poly(**1c**)_80_-*block*-poly(**1b**)_10_, AE activation occurred (Figures 3C and 4C). Treating sperm with these block copolymers resulted in AE activation that was comparable to that of their mannose or fucose homopolymers (Figure S2). Additionally, the maximum AE% for poly(**1a**)_10_-*block*-poly(**1c**)_80_-*block*-poly(**1a**)_10_ and poly(**1b**)_10_-*block*-poly(**1c**)_80_-*block*-poly(**1b**)_10_ was achieved at a lower concentration than that with the homopolymers. When 10 μM poly(**1a**)_100_ or poly(**1b**)_100_ was compared to 5 μM of their respective block copolymers, AE activity was comparable (Figure S3), suggesting that these triblock copolymers were more potent than the homopolymers because they required half of the polymer concentration to activate AE to the same degree. Surprisingly, poly(**1b**)_10_-*block*-poly(**1c**)_80_-*block*-poly(**1b**)_10_ demonstrated a different activation profile. With poly(**1a**)_100_, poly(**1b**)_100_, and poly(**1a**)_10_-*block*-poly(**1c**)_80_-*block*-poly(**1a**)_10_, a decrease in AE activity as polymer concentration dropped below 1 μM was observed. However, poly(**1b**)_10_-*block*-poly(**1c**)_80_-*block*-poly(**1b**)_10_ activated AE at concentrations as low as 0.01 μM, which suggests that this polymer is a potent inducer of AE as it requires 100-fold less polymer than its counterparts.

Finally, copolymers comprising two blocks of 20 activating sugar ligands were tested with mouse sperm. Interestingly, poly(**1a**)_20_-*block*-poly(**1c**)_60_-*block*-poly(**1a**)_20_ and poly(**1b**)_20_-*block*-poly(**1c**)_60_-*block*-poly(**1b**)_20_ were able to induce AE in mouse sperm but were not as potent as the homopolymers or the polymers displaying two 10-mer activating sugar blocks. There was no significant difference observed between the aforementioned polymers until a 10 μM polymer concentration was reached; at 10 μM, the polymers consisting of two 20-mer blocks had a significantly lower AE% than their respective homopolymers (Figure S2). Because the maximum AE% for poly(**1a**)_20_-*block*-poly(**1c**)_60_-*block*-poly(**1a**)_20_ and poly(**1b**)_20_-*block*-poly(**1c**)_60_-*block*-poly(**1b**)_20_ was obtained at 5 μM (Figures 3D and 4D), this concentration was used to compare to the maximum AE% of the homopolymers at 10 μM. Although AE activity with 5 μM of poly(**1b**)_20_-*block*-poly(**1c**)_60_-*block*-poly(**1b**)_20_ was comparable to 10 μM of poly(**1b**)_100_, this was not the case with poly(**1a**)_20_-*block*-poly(**1c**)_60_-*block*-poly(**1a**)_20_ and poly(**1a**)_100_ (Figure S3). Overall, the triblock copolymers displaying two 10 activating sugar blocks are the most effective barbell sequences, as the maximum AE% obtained is most comparable to that with homopolymer controls.

### Effects of Ligand Density in Random Copolymers on AE Activation in Mouse Sperm

To further validate our results that blocks of 10 repeating activating ligands on each end of the glycopolymer are imperative for efficient activation of AE, random copolymers were synthesized. Since the block copolymers consisting of 20% activating sugars were the most effective, poly(**1a**_20_-*ran*-**1c**_80_) and poly(**1b**_20_-*ran*-**1c**_80_) were prepared with 20% of a single activating sugar and 80% glucose, the nonactivating sugar. These random copolymers were not sequence-specific, and therefore, the exact arrangement of sugars in the polymers was unknown. However, the rates of monomer incorporation were considered to ensure that no accidental blocks formed during synthesis, resulting in a pseudoblock copolymer. The incorporation of mannose and glucose monomers or fucose and glucose monomers into the polymer sequence was quantified by the ^1^H NMR proton integration of each monomer after the polymerization was run to approximately 20–30% completion. The ratios of activating sugar to nonactivating sugar used in the syntheses were 1:1 and 1:4. We found that even at 30% completion, the ratios of the monomers were still close to 1:1 or 1:4, suggesting that the monomer incorporation rates were similar and there was a decreased risk of block formation during the random copolymerization (Figures S4–S7).

Treatment of mouse sperm with poly(**1a**_20_-*ran*-**1c**_80_) and poly(**1b**_20_-*ran*-**1c**_80_) demonstrated that random copolymers did not activate AE. When compared to DPBS, there was no significant difference in AE% at any of the polymer concentrations tested (Figures 3F and 4F). It was evident that poly(**1a**_20_-*ran*-**1c**_80_) and poly(**1b**_20_-*ran*-**1c**_80_) were significantly less effective at activating AE than their homopolymer or block copolymer counterparts, with the exception of poly(**1a**)_5_-*block*-poly(**1c**)_90_-*block*-poly(**1a**)_5_ and poly(**1b**)_5_-*block*-poly(**1c**)_90_-*block*-poly(**1b**)_5_ (Figure S2). Therefore, these results further support the hypothesis that a specific block arrangement and ligand density of activating sugars within the polymer are crucial features that contribute to successful glycopolymer-induced AE.

### Effects of Ligand Density in Triblock Copolymers on AE Activation in Mouse Sperm with Two Activating Sugars

Since the barbell sequence consisting of two 10-mer blocks of activating sugars had the most similar capability for AE activation to the homopolymers, we continued to explore different polymer arrangements while maintaining the same barbell template. Specifically, two additional polymers were synthesized with 10 repeat units of mannose ligands on one end and fucose ligands on the other (Figure 2D) to afford glycopolymers poly(**1a**)_10_-*block*-poly(**1c**)_80_-*block*-poly(**1b**)_10_ and poly(**1b**)_10_-*block*-poly(**1c**)_80_-*block*-poly(**1a**)_10_ with identical but reverse block orders. The two orders of block formation were used to account for any discrepancies in the number of repeat units within each block that could arise during synthesis and possibly result in differences in AE activation.

Surprisingly, poly(**1a**)_10_-*block*-poly(**1c**)_80_-*block*-poly(**1b**)_10_ and poly(**1b**)_10_-*block*-poly(**1c**)_80_-*block*-poly(**1a**)_10_ followed the same activation trend as the block copolymers consisting of a single activating sugar. Moreover, the activation was independent of the block synthesis order. AE activation for poly(**1a**)_10_-*block*-poly(**1c**)_80_-*block*-poly(**1b**)_10_ and poly(**1b**)_10_-*block*-poly(**1c**)_80_-*block*-poly(**1a**)_10_ was observed at polymer concentrations as low as 0.05 and 0.1 μM, respectively (Figures 3D and 4D). Levels of AE activation were comparable for homopolymers and these mixed triblock copolymers at a majority of polymer concentrations (Figure S2). There was no statistical difference between the AE% of poly(**1**)_100_ at 10 μM and the respective mixed triblock copolymers at 5 μM (Figure S3). In conclusion, the potency of the triblock copolymers with two 10-mer blocks of activating sugars is greater than that of the homopolymers because a lower polymer concentration is required for maximal AE.

### Structure of the Glycopolymer Backbones

Based on the results of the biological assays, we wanted to validate the reason behind the differences in AE activation among the block and random copolymer arrangements. We hypothesized that because the polymer backbones and degree of polymerization were conserved, there would not be significant differences between the polymer structures. To ascertain information about the glycopolymer structures in cell media, SAXS experiments were conducted.

SAXS data were collected on deacetylated polymer solutions at 1% (w/v) in M16 medium, which was the same buffer used in the biological assays. However, because BSA is a protein that scatters X-rays, the buffer was not supplemented with BSA to avoid interference. M16 buffer was used for the background signal of the SAXS experiments, and this signal was subtracted from the data collected. Data for all of the polymers was collected in a *q* range of 0.005 to 3.19 Å^–1^; however, samples were fit in a *q* region of 0.005–0.4 A^–1^. This range was chosen because this is the relevant SAXS scattering window. Although error in the majority of the SAXS region is negligible (<1%), uncertainties in the WAXS region, such as at *q* greater than 1.0 Å^–1^, are larger because the scattering is dominated by the signal from water. In addition, the signal from the background scattering is very large for the *q* range below 0.01 Å^–1^, resulting in greater uncertainties after background subtraction in comparison to those of other regions. The low-*q* region corresponds to longer scale orientations present in the samples and, consequently, increases the error in the lengths of the fitted models particularly.

**Figure 5:**
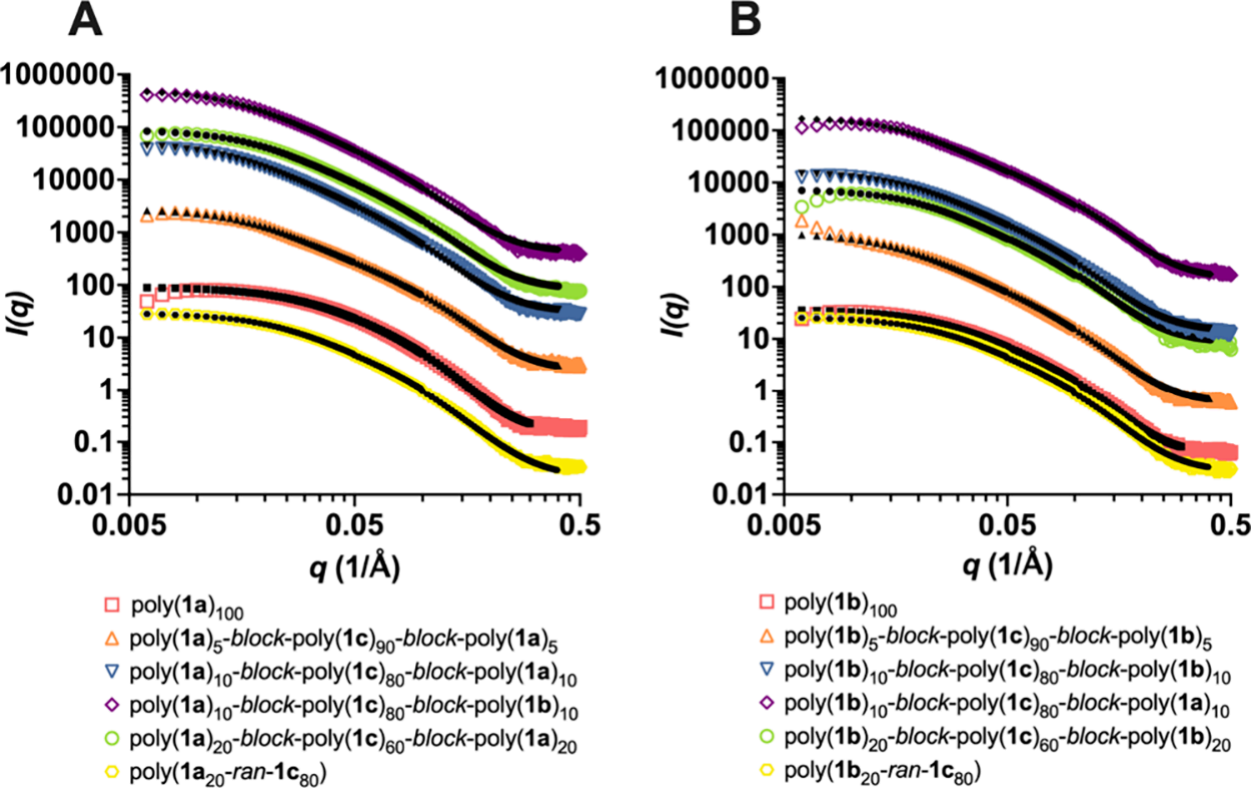
SAXS plots of (A) mannose glycopolymers and (B) fucose glycopolymers at 1% (w/v) in M16 buffer. The glycopolymers were fitted to a flexible cylinder model. Fits encompassed data where the scattering is above the background. Each data set was offset by an arbitrary amount for the sake of clarity. The color traces represent the glycopolymer data, whereas the black traces correspond to the fits to the data.

**Figure 6:**
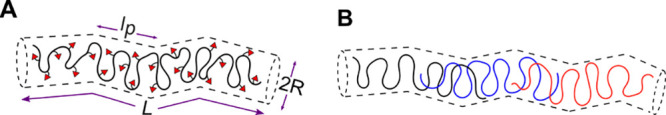
Proposed solution structure consistent with the SAXS model of norbornene homopolymers, block copolymers, and random copolymers. (A) According to SAXS data analysis, the glycopolymers adopt a flexible cylinder conformation where *L* is the contour length, *R* is the radius and 2*l*_p_ is the Kuhn length. (B) Proposed conformation of small entangled chains of irregularly coiled block copolymers in a flexible cylinder model. The black, blue, and red lines correspond to separate polymer chains. The aggregation of polymer chains could account for the longer contour lengths observed in the block copolymers when compared with the homopolymers or random copolymers. Sugar ligands were removed for clarity.

The scattering and fit for the polynorbornene samples displaying fucose or mannose sugars are shown in Figure 5. Based on the shapes of the curves, the data was fit to a flexible cylinder model (Figure 6A), which is consistent with our previously published work on norbornene polymers.^18^ The flexible cylinder model approximates parameters for semirigid structures^23^ and has been used as a model for fitting SAXS data from polymers with norbornene-based backbones.^24,25^ The flexible cylinder model consists of three parameters: contour length, Kuhn length, and radius. The contour length, *L*, is the overall length of the polymer chain. The Kuhn length, 2*l*_p_, is the length of repeat segments in the polymer. The radius, *R*, denotes the circular face of the cylinder.^23^ Dispersity of the radius and Kuhn length was added as a Gaussian distribution about the parameter value.^26^ The results for the parameters of each norbornene glycopolymer are summarized in Table 1. The error for each parameter was determined in SasView by monitoring the Chi-squared value for the fit.

Based on the parameters presented in Table 1, all of the polymers have very similar conformations despite varying ligand displays. The low-*q* region can provide information about the overall size of the polymer, mid-*q* describes the stiffness of the polymer, and the high-*q* region is related to the cross-sectional size.^23,27^ Therefore, the high-*q* and mid-*q* regions can be used to determine information about the radii and Kuhn lengths, respectively, while the low-*q* region can be used to ascertain contour lengths. The radii of the polymers appear well-defined and retained across the different samples, as all of the polymers have radii very close to 1.0 nm. This is further confirmed when observing the slopes of the data in the high-*q* region (Table S4); for all of the polymers, the slopes ranged from 2.7 to 3.0. Those data indicate that the short-range conformations of the polymers are similar regardless of the architecture of the ligands. However, when the other length parameters are analyzed, some disparities are observed.

When compared to poly(**1**)_100_, the block copolymers appear to possess longer contour lengths that are approximately 2–3 times greater than the homopolymers and random copolymers. In particular, the triblock copolymers consisting of two activating sugars, poly(**1a**)_10_-*block*-poly(**1c**)_80_-*block*-poly(**1b**)_10_ and poly(**1b**)_10_-*block*-poly(**1c**)_80_-*block*-poly(**1a**)_10_, show a greater increase in intensity at lower *q* values. This trend is also present in the data for poly(**1b**)_5_-*block*-poly(**1c**)_90_-*block*-poly(**1b**)_5_ and poly(**1a**)_10_-*block*-poly(**1c**)_80_-*block*-poly(**1a**)_10_. A greater increase in intensity at low *q* indicates the presence of longer-scale interactions. When fit to a flexible cylinder model, this results in increased contour lengths. Because all of the glycopolymers shared the same backbone and degree of polymerization, these results were surprising as there would be no reason expected for the polymers to vary in length. In addition, the molecular weights and the degree of polymerization for the block copolymers are comparable to those of the homopolymers and random copolymers (Table S1). Therefore, this significant difference in contour lengths could be due to errors introduced during background subtraction, which is greater at low-*q* or aggregation of the polymers.

The ability of block copolymers to form assemblies and adopt various morphologies has been well-studied and exploited in the fabrication of photonic materials, diagnostic imaging, and drug delivery and release.^28−31^ In the case of these norbornene glycopolymers, the presence of hydroxyl and amide groups likely drives the formation of hydrogen bonds and intermolecular interactions that can contribute to their overall conformations. Specifically, these polymers will most likely fold into irregular coils that can be intertwined with one another to form assemblies. The literature has shown that polymer conformations will vary depending on the length of the side chains and the presence of bulky substituents. In particular, densely packed, bulky functional groups decrease chain entanglement while less bulky substituents show an increase in entanglement.^32−34^ Additionally, studies on polysaccharides, norbornene-based polymers, and other polymer structures have demonstrated that single coils of these structures can become entangled to form assemblies of intertwining chains and in some cases helices.^35−37^ Based on the longer contour lengths and the relatively small, uncrowded functional groups, these polymers may assume structures in which multiple polymer chains are entangled with one another, resulting in longer contour lengths than expected, with radii similar to those of the homopolymers (Figure 6B). Nevertheless, the polymers that appear to have more extended contour lengths have significantly larger errors for this parameter, indicating that they may, in reality, be much closer in length to the homopolymers as would be expected. Therefore, the contour lengths of the block copolymers should be interpreted with caution.

Regardless of the discrepancies in contour lengths, the third length parameter, the Kuhn length, appeared to be very similar among the samples. The Kuhn lengths, which can translate to the rigidity of the structure in the flexible cylinder model, ranged from 2.9 to 5.4 nm. This suggests that the polymers have a relatively rigid conformation and are comparable to one another. In our previous studies,^13,18^ we have shown the importance of polymer rigidity in the activation of AE in mouse sperm. By employing different polymer backbones, we previously demonstrated that the rigidity of the polynorbornene backbone is necessary for the efficient activation of AE. In the case of the homopolymers and copolymers, there was no significant difference in the Kuhn lengths of the samples. This confirms that the arrangement of the ligands did not significantly change the rigidity of the polymer structures. Overall, the SAXS data analysis demonstrated that each sample displayed parameters corresponding to coiled polymer orientations with similar sizes, shapes, and extended rigid regions. These data are consistent with our hypothesis that varying arrangements and ligand display do not affect the overall conformations of the polymer structures. Therefore, these data confirm that specific glycopolymer architectures are, in fact, responsible for the induction of AE in mouse sperm.

### Implications for Sperm Receptor Behavior in Glycopolymer-Induced AE

The results of these experiments raise interesting questions about the receptor function contributing to mouse sperm AE. In particular, these results suggest that AE is influenced by a combination of receptor mechanisms. Multivalent ligands synthesized by ROMP have been shown to act as excellent effectors or inhibitors of biological activity through the clustering of receptors.^38−41^ During receptor clustering, the orientation or proximity of receptors on the cell surface is altered by the presence of a multivalent ligand to enhance binding affinity and elicit a biological response.^42,43^ Studies utilizing linear norbornene-based glycopolymers have demonstrated that the rate of receptor clustering and proximity of receptors may increase with polymers of higher valency, resulting in increased potency.^39,41,44^ This phenomenon supports our previous studies demonstrating that shorter polynorbornene homopolymers consisting of 10 or 50 repeat units were not as effective at inducing AE when compared to homopolymers with a greater degree of polymerization.^11,13^

However, block copolymers with two 20-mer blocks of activating sugars demonstrated a reduction in potency when compared to homopolymers with 100 repeat units or block copolymers with two 10-mer blocks of activating ligands, suggesting that additional receptor mechanisms are at play. Herein, we propose that AE is successfully induced when a glycopolymer can bind to two receptors on the sperm head simultaneously, recruiting chelation and statistical rebinding for activation. During chelation, a multivalent ligand is able to bind to multiple receptors resulting in greater affinity once the first ligand binds, ultimately decreasing the dissociation rate.^45,46^ Typically, chelation by a multivalent ligand acts as a bridge for multiple receptors on a cell surface to induce a signal. Therefore, if the receptors on the sperm head are a specific distance apart, then a multivalent ligand that spans the distance of the receptors is necessary for effective binding. During statistical rebinding, the presence of the multivalent ligand increases the local concentration near the receptors, resulting in increased binding affinity.^42,47^ These receptor behaviors are supported by the activity of the block copolymers.

**Figure 7:**
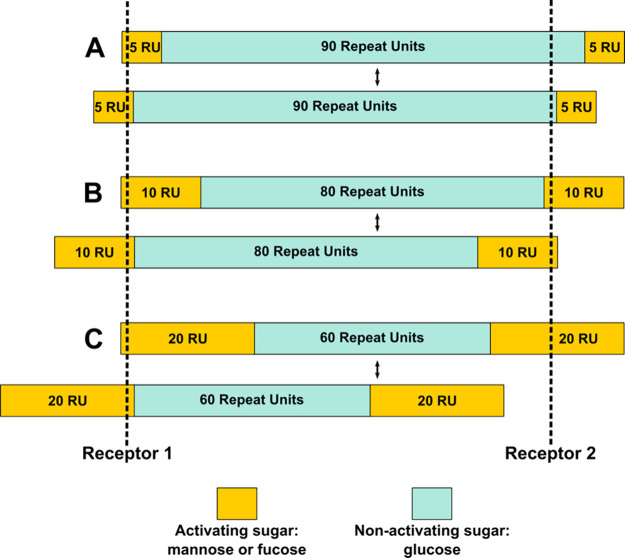
Simplified binding model for norbornene block copolymers. The binding model illustrated is only one depiction of a possible binding interaction between the glycopolymers and sperm-surface receptors and is not exhaustive of all of the potential binding modalities or side interactions. The blue blocks represent repeat units of a nonactivating ligand (glucose), while the gold blocks correspond to repeat units of an activating ligand (mannose or fucose). RU signifies repeating units. We propose that the glycopolymers must simultaneously recruit two receptors on the sperm to activate AE. (A) Block copolymers with two 5-mer blocks of activating sugars cannot successfully span the distance of the two receptors, resulting in no AE activity. (B) Block copolymers consisting of two 10-mer blocks of activating ligands can span the distance of the receptors regardless of the initial binding and rebinding position of the activating ligand, resulting in AE. (C) Block copolymers comprising two 20-mer blocks of activating sugars can span the distance of the receptors and activate AE when the polymer binds via the first few activating sugars in the block. However, if the polymer binds via the last few activating sugars, the polymer becomes too short, and AE activity is reduced.

Despite the degree of polymerization remaining constant among the block copolymers, the orientation or length of the polymer bridges varies, resulting in the observed differences in AE activation. For example, a block copolymer with two 5-mer blocks does not possess the correct number of repeat units of activating sugars to span the distance of the two receptors, resulting in no activation of AE (Figure 7A). Conversely, depending on the position at which the polymer initially binds and rebinds to the first receptor, a block copolymer with two 20-mer blocks of activating ligands may successfully span the distance of the receptors, resulting in efficient binding and AE activation, or may become too short to recruit both receptors. As a consequence, there is a decrease in activation efficiency (Figure 7C). Interestingly, a 100-mer with blocks of 10 repeat units on each end possesses the optimal architecture for AE activation in mouse sperm; regardless of the possible initial binding and rebinding positions of the polymer to the first receptor, this polymer can recruit both receptors and initiate AE (Figure 7B). This behavior is further corroborated by the inability of random copolymers to induce AE. Because the placement of ligands was randomized in these copolymers, the distance of the polymer bridge was not controlled or optimized to span the distance of the receptors, leading to no activation and presumably inefficient binding. Although AE activation by the block and random copolymers provides evidence to support the chelate effect, this may not be the only binding interaction occurring between the glycopolymers and sperm-surface receptors. Therefore, the previously described binding model (Figure 7) is only a single depiction of glycopolymer-induced AE and is not all-encompassing of the numerous binding behaviors and possibilities.

## Conclusions

Triblock polynorbornene copolymers consisting of two 10-mer blocks of mannose or fucose ligands separated by a block of nonactivating sugar, glucose, are able to induce AE in mouse sperm at levels comparable to mannose or fucose polynorbornene homopolymers. In contrast, random copolymers and block copolymers comprised of two 5-mer or 20-mer blocks of mannose or fucose ligands with nonactivating glucose as a spacer demonstrated no AE activation or reduced potency. The SAXS data analysis revealed that the glycopolymers all adopted semiflexible cylinder conformations with very similar radii and rigidity.

Overall, our results highlight the sugar arrangement in glycopolymers required for efficient AE activation in mouse sperm. The behavior of the ABC triblock glycopolymers raises a question about the receptors participating in glycopolymer-induced AE. Mannose and fucose moieties on the same block copolymer were able to induce AE in mouse sperm, suggesting the presence of receptors on the sperm that can recognize both sugars. It is unclear whether the same receptor is able to interact with both sugars or if two different receptors in close proximity bind to a specific sugar. Further exploration is necessary to elucidate the identities of the receptors involved in glycopolymer-induced AE and the receptor binding mechanisms that contribute to activation. The results of this study suggest that multiple polymer behaviors such as chelation, receptor clustering, and statistical rebinding are at play in the activation mechanism of AE. The structure of these block copolymers will be advantageous in future experiments utilizing block copolymers to probe the identity of the receptors and to map their location and their involvement in the transduction of signaling pathways contributing to the induction of AE by carbohydrate recognition.

## Supporting Information Available

Glycopolymer AE% comparisons and SAXS analysis; reaction schemes, NMR spectra, and GPC results (PDF)
