# Supplementary material for: Triblock Glycopolymers with Two 10-mer Blocks of Activating Sugars Enhance the Activation of Acrosomal Exocytosis in Mouse Sperm
Source: ACS Bio Med Chem Au. 2024 Apr 29;4(3):165–77. doi: 10.1021/acsbiomedchemau.4c00012 (PMC11191571; doi:10.1021/acsbiomedchemau.4c00012)
Supplement: Supplementary file 1 — bg4c00012_si_001.pdf [file bg4c00012_si_001.pdf]

**Supporting Information**

# Triblock Glycopolymers with Two 10-mer Blocks of Activating Sugars Enhance the Activation of Acrosomal Exocytosis in Mouse Sperm

Luz C. Mendez[1], Mitchell Kennedy[1], Surita R. Bhatia[1], and Nicole S. Sampson*[1,2]

[1]Department of Chemistry, Stony Brook University, Stony Brook, NY 11794-3400, United States

[2]Department of Chemistry, University of Rochester, Rochester, NY 14627-0216, United States

*Corresponding author: nicole.sampson@rochester.edu

## Table of Contents


Scheme S1. Synthesis of random copolymers ........ S4

Scheme S2. Synthesis of block copolymers........ S5

Table S1. Dispersities of Acetylated Norbornene Homopolymers, Random Copolymers, and Block Copolymers ........ S6

Table S2. Comparison of AE Induction in Mouse Sperm by Mannose Glycopolymers ........ S8

Table S3. Comparison of AE Induction in Mouse Sperm by Fucose Glycopolymers ........ S10

Table S4. Slopes at the High-*q* Region from SAXS Data Fit at 1% (w/v) of Norbornene Homopolymers, Random Copolymers, and Block Copolymers........ S12

Figure S1. AE activation in mouse sperm by poly(**1c**)$_{100}$ ........ S13

Figure S2. Comparison of AE induction in mouse sperm by mannose or fucose glycopolymers........ S14

Figure S3. AE% of mannose and fucose glycopolymers at 5 μM polymer concentration compared to the maximum AE% of poly(**1a**)$_{100}$ and poly(**1b**)$_{100}$ ........ S15

Figure S4. $^{1}$H NMR spectrum of **1c′** and **1a′** (1:1) incorporated into random copolymers S16

Figure S5. $^{1}$H NMR spectrum of **1c′** and **1a′** (4:1) incorporated into random copolymers........ S17

Figure S6. $^{1}$H NMR spectrum of **1c′** and **1b′** (1:1) incorporated into random copolymers........ S18

Figure S7. $^{1}$H NMR spectrum of **1c′** and **1b′** (4:1) incorporated into random copolymers........ S19

Figure S8. $^{1}$H NMR spectrum of poly(**1a′**$_{20}$-*ran*-**1c′**$_{80}$) ........ S20

Figure S9. $^{13}$C NMR spectrum of poly(**1a′**$_{20}$-*ran*-**1c′**$_{80}$) ........ S21

Figure S10. $^{1}$H NMR spectrum of poly(**1a**$_{20}$-*ran*-**1c**$_{80}$)........ S22

Figure S11. $^{1}$H NMR spectrum of poly(**1b′**$_{20}$-*ran*-**1c′**$_{80}$) ........ S23

Figure S12. $^{13}$C NMR spectrum of poly(**1b′**$_{20}$-*ran*-**1c′**$_{80}$) ........ S24

Figure S13. $^{1}$H NMR spectrum of poly(**1b**$_{20}$-*ran*-**1c**$_{80}$) ........ S25

Figure S14. $^{1}$H NMR spectrum of poly(**1a′**)$_{5}$-*bloc*k-poly(**1c′**)$_{90}$-*block*-poly(**1a′**)$_{5}$ ........ S26

Figure S15. $^{13}$C NMR spectrum of poly(**1a′**)$_{5}$-*bloc*k-poly(**1c′**)$_{90}$-*block*-poly(**1a′**)$_{5}$ ........ S27

Figure S16. $^{1}$H NMR spectrum of poly(**1a**)$_{5}$-*bloc*k-poly(**1c**)$_{90}$-*block*-poly(**1a**)$_{5}$ ........ S28

Figure S17. $^{1}$H NMR spectrum of poly(**1a′**)$_{10}$-*bloc*k-poly(**1c′**)$_{80}$-*block*-poly(**1a′**)$_{10}$ ........ S29

Figure S18. $^{13}$C NMR spectrum of poly(**1a′**)$_{10}$-*bloc*k-poly(**1c′**)$_{80}$-*block*-poly(**1a′**)$_{10}$ ........ S30

Figure S19. $^{1}$H NMR spectrum of poly(**1a**)$_{10}$-*bloc*k-poly(**1c**)$_{80}$-*block*-poly(**1a**)$_{10}$........ S31

Figure S20. $^{1}$H NMR spectrum of poly(**1a′**)$_{10}$-*bloc*k-poly(**1c′**)$_{80}$-*block*-poly(**1b′**)$_{10}$ ........ S32

Figure S21. $^{13}$C NMR spectrum of poly(**1a′**)$_{10}$-*bloc*k-poly(**1c′**)$_{80}$-*block*-poly(**1b′**)$_{10}$ ........ S33

Figure S22. $^{1}$H NMR spectrum of poly(**1a**)$_{10}$-*bloc*k-poly(**1c**)$_{80}$-*block*-poly(**1b**)$_{10}$ ........ S34

Figure S23. $^{1}$H NMR spectrum of poly(**1a′**)$_{20}$-*bloc*k-poly(**1c′**)$_{60}$-*block*-poly(**1a′**)$_{20}$ ................................ S35
Figure S24. $^{13}$C NMR spectrum of poly(**1a′**)$_{20}$-*bloc*k-poly(**1c′**)$_{60}$-*block*-poly(**1a′**)$_{20}$ ............................... S36
Figure S25. $^{1}$H NMR spectrum of poly(**1a**)$_{20}$-*bloc*k-poly(**1c**)$_{60}$-*block*-poly(**1a**)$_{20}$.................................... S37
Figure S26. $^{1}$H NMR spectrum of poly(**1b′**)$_{5}$-*bloc*k-poly(**1c′**)$_{90}$-*block*-poly(**1b′**)$_{5}$.................................. S38
Figure S27. $^{13}$C NMR spectrum of poly(**1b′**)$_{5}$-*bloc*k-poly(**1c′**)$_{90}$-*block*-poly(**1b′**)$_{5}$ ................................ S39
Figure S28. $^{1}$H NMR spectrum of poly(**1b**)$_{5}$-*bloc*k-poly(**1c**)$_{90}$-*block*-poly(**1b**)$_{5}$ ..................................... S40
Figure S29. $^{1}$H NMR spectrum of poly(**1b′**)$_{10}$-*bloc*k-poly(**1c′**)$_{80}$-*block*-poly(**1b′**)$_{10}$ ............................... S41
Figure S30. $^{13}$C NMR spectrum of poly(**1b′**)$_{10}$-*bloc*k-poly(**1c′**)$_{80}$-*block*-poly(**1b′**)$_{10}$.............................. S42
Figure S31. $^{1}$H NMR spectrum of poly(**1b**)$_{10}$-*bloc*k-poly(**1c**)$_{80}$-*block*-poly(**1b**)$_{10}$ .................................. S43
Figure S32. $^{1}$H NMR spectrum of poly(**1b′**)$_{10}$-*bloc*k-poly(**1c′**)$_{80}$-*block*-poly(**1a′**)$_{10}$ ............................... S44
Figure S33. $^{13}$C NMR spectrum of poly(**1b′**)$_{10}$-*bloc*k-poly(**1c′**)$_{80}$-*block*-poly(**1a′**)$_{10}$.............................. S45
Figure S34. $^{1}$H NMR spectrum of poly(**1b**)$_{10}$-*bloc*k-poly(**1c**)$_{80}$-*block*-poly(**1a**)$_{10}$ .................................. S46
Figure S35. $^{1}$H NMR spectrum of poly(**1b′**)$_{20}$-*bloc*k-poly(**1c′**)$_{60}$-*block*-poly(**1b′**)$_{20}$ ............................... S47
Figure S36. $^{13}$C NMR spectrum of poly(**1b′**)$_{20}$-*bloc*k-poly(**1c′**)$_{60}$-*block*-poly(**1b′**)$_{20}$.............................. S48
Figure S37. $^{1}$H NMR spectrum of poly(**1b**)$_{20}$-*bloc*k-poly(**1c**)$_{60}$-*block*-poly(**1b**)$_{20}$ .................................. S49

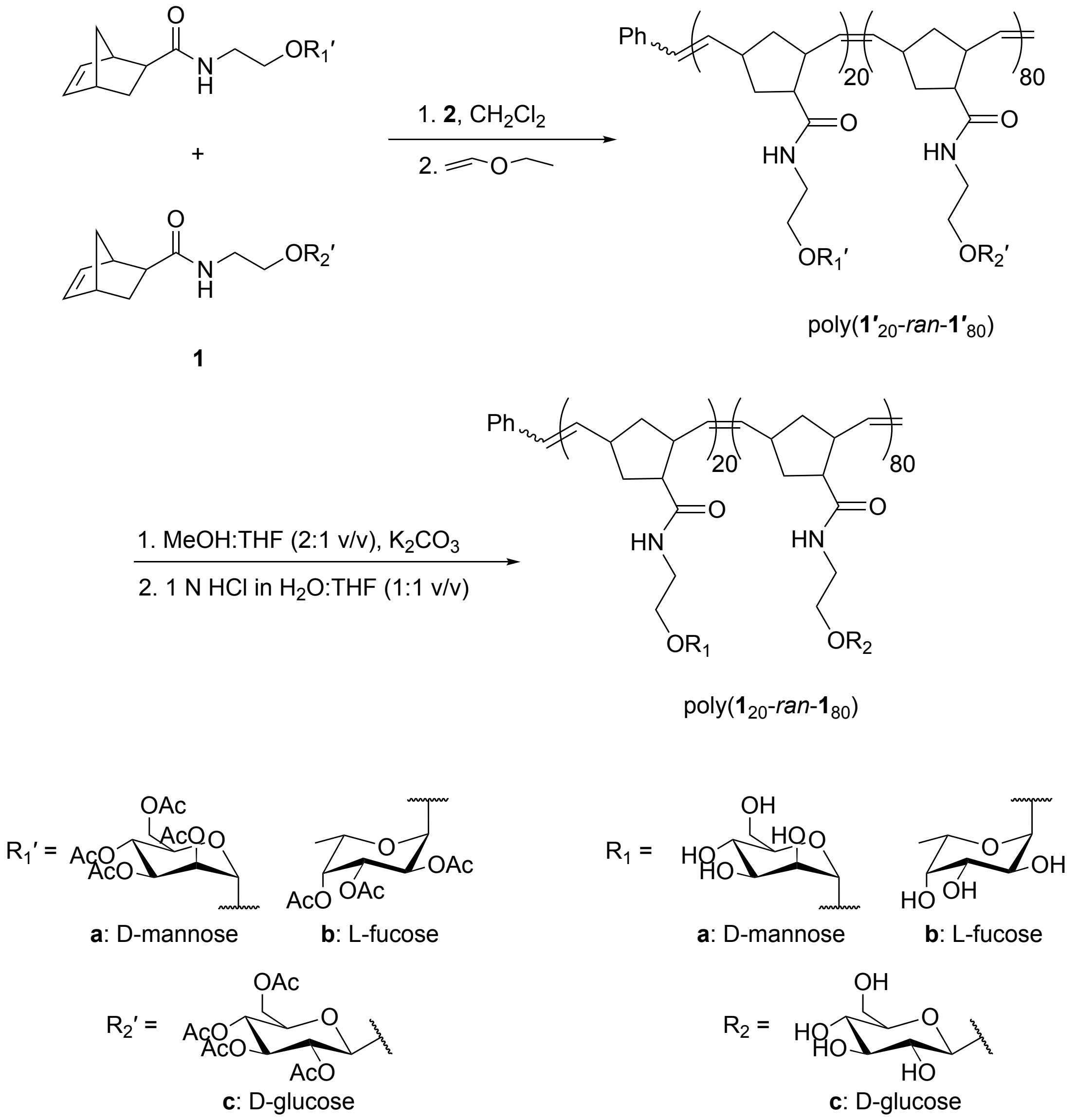


**Scheme S1.** Synthesis of random copolymers

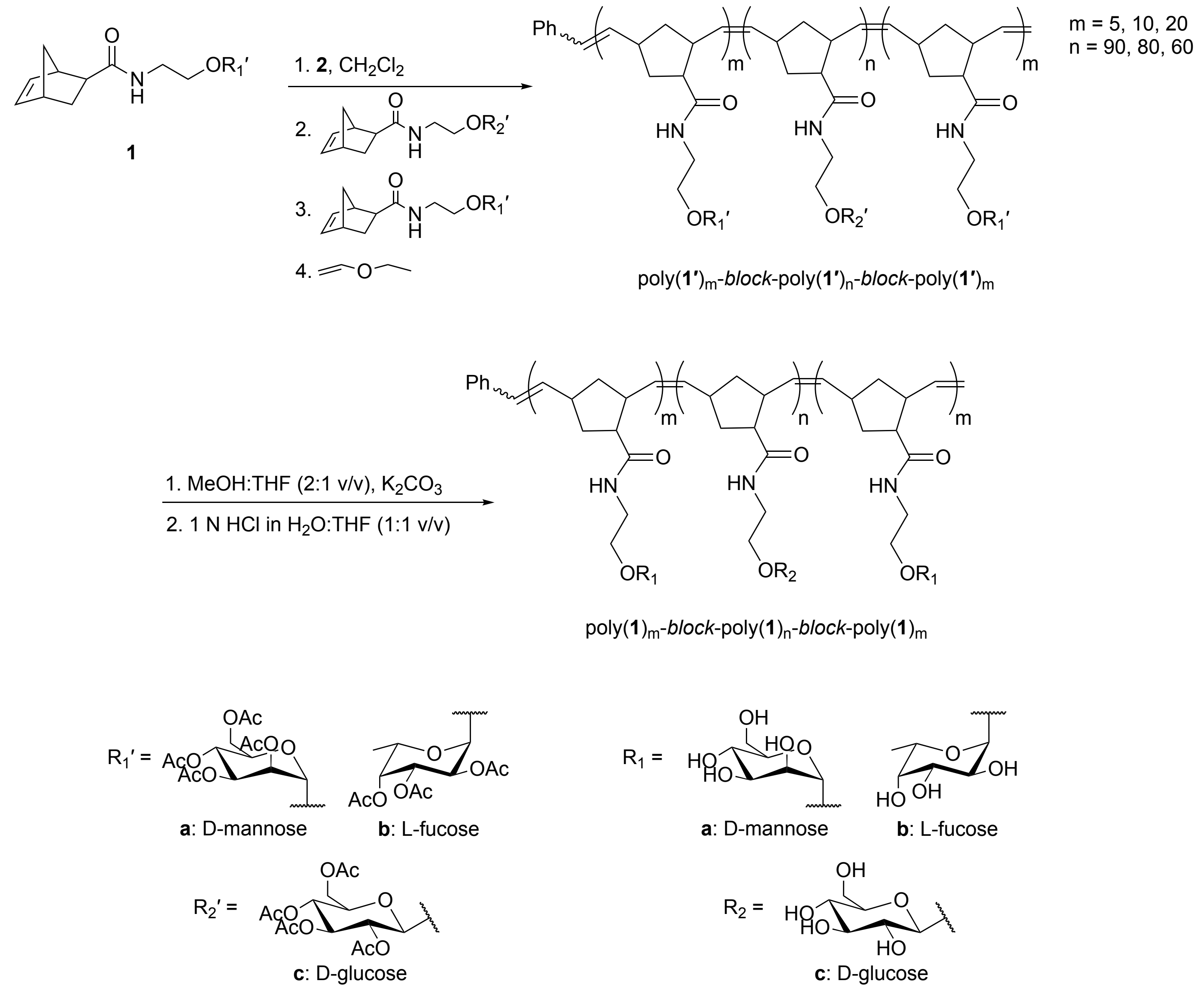


**Scheme S2.** Synthesis of block copolymers

**Table S1.** Dispersities of Acetylated Norbornene Homopolymers, Random Copolymers, and Block Copolymers

| Polymer | $M_n^{theor, a}$ (kDa) | $M_n^b$ (kDa) | $M_w^b$ (kDa) | $Đ_M^b$ (kDa) | $DP_n^c$ | **1c′**:**1a′**/**1b′** Ratio$^{theor}$ | **1c′**:**1a′**/**1b′** Ratio$^c$ |
|---|---|---|---|---|---|---|---|
| poly(**1a′**)$_{100}$ (I)$^{d, e}$ | 51,260 | 46,540 | 54,110 | 1.16 | 92 | – | – |
| poly(**1a′**)$_{100}$ (II)$^d$ | 51,260 | 44,220 | 52,320 | 1.18 | 93 | – | – |
| poly(**1b′**)$_{100}$ (I)$^d$ | 45,450 | 41,700 | 50,420 | 1.21 | 97 | – | – |
| poly(**1b′**)$_{100}$ (II)$^d$ | 45,450 | 40,340 | 49,320 | 1.22 | 92 | – | – |
| poly(**1a′**$_{20}$-*ran*-**1c′**$_{80}$) (I) | 51,260 | 30,400 | 33,980 | 1.12 | 94 | 4:1 | 3.9:1 |
| poly(**1a′**$_{20}$-*ran*-**1c′**$_{80}$) (II) | 51,260 | 33,550 | 37,200 | 1.11 | 98 | 4:1 | 3.9:1 |
| poly(**1b′**$_{20}$-*ran*-**1c′**$_{80}$) (I) | 50,100 | 30,260 | 33,900 | 1.12 | 101 | 4:1 | 3.8:1 |
| poly(**1b′**$_{20}$-*ran*-**1c′**$_{80}$) (II) | 50,100 | 31,910 | 35,900 | 1.13 | 98 | 4:1 | 3.9:1 |
| poly(**1a′**)$_5$-*block*-poly(**1c′**)$_{90}$-*block*-poly(**1a′**)$_5$ (I) | 51,260 | 31,810 | 43,320 | 1.36 | 98 | 9:1 | 8.8:1 |
| poly(**1a′**)$_5$-*block*-poly(**1c′**)$_{90}$-*block*-poly(**1a′**)$_5$ (II) | 51,260 | 30,650 | 38,140 | 1.24 | 98 | 9:1 | 7.9:1 |
| poly(**1a′**)$_{10}$-*block*-poly(**1c′**)$_{80}$-*block*-poly(**1a′**)$_{10}$ (I) | 51,260 | 31,520 | 44,290 | 1.40 | 93 | 4:1 | 4.2:1 |
| poly(**1a′**)$_{10}$-*block*-poly(**1c′**)$_{80}$-*block*-poly(**1a′**)$_{10}$ (II) | 51,260 | 34,230 | 47,430 | 1.39 | 98 | 4:1 | 4:1 |
| poly(**1a′**)$_{10}$-*block*-poly(**1c′**)$_{80}$-*block*-poly(**1b′**)$_{10}$ (I) | 50,680 | 41,890 | 47,830 | 1.14 | 104 | 8:1:1 | 8.3:1.1:1 |
| poly(**1a′**)$_{10}$-*block*-poly(**1c′**)$_{80}$-*block*-poly(**1b′**)$_{10}$ (II) | 50,680 | 39,450 | 45,050 | 1.14 | 100 | 8:1:1 | 8:1:1 |
| poly(**1a′**)$_{20}$-*block*-poly(**1c′**)$_{60}$-*block*-poly(**1a′**)$_{20}$ (I) | 51,260 | 35,780 | 48,840 | 1.37 | 91 | 1.5:1 | 1.4:1 |
| poly(**1a′**)$_{20}$-*block*-poly(**1c′**)$_{60}$-*block*-poly(**1a′**)$_{20}$ (II) | 51,260 | 36,690 | 48,780 | 1.33 | 90 | 1.5:1 | 1.6:1 |
| poly(**1b′**)$_5$-*block*-poly(**1c′**)$_{90}$-*block*-poly(**1b′**)$_5$ (I) | 50,680 | 27,500 | 35,530 | 1.29 | 94 | 9:1 | 8.4:1 |
| poly(**1b′**)$_5$-*block*-poly(**1c′**)$_{90}$-*block*-poly(**1b′**)$_5$ (II) | 50,680 | 31,520 | 44,200 | 1.40 | 100 | 9:1 | 7.3:1 |

| | | | | | | | |
|---|---|---|---|---|---|---|---|
| poly(**1b′**)$_{10}$-*block*-poly(**1c′**)$_{80}$-*block*-poly(**1b′**)$_{10}$ (I) | 50,100 | 35,890 | 50,890 | 1.42 | 100 | 4:1 | 3.5:1 |
| poly(**1b′**)$_{10}$-*block*-poly(**1c′**)$_{80}$-*block*-poly(**1b′**)$_{10}$ (II) | 50,100 | 38,150 | 44,490 | 1.17 | 99 | 4:1 | 4.2:1 |
| poly(**1b′**)$_{10}$-*block*-poly(**1c′**)$_{80}$-*block*-poly(**1b′**)$_{10}$ (III) | 50,100 | 37,480 | 43,570 | 1.16 | 103 | 4:1 | 4.4:1 |
| poly(**1b′**)$_{10}$-*block*-poly(**1c′**)$_{80}$-*block*-poly(**1a′**)$_{10}$ (I) | 50,680 | 42,090 | 48,470 | 1.15 | 103 | 8:1:1 | 8:1.3:1 |
| poly(**1b′**)$_{10}$-*block*-poly(**1c′**)$_{80}$-*block*-poly(**1a′**)$_{10}$ (II) | 50,680 | 40,770 | 46,970 | 1.15 | 103 | 8:1:1 | 8.1:1.2:1 |
| poly(**1b′**)$_{20}$-*block*-poly(**1c′**)$_{60}$-*block*-poly(**1b′**)$_{20}$ (I) | 48,940 | 36,460 | 50,240 | 1.38 | 99 | 1.5:1 | 1.7:1 |
| poly(**1b′**)$_{20}$-*block*-poly(**1c′**)$_{60}$-*block*-poly(**1b′**)$_{20}$ (II) | 48,940 | 31,410 | 38,120 | 1.21 | 98 | 1.5:1 | 1.5:1 |

[a]Theoretical molecular weights ($M_n^{theor}$) were calculated based on the catalyst-to-monomer ratio and 100% conversion. [b]Values measured by GPC refractive index (RI) using polystyrene as a standard. [c]Values calculated based on $^{1}$H NMR end-group analysis of the styrene aromatic protons (5H) at δ 7.33 ppm in $CDCl_3$. The protons on the anomeric carbons of the sugars were used to determine the **1c′**:**1a′**/**1b′** ratios. [d]Data for these homopolymers are from a previous study.[13] [e](I), (II), and (III) denote the polymer batch number.

**Table S2.** Comparison of AE Induction in Mouse Sperm by Mannose Glycopolymers

| Polymer Concentration | Polymer Comparison[a] | Statistical Significance (p)[b] |
|---|---|---|
| 0.1 μM | poly(**1a**)$_{100}$ vs. poly(**1a**)$_{10}$-*block*-poly(**1c**)$_{80}$-*block*-poly(**1b**)$_{10}$ | * |
| 1 μM | poly(**1a**)$_{5}$-*block*-poly(**1c**)$_{90}$-*block*-poly(**1a**)$_{5}$ vs. poly(**1a**)$_{20}$-*block*-poly(**1c**)$_{60}$-*block*-poly(**1a**)$_{20}$ | * |
| | poly(**1a**)$_{100}$ vs. poly(**1a**)$_{5}$-*block*-poly(**1c**)$_{90}$-*block*-poly(**1a**)$_{5}$ | * |
| | poly(**1a**)$_{5}$-*block*-poly(**1c**)$_{90}$-*block*-poly(**1a**)$_{5}$ vs. poly(**1a**)$_{10}$-*block*-poly(**1c**)$_{80}$-*block*-poly(**1a**)$_{10}$ | ** |
| | poly(**1a**)$_{5}$-*block*-poly(**1c**)$_{90}$-*block*-poly(**1a**)$_{5}$ vs. poly(**1a**)$_{10}$-*block*-poly(**1c**)$_{80}$-*block*-poly(**1b**)$_{10}$ | *** |
| 5 μM | poly(**1a**)$_{100}$ vs. poly(**1a**)$_{5}$-*block*-poly(**1c**)$_{90}$-*block*-poly(**1a**)$_{5}$ | ** |
| | poly(**1a**)$_{5}$-*block*-poly(**1c**)$_{90}$-*block*-poly(**1a**)$_{5}$ vs. poly(**1a**)$_{10}$-*block*-poly(**1c**)$_{80}$-*block*-poly(**1a**)$_{10}$ | *** |
| | poly(**1a**)$_{5}$-*block*-poly(**1c**)$_{90}$-*block*-poly(**1a**)$_{5}$ vs. poly(**1a**)$_{10}$-*block*-poly(**1c**)$_{80}$-*block*-poly(**1b**)$_{10}$ | ** |
| | poly(**1a**$_{20}$-*ran*-**1c**$_{80}$) vs. poly(**1a**)$_{10}$-*block*-poly(**1c**)$_{80}$-*block*-poly(**1a**)$_{10}$ | * |
| 10 μM | poly(**1a**)$_{100}$ vs. poly(**1a**)$_{20}$-*block*-poly(**1c**)$_{60}$-*block*-poly(**1a**)$_{20}$ | *** |
| | poly(**1a**)$_{100}$ vs. poly(**1a**)$_{5}$-*block*-poly(**1c**)$_{90}$-*block*-poly(**1a**)$_{5}$ | **** |
| | poly(**1a**)$_{100}$ vs. poly(**1a**$_{20}$-*ran*-**1c**$_{80}$) | **** |
| | poly(**1a**)$_{100}$ vs. poly(**1a**)$_{10}$-*block*-poly(**1c**)$_{80}$-*block*-poly(**1b**)$_{10}$ | * |
| | poly(**1a**$_{20}$-*ran*-**1c**$_{80}$) vs. poly(**1a**)$_{10}$-*block*-poly(**1c**)$_{80}$-*block*-poly(**1a**)$_{10}$ | * |

| | | |
| --- | --- | --- |
| | poly(**1a**)$_5$-*block*-poly(**1c**)$_{90}$-*block*-poly(**1a**)$_5$ vs. poly(**1a**)$_{10}$-*block*-poly(**1c**)$_{80}$-*block*-poly(**1a**)$_{10}$ | *** |
| | poly(**1a**)$_5$-*block*-poly(**1c**)$_{90}$-*block*-poly(**1a**)$_5$ vs. poly(**1a**)$_{10}$-*block*-poly(**1c**)$_{80}$-*block*-poly(**1b**)$_{10}$ | ** |
| 20 μM | poly(**1a**)$_{10}$-*block*-poly(**1c**)$_{80}$-*block*-poly(**1a**)$_{10}$ vs. poly(**1a**)$_{20}$-*block*-poly(**1c**)$_{60}$-*block*-poly(**1a**)$_{20}$ | * |
| | poly(**1a**)$_5$-*block*-poly(**1c**)$_{90}$-*block*-poly(**1a**)$_5$ vs. poly(**1a**)$_{10}$-*block*-poly(**1c**)$_{80}$-*block*-poly(**1a**)$_{10}$ | *** |
| | poly(**1a**)$_5$-*block*-poly(**1c**)$_{90}$-*block*-poly(**1a**)$_5$ vs. poly(**1a**)$_{10}$-*block*-poly(**1c**)$_{80}$-*block*-poly(**1b**)$_{10}$ | * |
| | poly(**1a**$_{20}$-*ran*-**1c**$_{80}$) vs. poly(**1a**)$_{10}$-*block*-poly(**1c**)$_{80}$-*block*-poly(**1a**)$_{10}$ | * |

[a]Comparisons of AE activation between polymers are shown and have been adapted from Figure S2A. Polymer comparisons shown are in the same order as illustrated in Figure S2A (bottom to top). Polymer comparisons with no statistical significance have not been included. [b]One-way ANOVA was used to compare AE% where *$p < 0.05$, **$p < 0.01$, ***$p < 0.001$, and ****$p < 0.0001$.

**Table S3.** Comparison of AE Induction in Mouse Sperm by Fucose Glycopolymers

| Polymer Concentration | Polymer Comparison | Statistical Significance (p) |
|---|---|---|
| 0.1 μM | poly(**1b**)$_{100}$ vs. poly(**1b**)$_{10}$-*block*-poly(**1c**)$_{80}$-*block*-poly(**1b**)$_{10}$ | *** |
| | poly(**1b**)$_{100}$ vs. poly(**1b**)$_{10}$-*block*-poly(**1c**)$_{80}$-*block*-poly(**1a**)$_{10}$ | ** |
| | poly(**1b**)$_{5}$-*block*-poly(**1c**)$_{90}$-*block*-poly(**1b**)$_{5}$ vs. poly(**1b**)$_{10}$-*block*-poly(**1c**)$_{80}$-*block*-poly(**1b**)$_{10}$ | ** |
| | poly(**1b**$_{20}$-*ran*-**1c**$_{80}$) vs. poly(**1b**)$_{10}$-*block*-poly(**1c**)$_{80}$-*block*-poly(**1b**)$_{10}$ | *** |
| | poly(**1b**$_{20}$-*ran*-**1c**$_{80}$) vs. poly(**1b**)$_{10}$-*block*-poly(**1c**)$_{80}$-*block*-poly(**1a**)$_{10}$ | ** |
| 1 μM | poly(**1b**)$_{5}$-*block*-poly(**1c**)$_{90}$-*block*-poly(**1b**)$_{5}$ vs. poly(**1b**)$_{10}$-*block*-poly(**1c**)$_{80}$-*block*-poly(**1b**)$_{10}$ | * |
| | poly(**1b**$_{20}$-*ran*-**1c**$_{80}$) vs. poly(**1b**)$_{10}$-*block*-poly(**1c**)$_{80}$-*block*-poly(**1b**)$_{10}$ | * |
| 5 μM | poly(**1b**)$_{5}$-*block*-poly(**1c**)$_{90}$-*block*-poly(**1b**)$_{5}$ vs. poly(**1b**)$_{10}$-*block*-poly(**1c**)$_{80}$-*block*-poly(**1b**)$_{10}$ | ** |
| | poly(**1b**$_{20}$-*ran*-**1c**$_{80}$) vs. poly(**1b**)$_{10}$-*block*-poly(**1c**)$_{80}$-*block*-poly(**1b**)$_{10}$ | ** |
| | poly(**1b**)$_{5}$-*block*-poly(**1c**)$_{90}$-*block*-poly(**1b**)$_{5}$ vs. poly(**1b**)$_{10}$-*block*-poly(**1c**)$_{80}$-*block*-poly(**1a**)$_{10}$ | *** |
| | poly(**1b**$_{20}$-*ran*-**1c**$_{80}$) vs. poly(**1b**)$_{10}$-*block*-poly(**1c**)$_{80}$-*block*-poly(**1a**)$_{10}$ | ** |
| 10 μM | poly(**1b**)$_{100}$ vs. poly(**1b**)$_{20}$-*block*-poly(**1c**)$_{60}$-*block*-poly(**1b**)$_{20}$ | ** |
| | poly(**1b**)$_{100}$ vs. poly(**1b**)$_{5}$-*block*-poly(**1c**)$_{90}$-*block*-poly(**1b**)$_{5}$ | **** |
| | poly(**1b**)$_{100}$ vs. poly(**1b**$_{20}$-*ran*-**1c**$_{80}$) | **** |

| | | |
|---|---|---|
| | poly(**1b**)$_{10}$-*block*-poly(**1c**)$_{80}$-*block*-poly(**1a**)$_{10}$ vs. poly(**1b**)$_{20}$-*block*-poly(**1c**)$_{60}$-*block*-poly(**1b**)$_{20}$ | * |
| | poly(**1b**$_{20}$-*ran*-**1c**$_{80}$) vs. poly(**1b**)$_{10}$-*block*-poly(**1c**)$_{80}$-*block*-poly(**1b**)$_{10}$ | ** |
| | poly(**1b**)$_{5}$-*block*-poly(**1c**)$_{90}$-*block*-poly(**1b**)$_{5}$ vs. poly(**1b**)$_{10}$-*block*-poly(**1c**)$_{80}$-*block*-poly(**1b**)$_{10}$ | ** |
| | poly(**1b**)$_{5}$-*block*-poly(**1c**)$_{90}$-*block*-poly(**1b**)$_{5}$ vs. poly(**1b**)$_{10}$-*block*-poly(**1c**)$_{80}$-*block*-poly(**1a**)$_{10}$ | *** |
| | poly(**1b**$_{20}$-*ran*-**1c**$_{80}$) vs. poly(**1b**)$_{10}$-*block*-poly(**1c**)$_{80}$-*block*-poly(**1a**)$_{10}$ | *** |
| 20 μM | poly(**1b**$_{20}$-*ran*-**1c**$_{80}$) vs. poly(**1b**)$_{10}$-*block*-poly(**1c**)$_{80}$-*block*-poly(**1b**)$_{10}$ | * |
| | poly(**1b**$_{20}$-*ran*-**1c**$_{80}$) vs. poly(**1b**)$_{10}$-*block*-poly(**1c**)$_{80}$-*block*-poly(**1a**)$_{10}$ | * |

[a]Comparisons of AE activation between polymers are shown and have been adapted from Figure S2B. Polymer comparisons shown are in the same order as illustrated in Figure S2B (bottom to top). Polymer comparisons with no statistical significance have not been included. [b]One-way ANOVA was used to compare AE% where *$p < 0.05$, **$p < 0.01$, ***$p < 0.001$, and ****$p < 0.0001$.

**Table S4.** Slopes at the High-*q* Region from SAXS Data Fit at 1% (w/v) of Norbornene Homopolymers, Random Copolymers, and Block Copolymers

| Polymer | Slope at High-$q^a$ |
|---|---|
| poly(**1a**)$_{100}$ (I)$^{b,\ c}$ | 3.0 |
| poly(**1b**)$_{100}$ (II)$^{b}$ | 2.9 |
| poly(**1a′**$_{20}$-*ran*-**1c′**$_{80}$) (II) | 2.9 |
| poly(**1b′**$_{20}$-*ran*-**1c′**$_{80}$) (II) | 2.8 |
| poly(**1a′**)$_{5}$-*block*-poly(**1c′**)$_{90}$-*block*-poly(**1a′**)$_{5}$ (I) | 2.7 |
| poly(**1a′**)$_{10}$-*block*-poly(**1c′**)$_{80}$-*block*-poly(**1a′**)$_{10}$ (I) | 2.7 |
| poly(**1a′**)$_{10}$-*block*-poly(**1c′**)$_{80}$-*block*-poly(**1b′**)$_{10}$ (I) | 2.7 |
| poly(**1a′**)$_{20}$-*block*-poly(**1c′**)$_{60}$-*block*-poly(**1a′**)$_{20}$ (I) | 2.7 |
| poly(**1b′**)$_{5}$-*block*-poly(**1c′**)$_{90}$-*block*-poly(**1b′**)$_{5}$ (I) | 2.8 |
| poly(**1b′**)$_{10}$-*block*-poly(**1c′**)$_{80}$-*block*-poly(**1b′**)$_{10}$ (III) | 2.7 |
| poly(**1b′**)$_{10}$-*block*-poly(**1c′**)$_{80}$-*block*-poly(**1a′**)$_{10}$ (I) | 2.7 |
| poly(**1b′**)$_{20}$-*block*-poly(**1c′**)$_{60}$-*block*-poly(**1b′**)$_{20}$ (I) | 2.7 |

[a]Values for this parameter were determined by the slope of the data between the *q* region of 0.1 and 0.2 $Å^{-1}$. When the data is fit with a flexible cylinder model, the high-*q* region can provide data about the cross-section or radius of the structure in solution. [b]Data for these homopolymers are from a previous study. [c](I), (II), and (III) denote the polymer batch number.

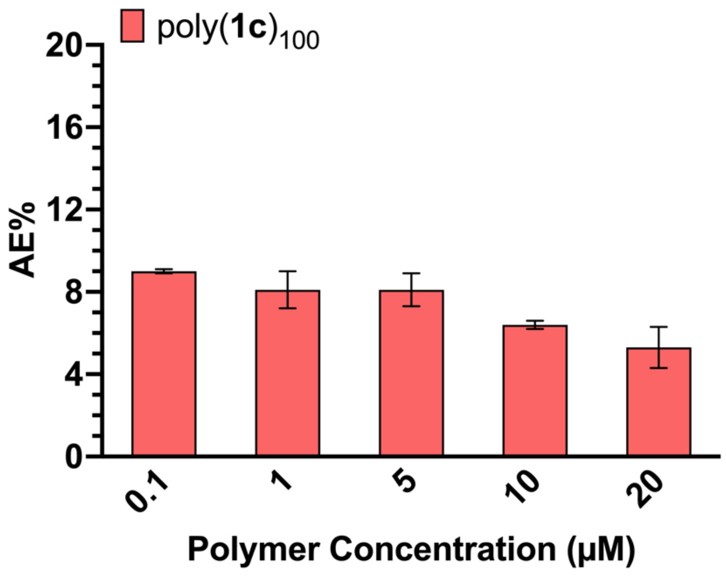


**Figure S1.** AE activation in mouse sperm by poly(**1c**)$_{100}$. The average AE% for mouse sperm treated with Dulbecco's phosphate-buffered saline, DPBS (negative control), was 7.2%. Data represents mean ± standard error of the mean of at least three independent experiments testing two batches of polymer. One-way ANOVA was used to compare AE% to the negative control. There was no statistically significant difference between the AE% of sperm treated with the negative control and sperm treated with poly(**1c**)$_{100}$.

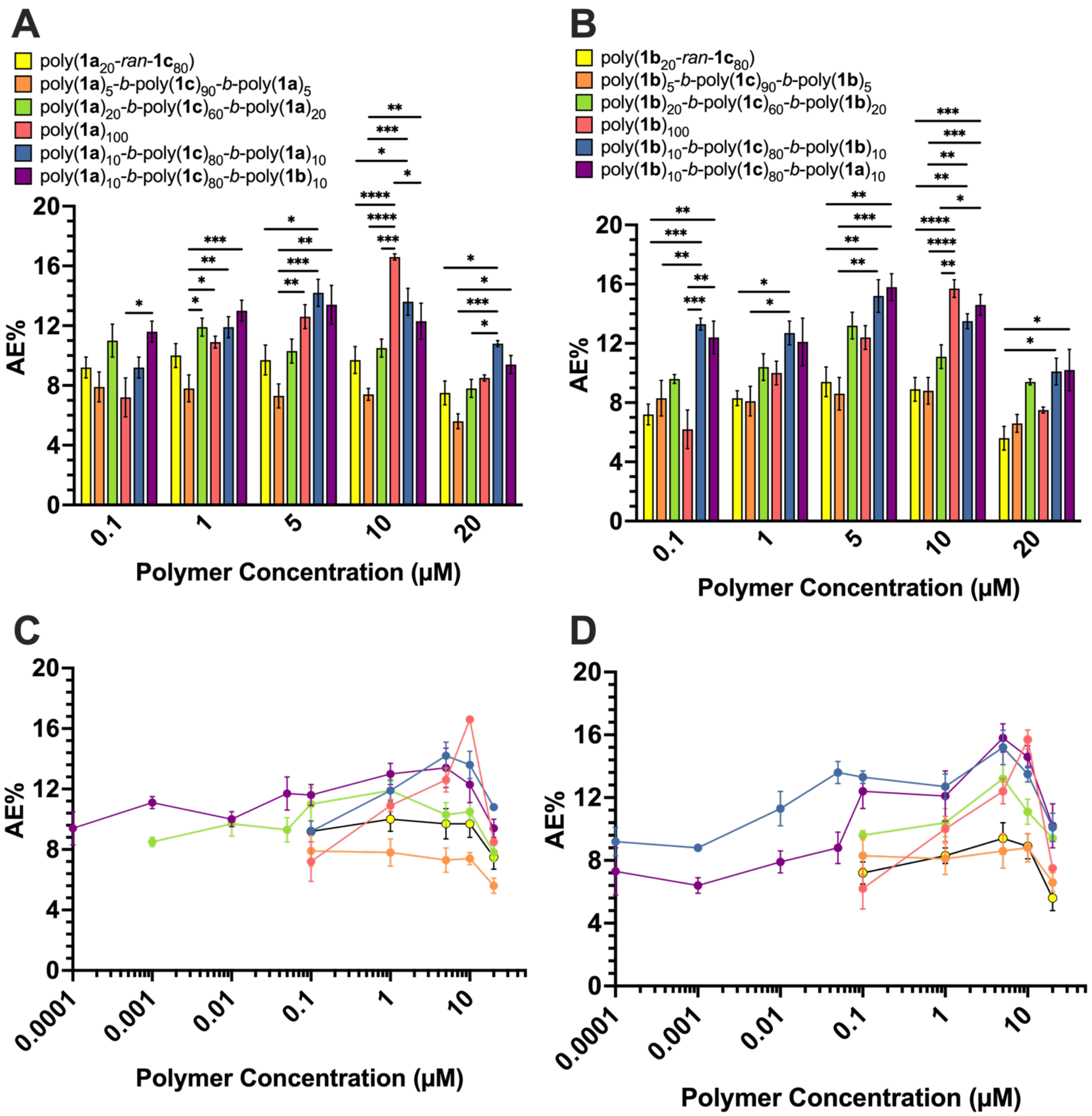


**Figure S2.** Comparison of AE induction in mouse sperm by (**A**) mannose or (**B**) fucose glycopolymers. AE activation curves of (**C**) mannose and (**D**) fucose glycopolymers are shown on a $\log_{10}$ scale. The average AE% for mouse sperm treated with DPBS (negative control) was 7.2%. Data represents mean ± standard error of the mean of at least three independent experiments testing at least two batches of each polymer. One-way ANOVA was used to compare AE% where *$p < 0.05$, **$p < 0.01$, ***$p < 0.001$, and ****$p < 0.0001$.

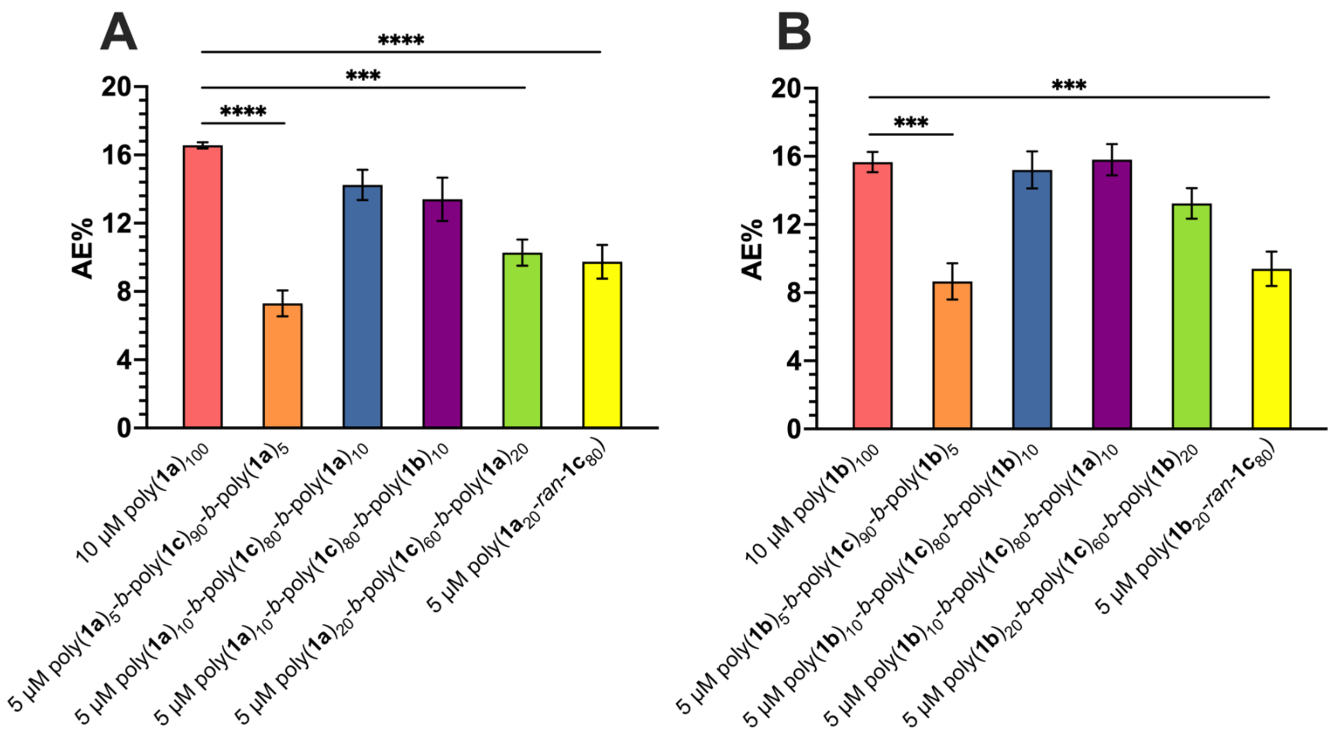


**Figure S3.** AE% of (**A**) mannose and (**B**) fucose glycopolymers at 5 μM polymer concentration compared to the maximum AE% of poly(**1a**)$_{100}$ and poly(**1b**)$_{100}$, respectively. The average AE% for mouse sperm treated with DPBS (negative control) was 7.2%. Data represents mean ± standard error of the mean of at least three independent experiments testing at least two batches of each polymer. One-way ANOVA was used to compare AE% where *$p < 0.05$, **$p < 0.01$, ***$p < 0.001$, and ****$p < 0.0001$.

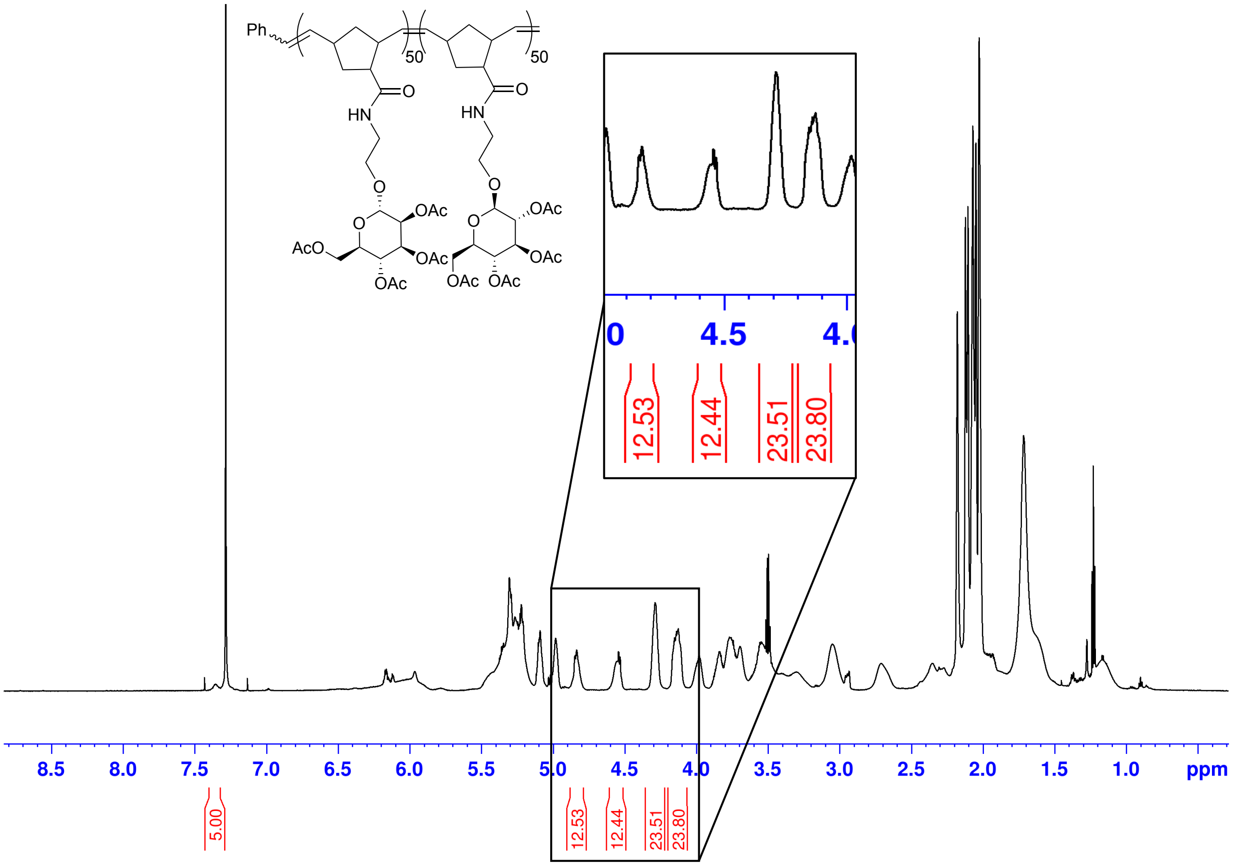


**Figure S4.** $^{1}$H NMR (400 MHz, $CDCl_3$) spectrum of **1c′** and **1a′** (1:1) incorporated into random copolymers. The polymerization was run to approximately 20–30% conversion. $^{1}$H NMR end-group analysis of the styrene aromatic protons (5H) at δ 7.33 ppm was used to determine the proton integration of the anomeric carbon for each monomer to determine the monomer ratios. The **1c′**:**1a′** ratio was 1:1.

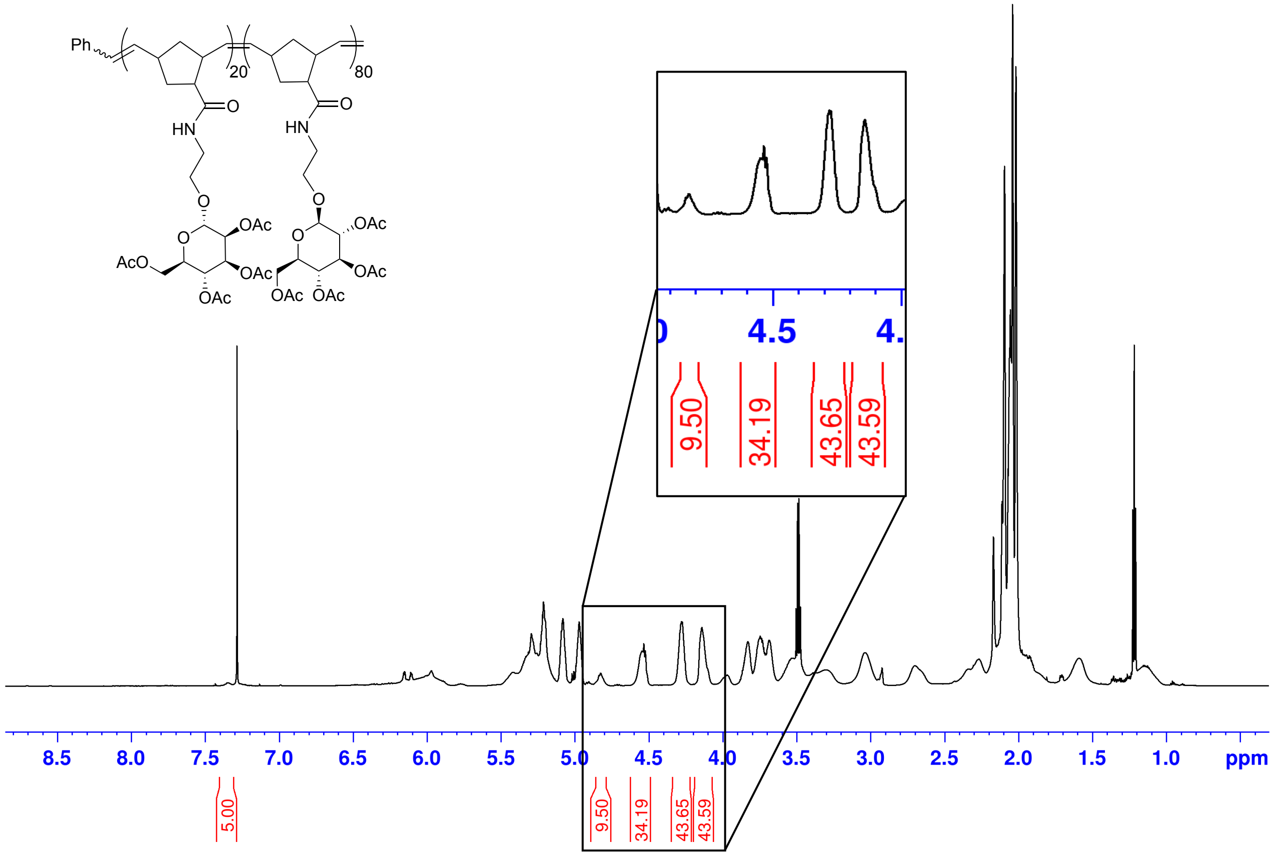


**Figure S5.** $^{1}$H NMR (400 MHz, $CDCl_3$) spectrum of **1c′** and **1a′** (4:1) incorporated into random copolymers. The polymerization was run to approximately 20–30% conversion. $^{1}$H NMR end-group analysis of the styrene aromatic protons (5H) at δ 7.33 ppm was used to determine the proton integration of the anomeric carbon for each monomer to determine the monomer ratios. The **1c′**:**1a′** ratio was 3.4:1.

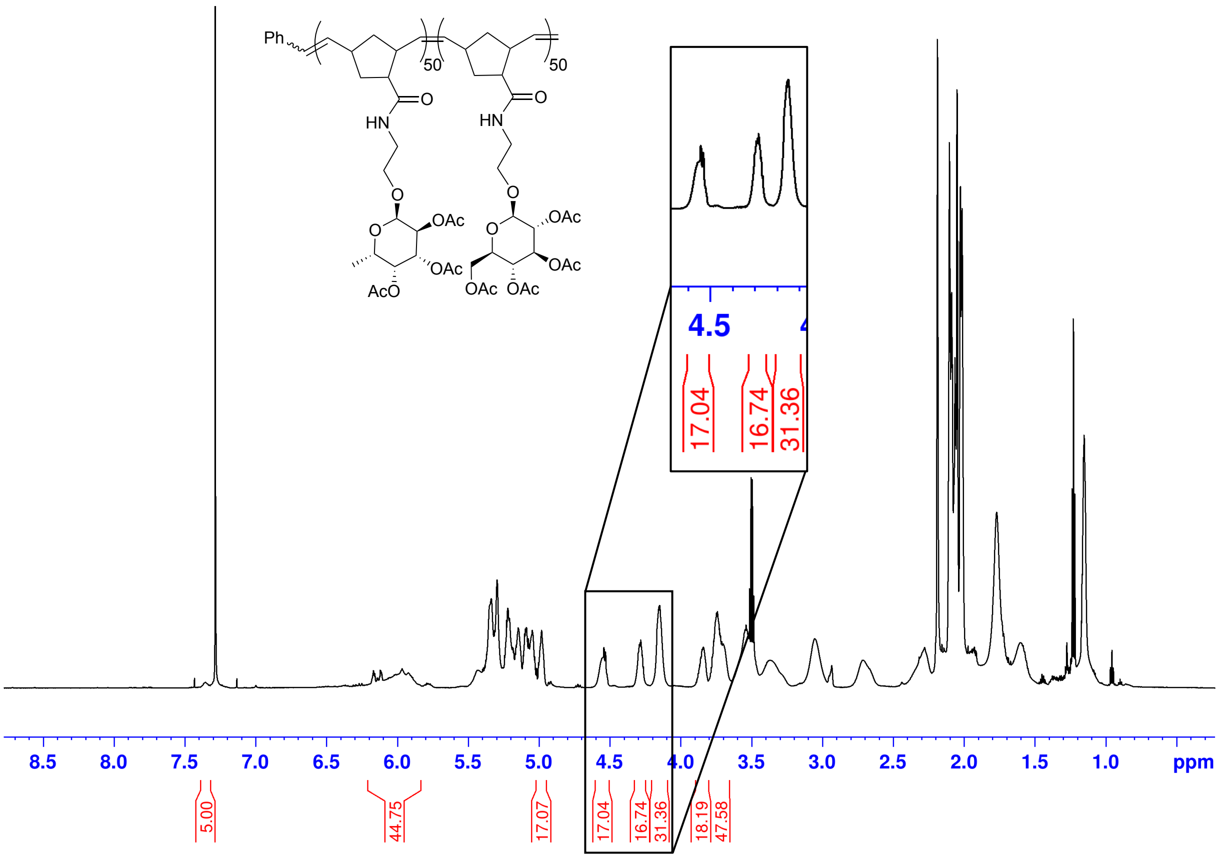


**Figure S6.** $^{1}$H NMR (400 MHz, $CDCl_3$) spectrum of **1c′** and **1b′** (1:1) incorporated into random copolymers. The polymerization was run to approximately 20–30% conversion. $^{1}$H NMR end-group analysis of the styrene aromatic protons (5H) at δ 7.33 ppm was used to determine the proton integration of the anomeric carbon for each monomer to determine the monomer ratios. The **1c′**:**1b′** ratio was 1.2:1.

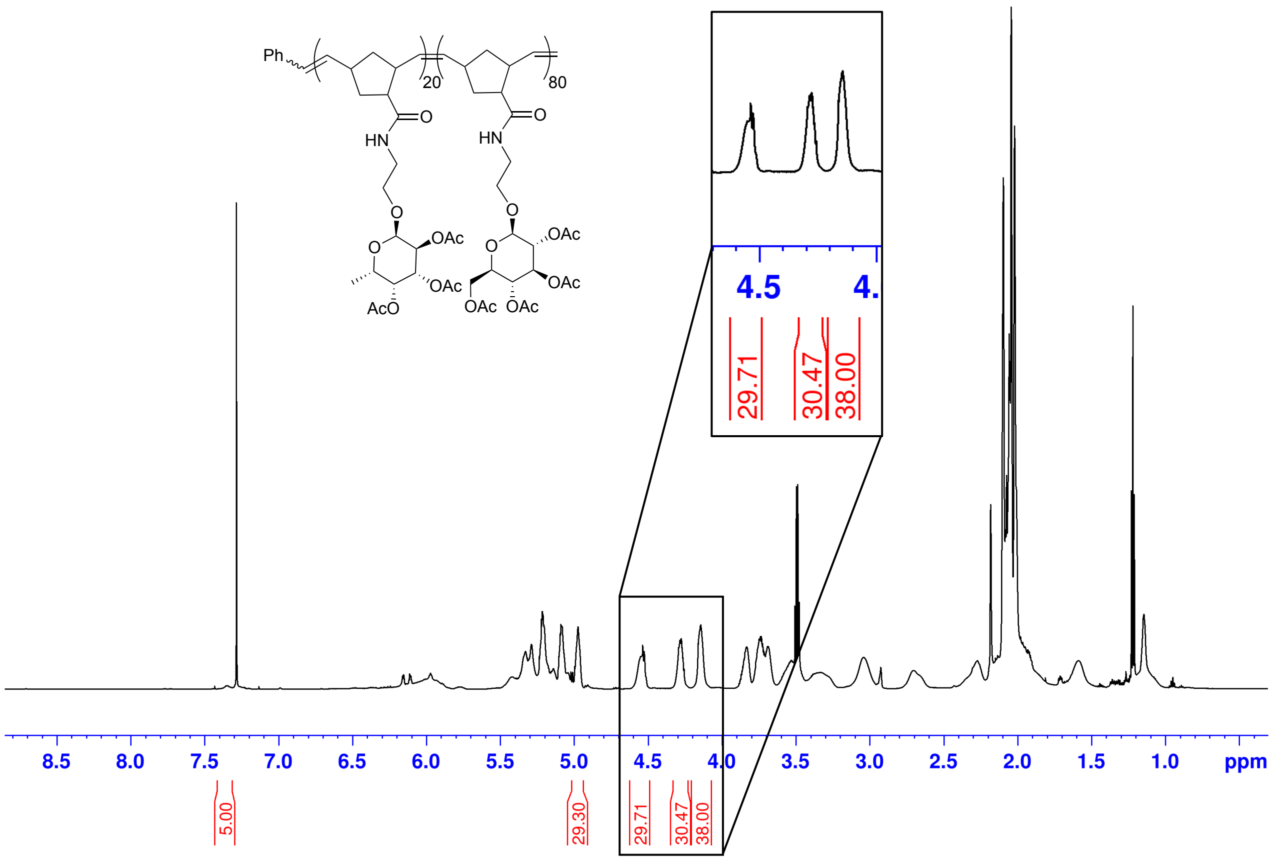


**Figure S7.** $^{1}$H NMR (400 MHz, $CDCl_3$) spectrum of **1c′** and **1b′** (4:1) incorporated into random copolymers. The polymerization was run to approximately 20–30% conversion. $^{1}$H NMR end-group analysis of the styrene aromatic protons (5H) at δ 7.33 ppm was used to determine the proton integration of the anomeric carbon for each monomer to determine the monomer ratios. The **1c′**:**1b′** ratio was 3.8:1.

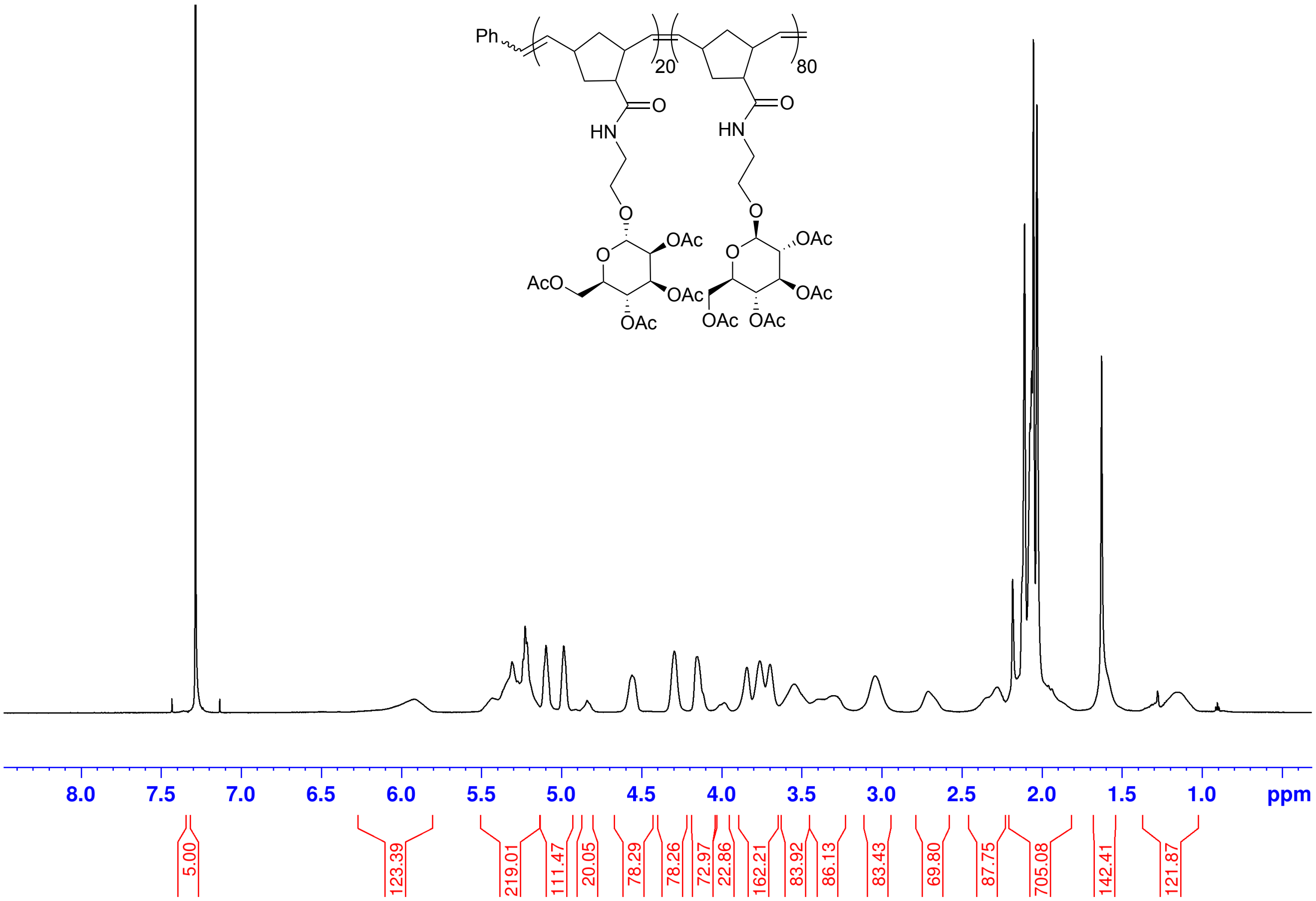


**Figure S8**. $^{1}$H NMR (700 MHz, $CDCl_3$) spectrum of poly(**1a′**$_{20}$-*ran*-**1c′**$_{80}$)

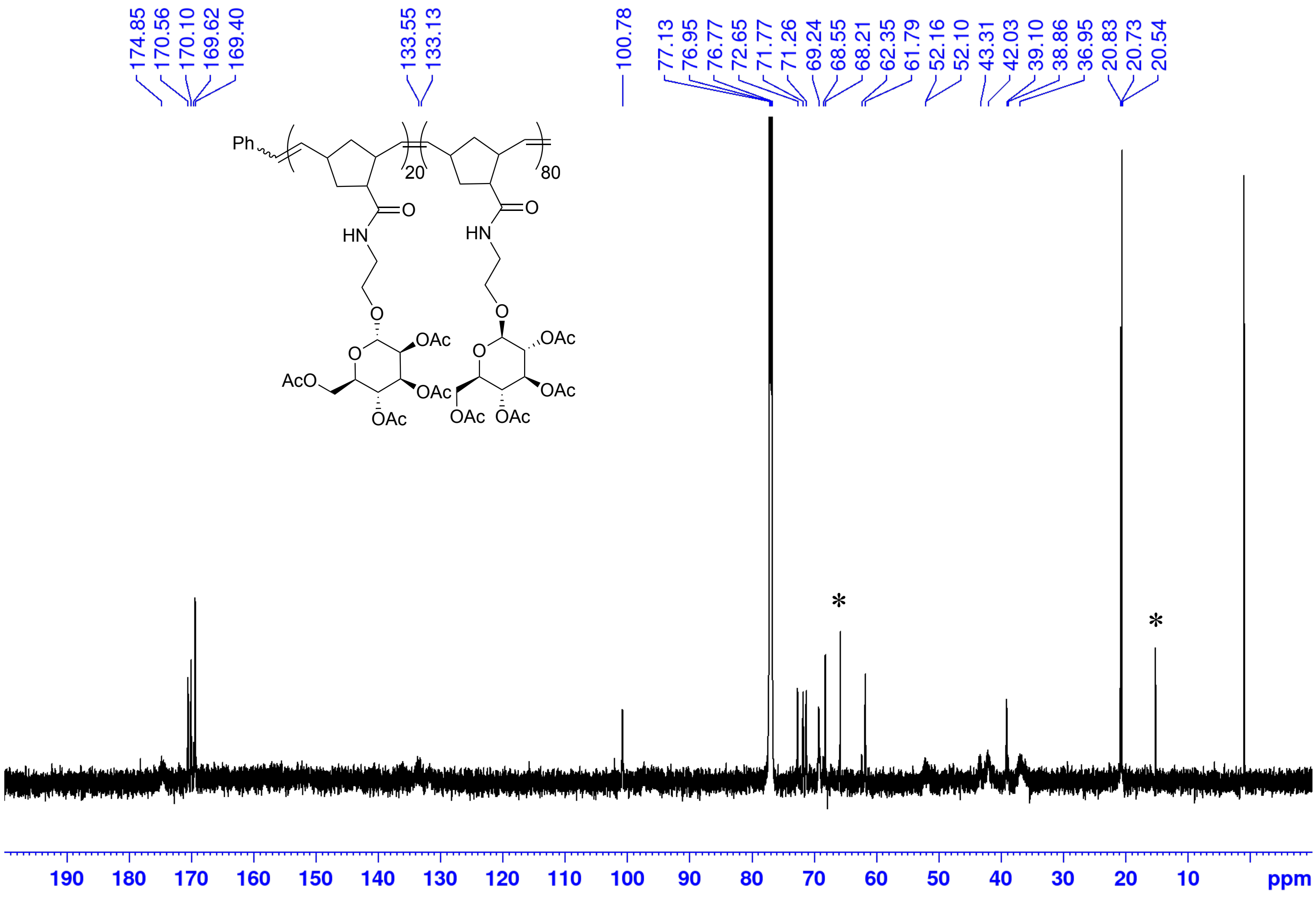


**Figure S9**. $^{13}$C NMR (176 MHz, $CDCl_3$) spectrum of poly(**1a′**$_{20}$-*ran*-**1c′**$_{80}$)

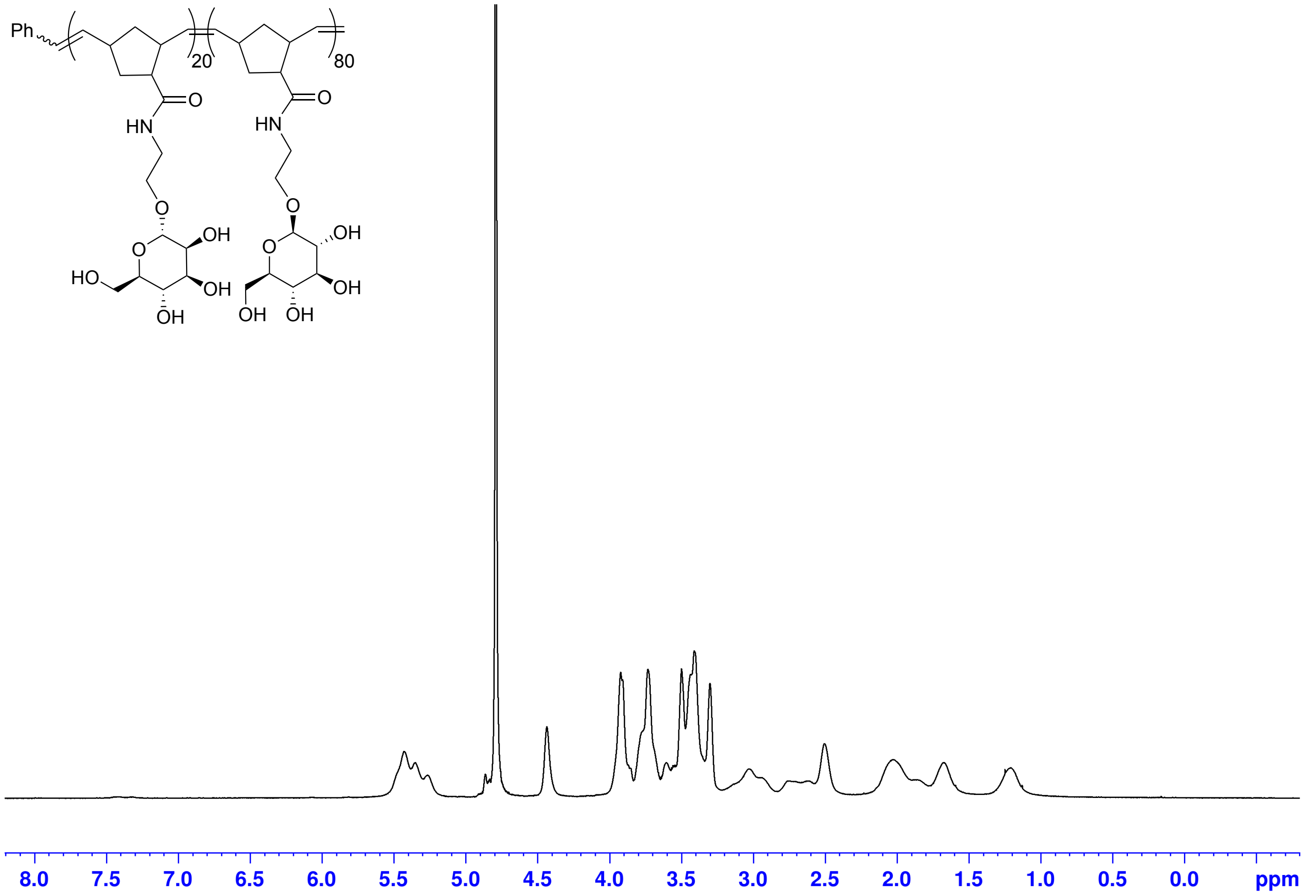


**Figure S10**. $^{1}$H NMR (700 MHz, $D_2O$) spectrum of poly(**1a**$_{20}$-*ran*-**1c**$_{80}$)

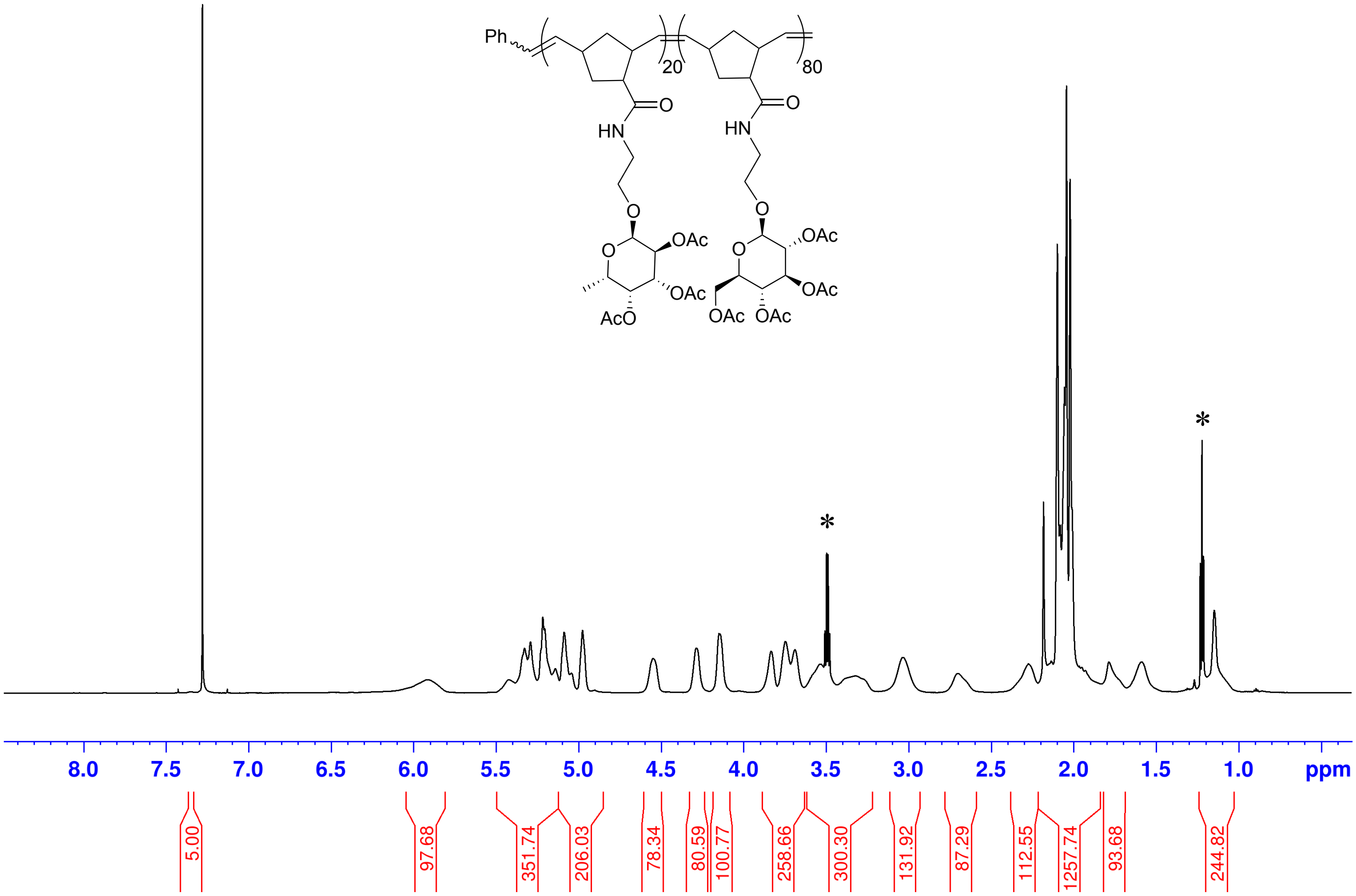


**Figure S11**. $^{1}$H NMR (700 MHz, $CDCl_3$) spectrum of poly(**1b′**$_{20}$-*ran*-**1c′**$_{80}$)

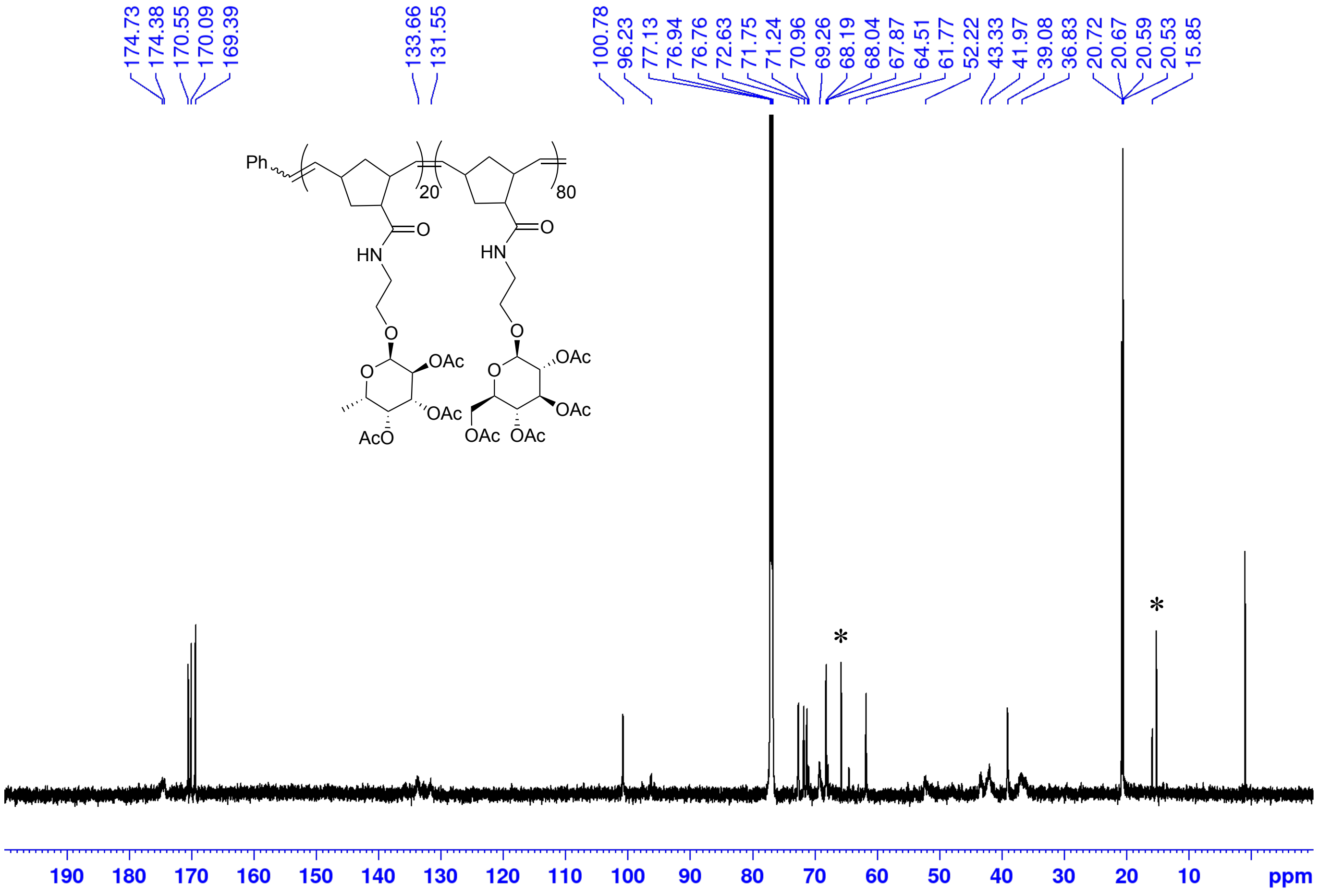


**Figure S12**. $^{13}$C NMR (176 MHz, $CDCl_3$) spectrum of poly(**1b′**$_{20}$-*ran*-**1c′**$_{80}$)

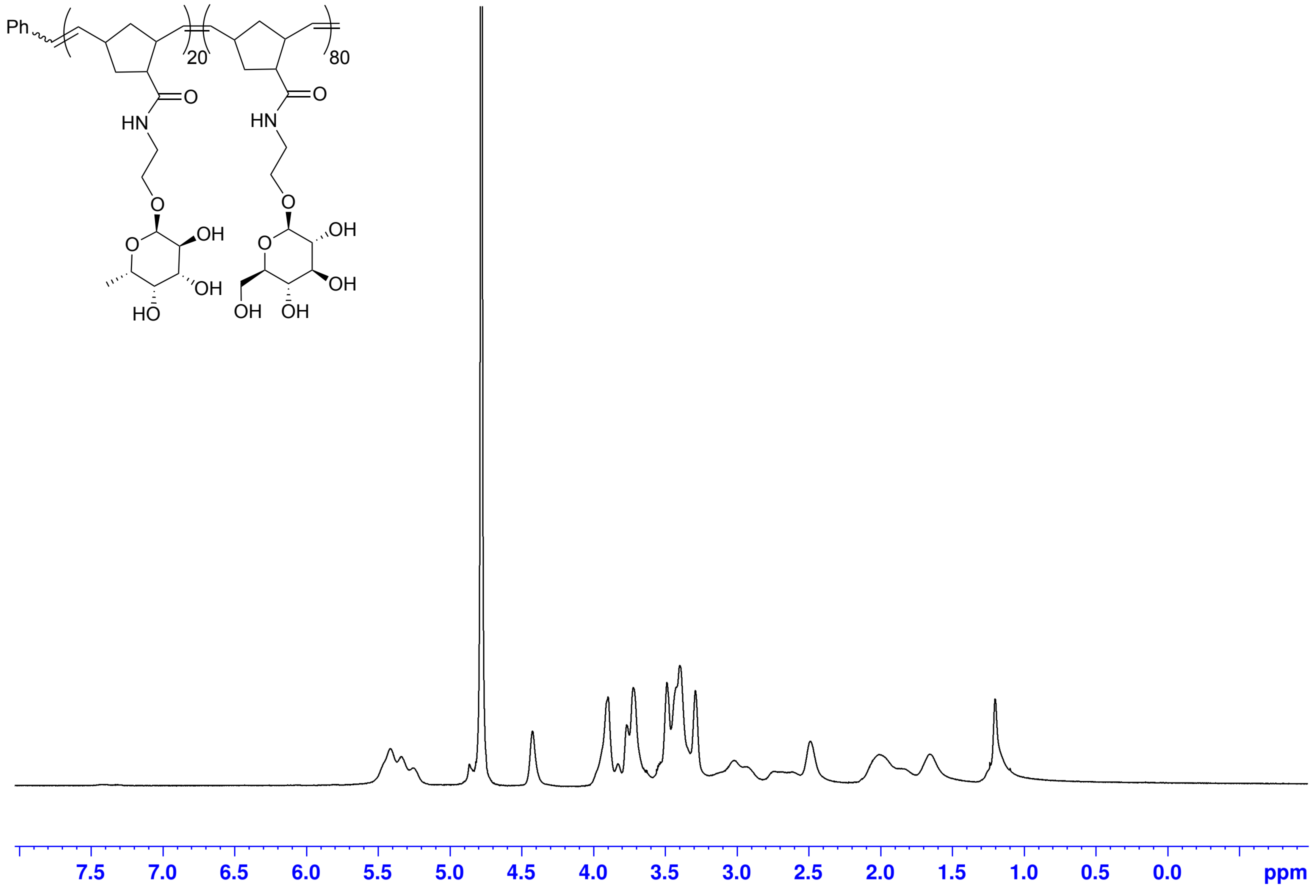


**Figure S13**. $^{1}$H NMR (700 MHz, $D_2O$) spectrum of poly(**1b**$_{20}$-*ran*-**1c**$_{80}$)

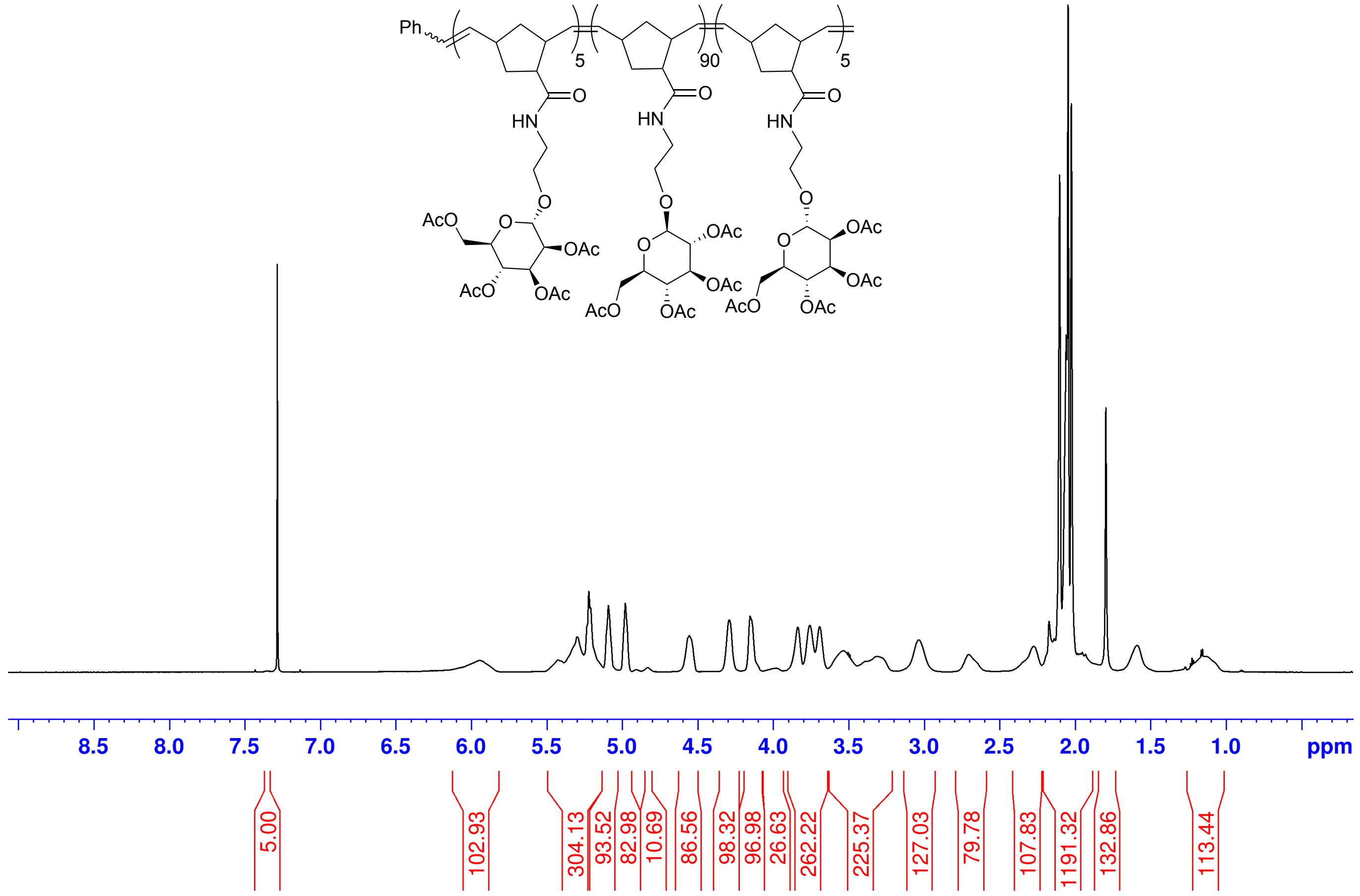


**Figure S14**. $^{1}$H NMR (700 MHz, $CDCl_3$) spectrum of poly(**1a′**)$_5$-*block*-poly(**1c′**)$_{90}$-*block*-poly(**1a′**)$_5$

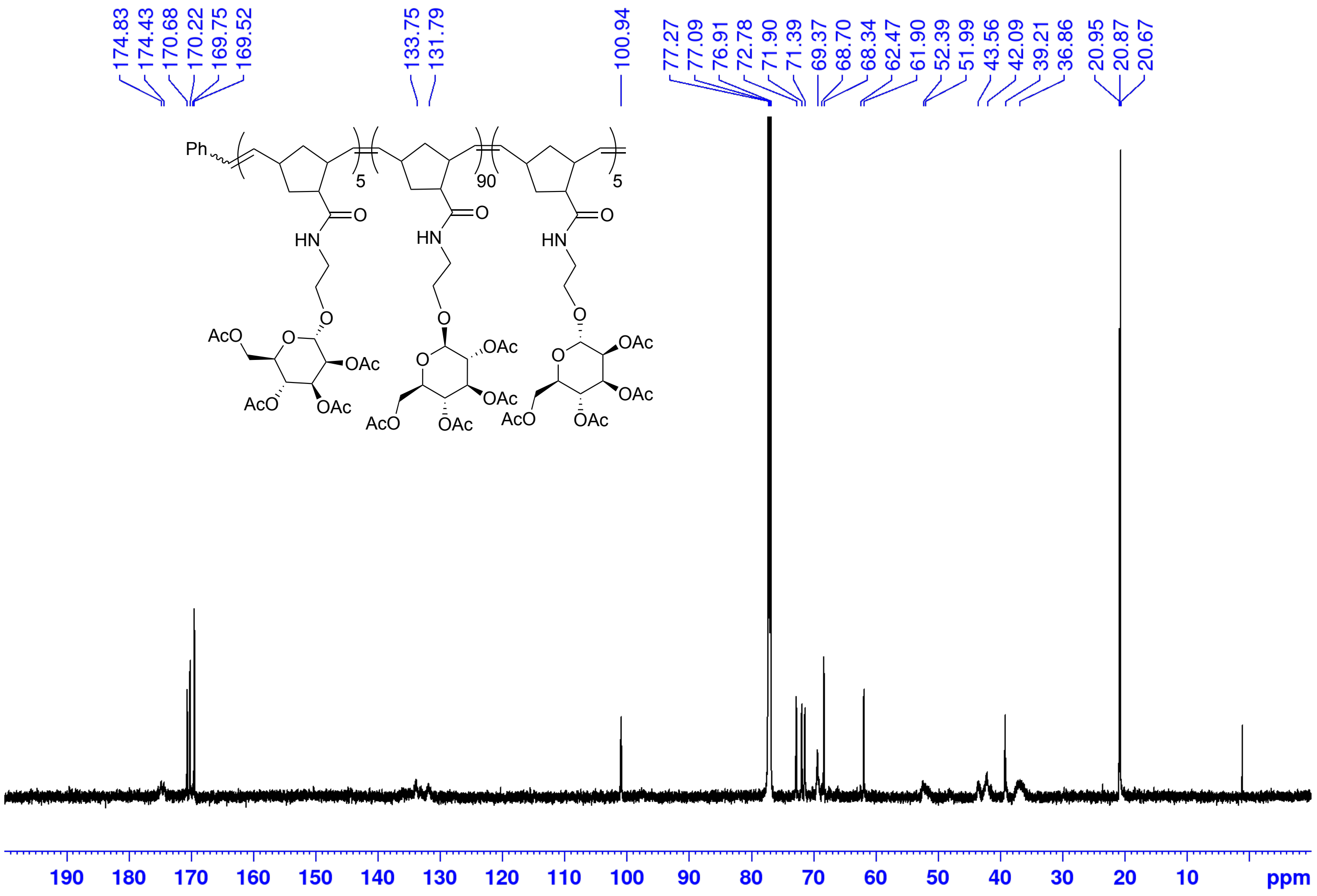


**Figure S15**. $^{13}$C NMR (176 MHz, $CDCl_3$) spectrum of poly(**1a′**)$_5$-*block*-poly(**1c′**)$_{90}$-*block*-poly(**1a′**)$_5$

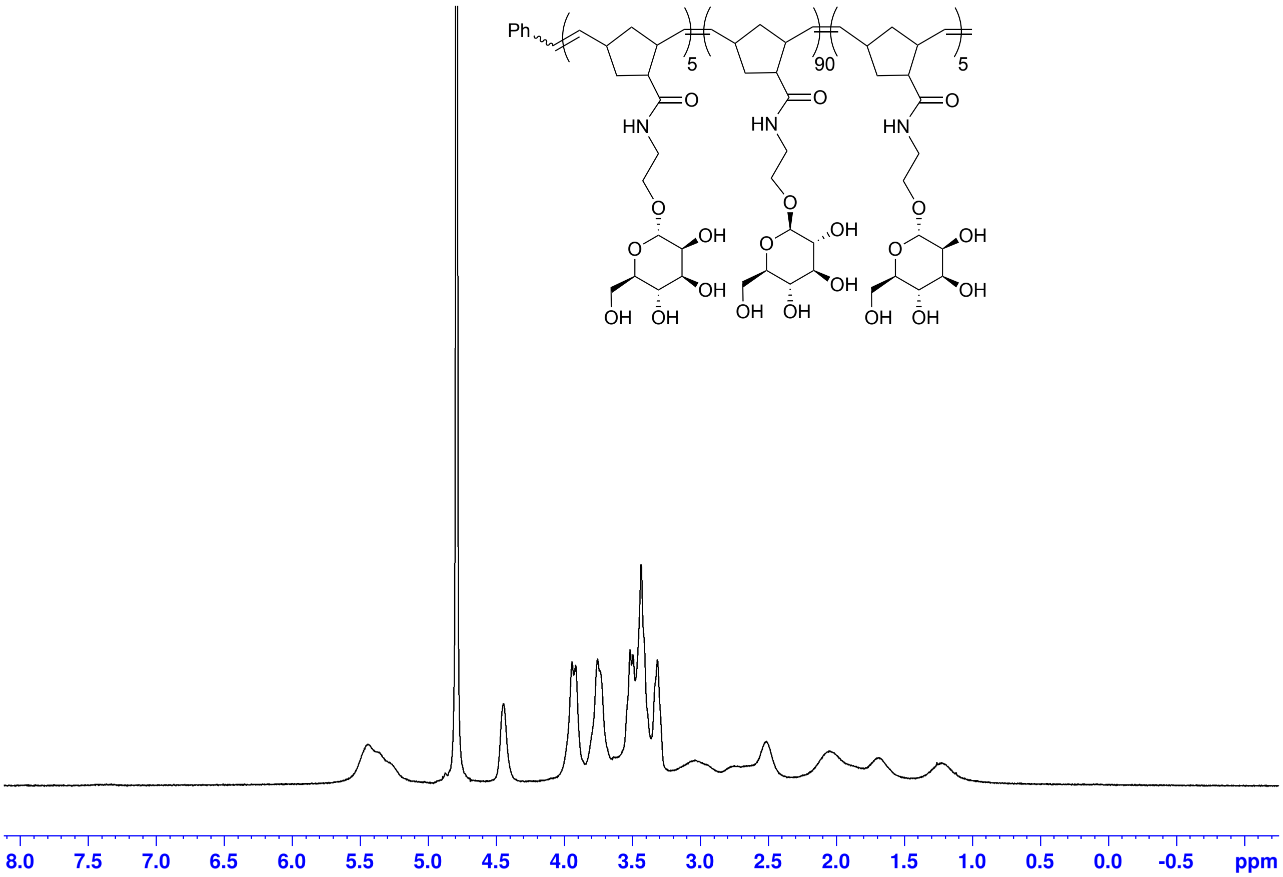


**Figure S16**. $^{1}$H NMR (400 MHz, $D_2O$) spectrum of poly(**1a**)$_5$-*block*-poly(**1c**)$_{90}$-*block*-poly(**1a**)$_5$

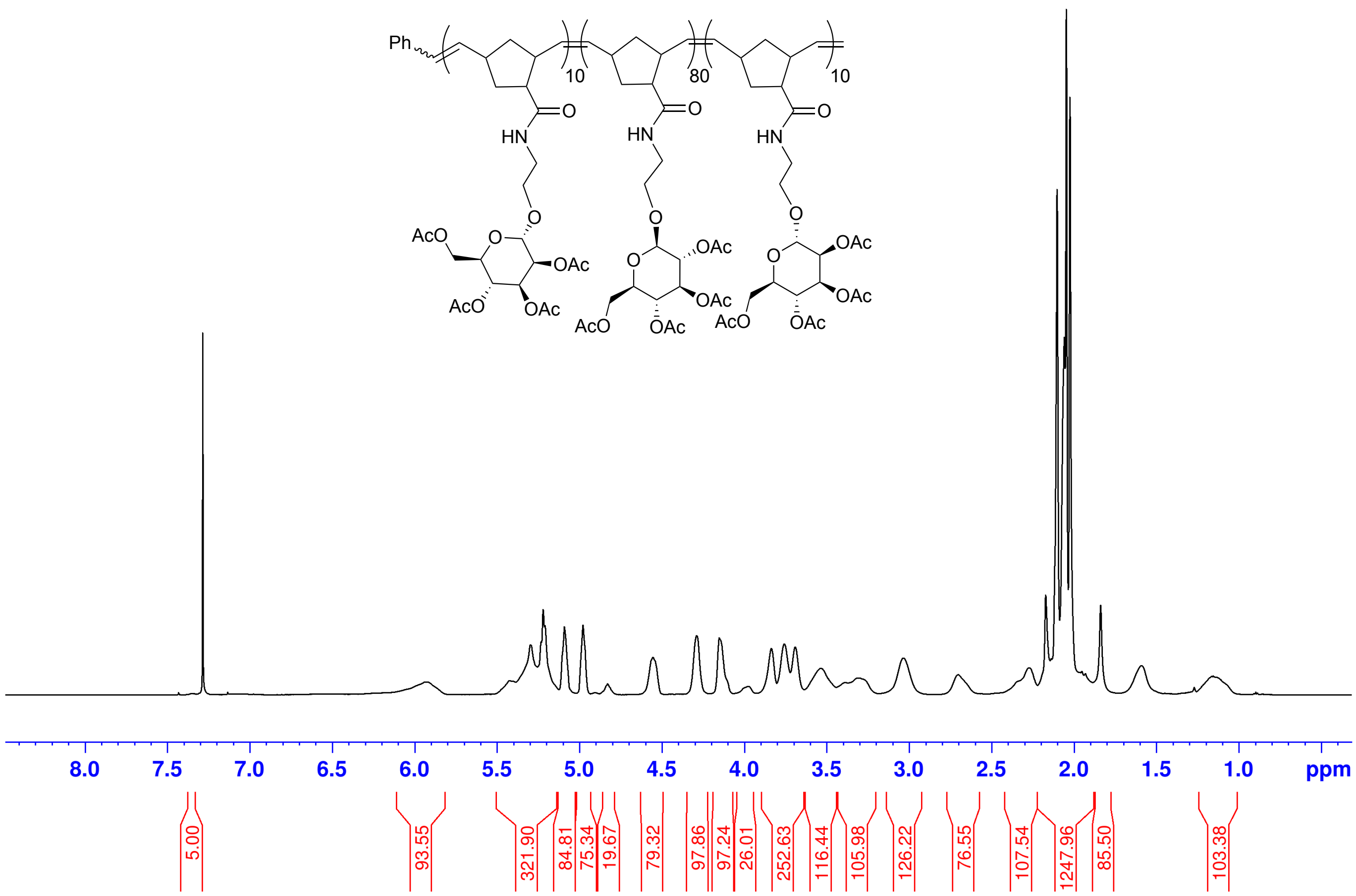


**Figure S17**. $^{1}$H NMR (700 MHz, $CDCl_3$) spectrum of poly(**1a′**)$_{10}$-*block*-poly(**1c′**)$_{80}$-*block*-poly(**1a′**)$_{10}$

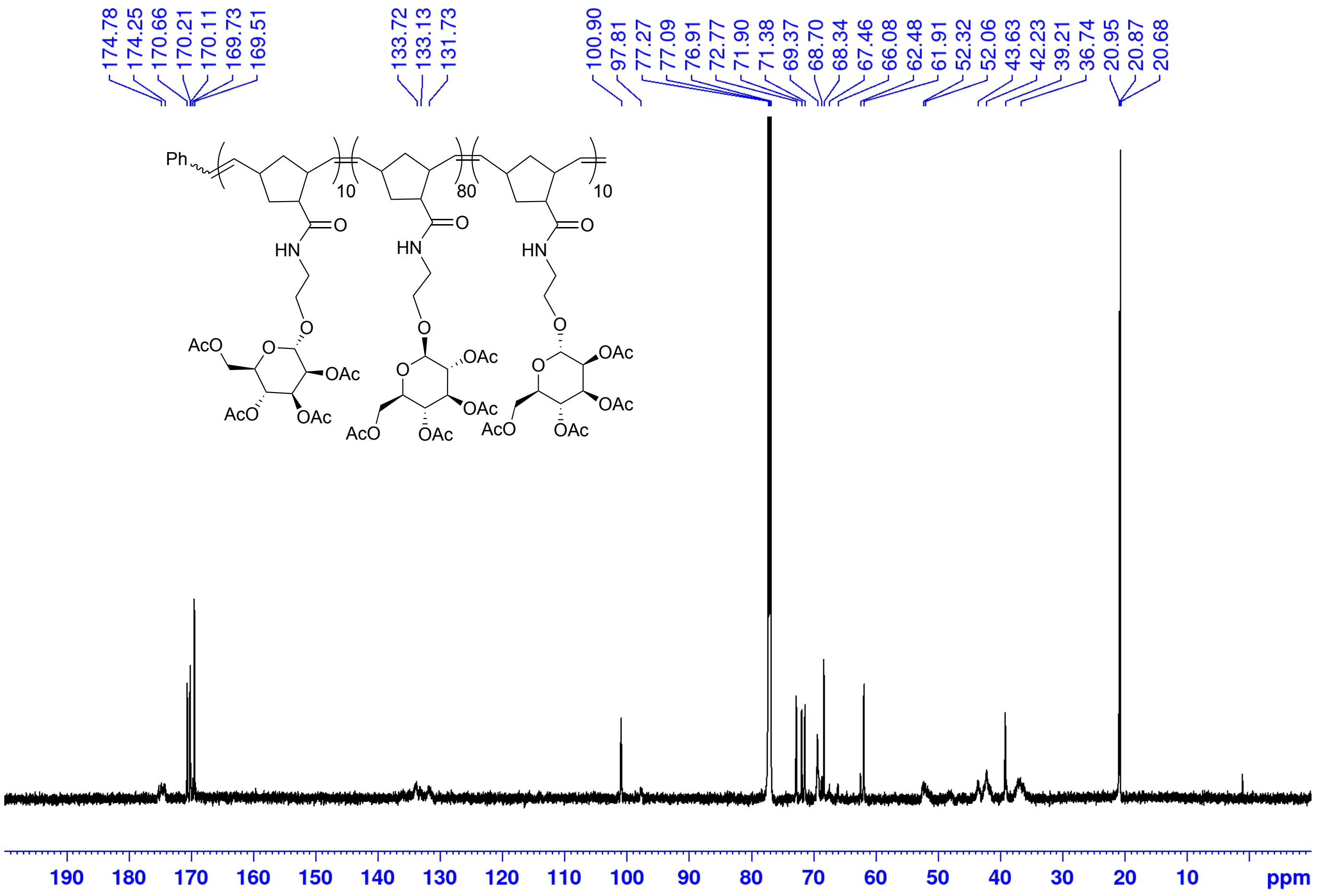


**Figure S18**. $^{13}$C NMR (176 MHz, $CDCl_3$) spectrum of poly(**1a′**)$_{10}$-*block*-poly(**1c′**)$_{80}$-*block*-poly(**1a′**)$_{10}$

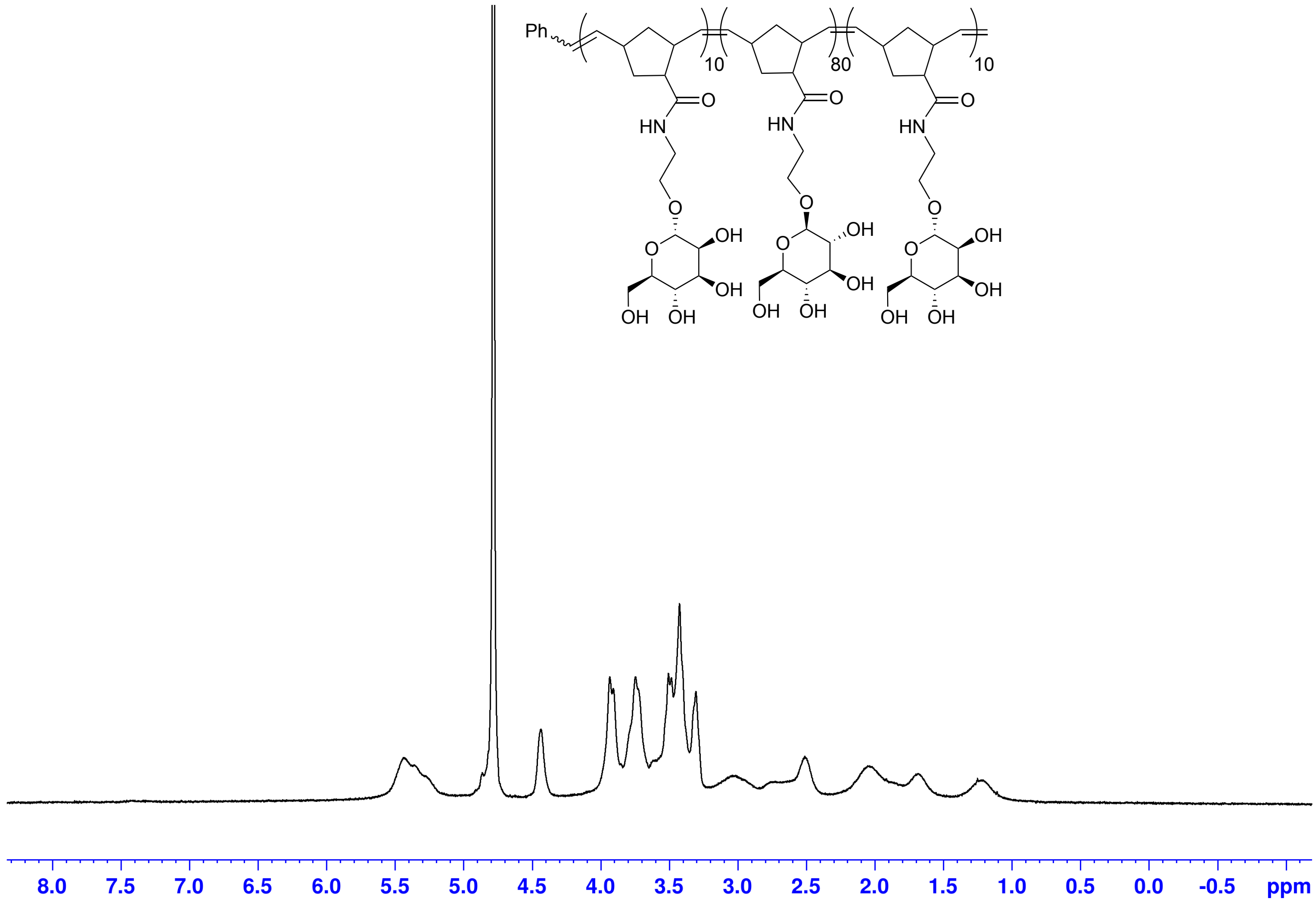


**Figure S19**. $^{1}$H NMR (400 MHz, $D_2O$) spectrum of poly(**1a**)$_{10}$-*block*-poly(**1c**)$_{80}$-*block*-poly(**1a**)$_{10}$

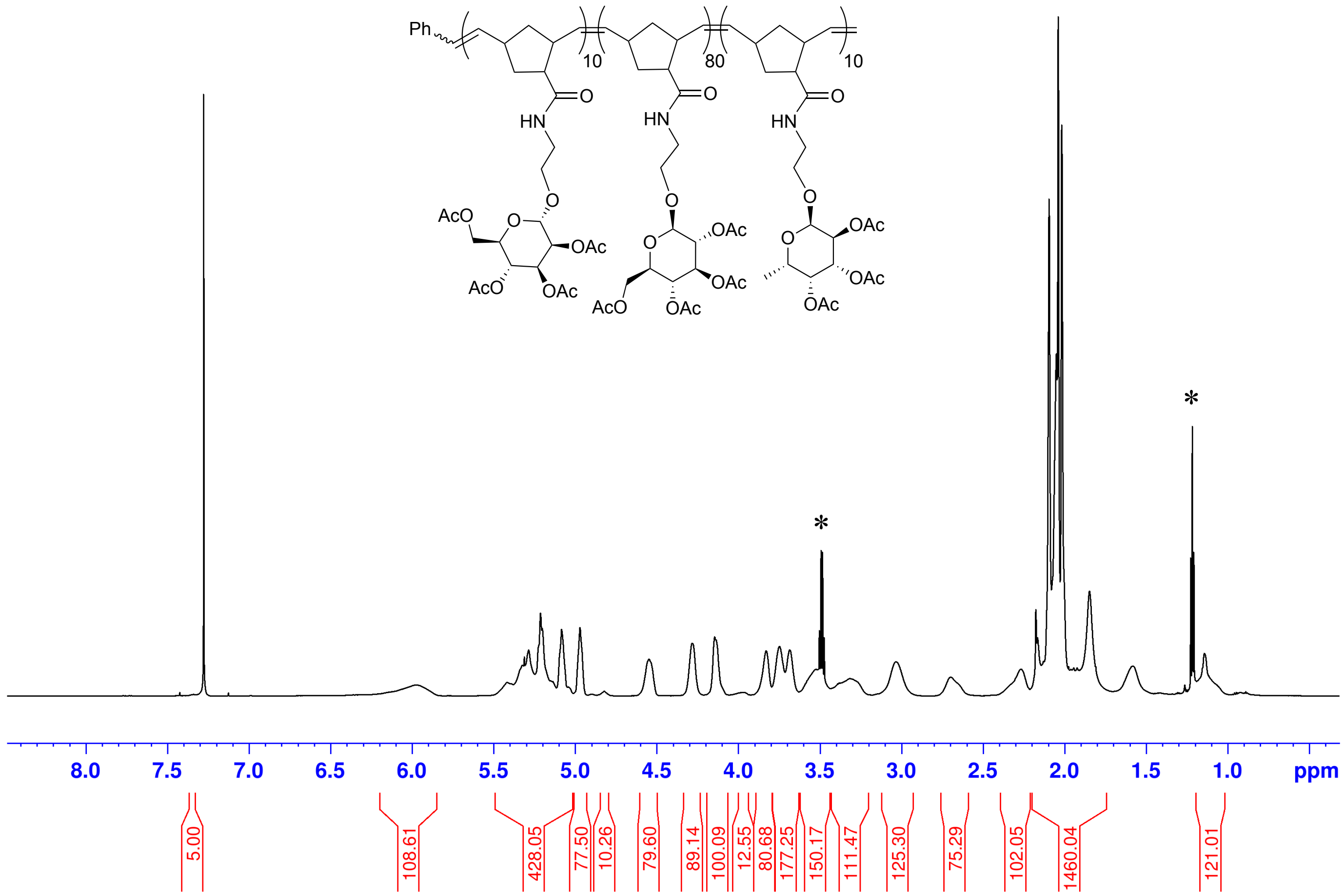

**Figure S20**. $^{1}$H NMR (700 MHz, $CDCl_3$) spectrum of poly(**1a′**)$_{10}$-*block*-poly(**1c′**)$_{80}$-*block*-poly(**1b′**)$_{10}$

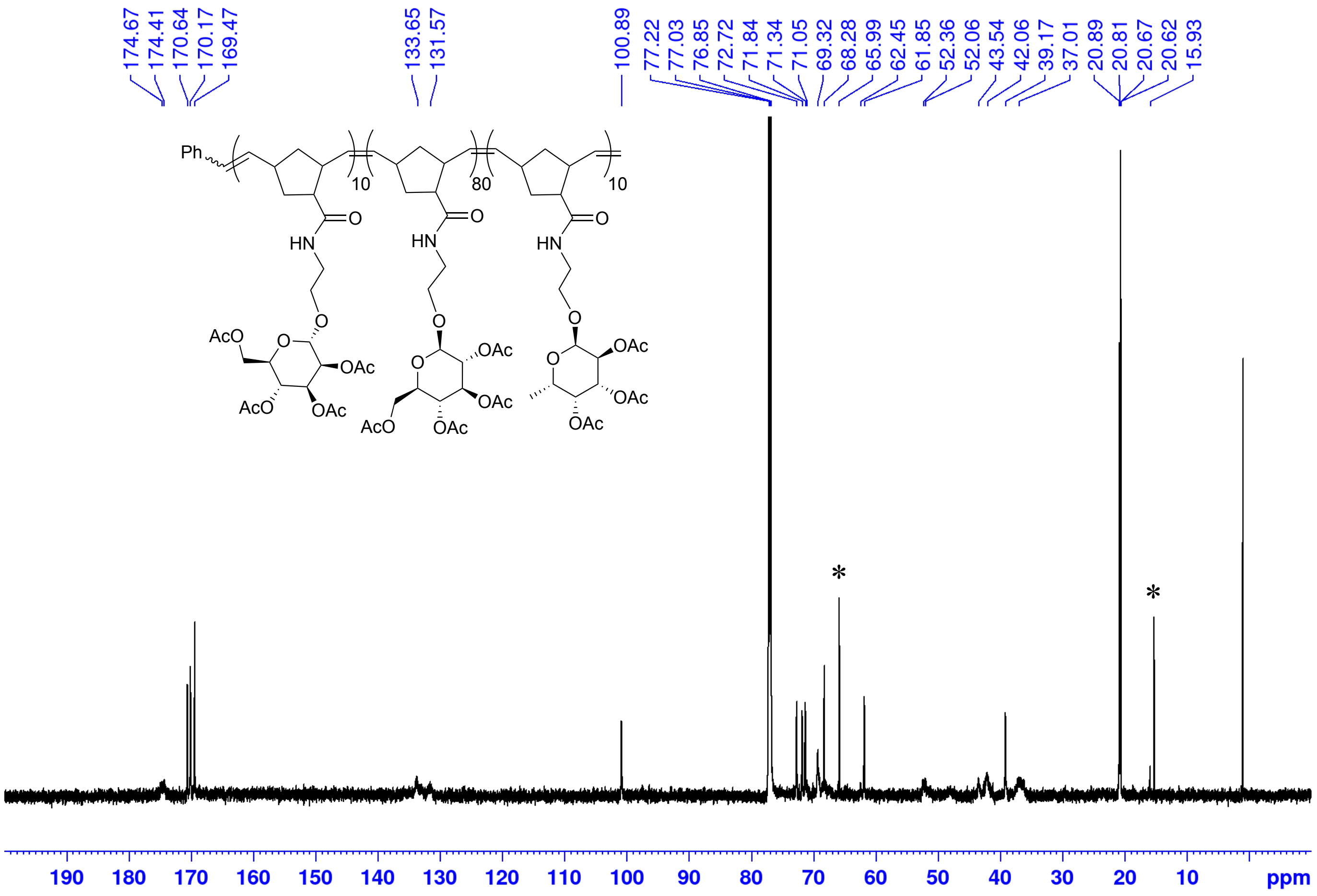


**Figure S21**. $^{13}$C NMR (176 MHz, $CDCl_3$) spectrum of poly(**1a′**)$_{10}$-*block*-poly(**1c′**)$_{80}$-*block*-poly(**1b′**)$_{10}$

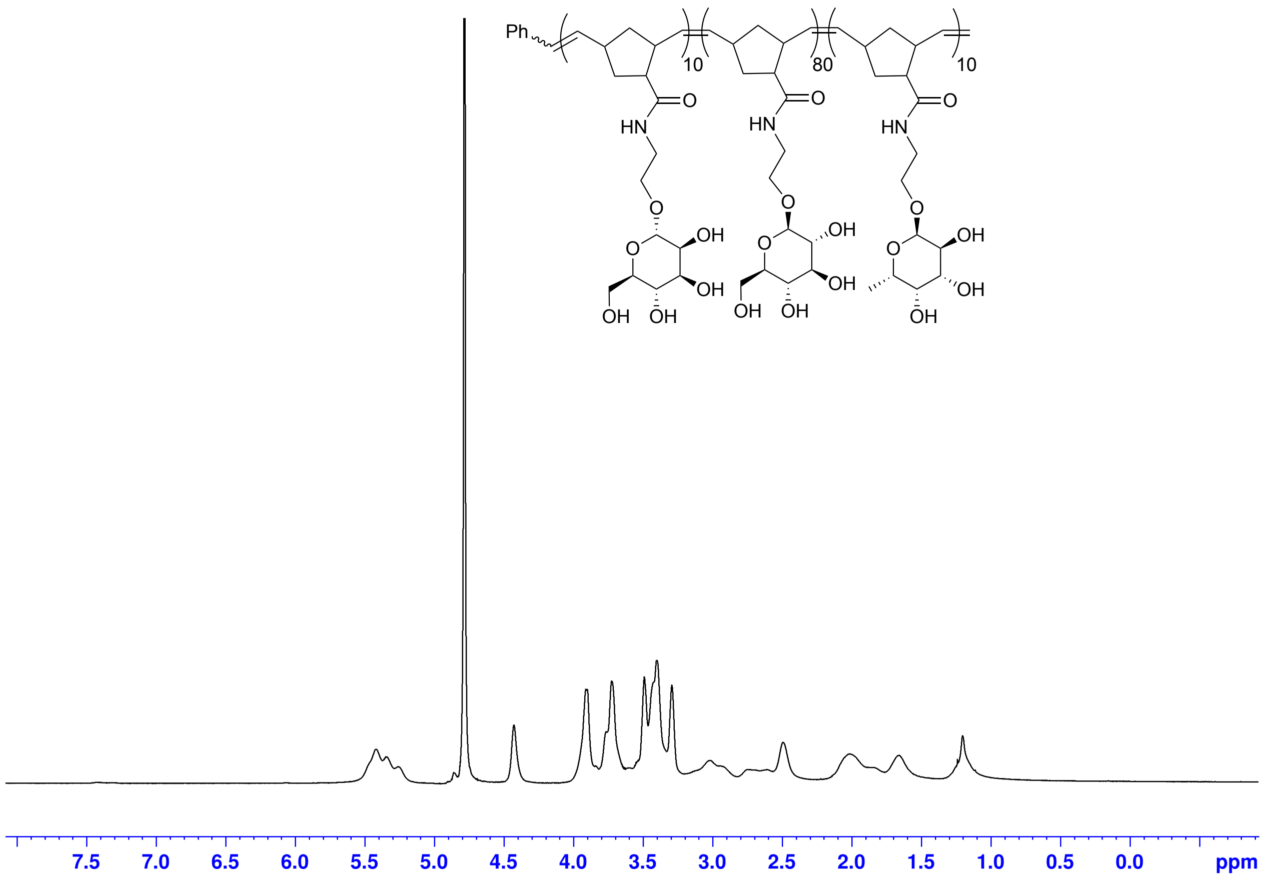


**Figure S22**. $^{1}$H NMR (700 MHz, $D_2O$) spectrum of poly(**1a**)$_{10}$-*block*-poly(**1c**)$_{80}$-*block*-poly(**1b**)$_{10}$

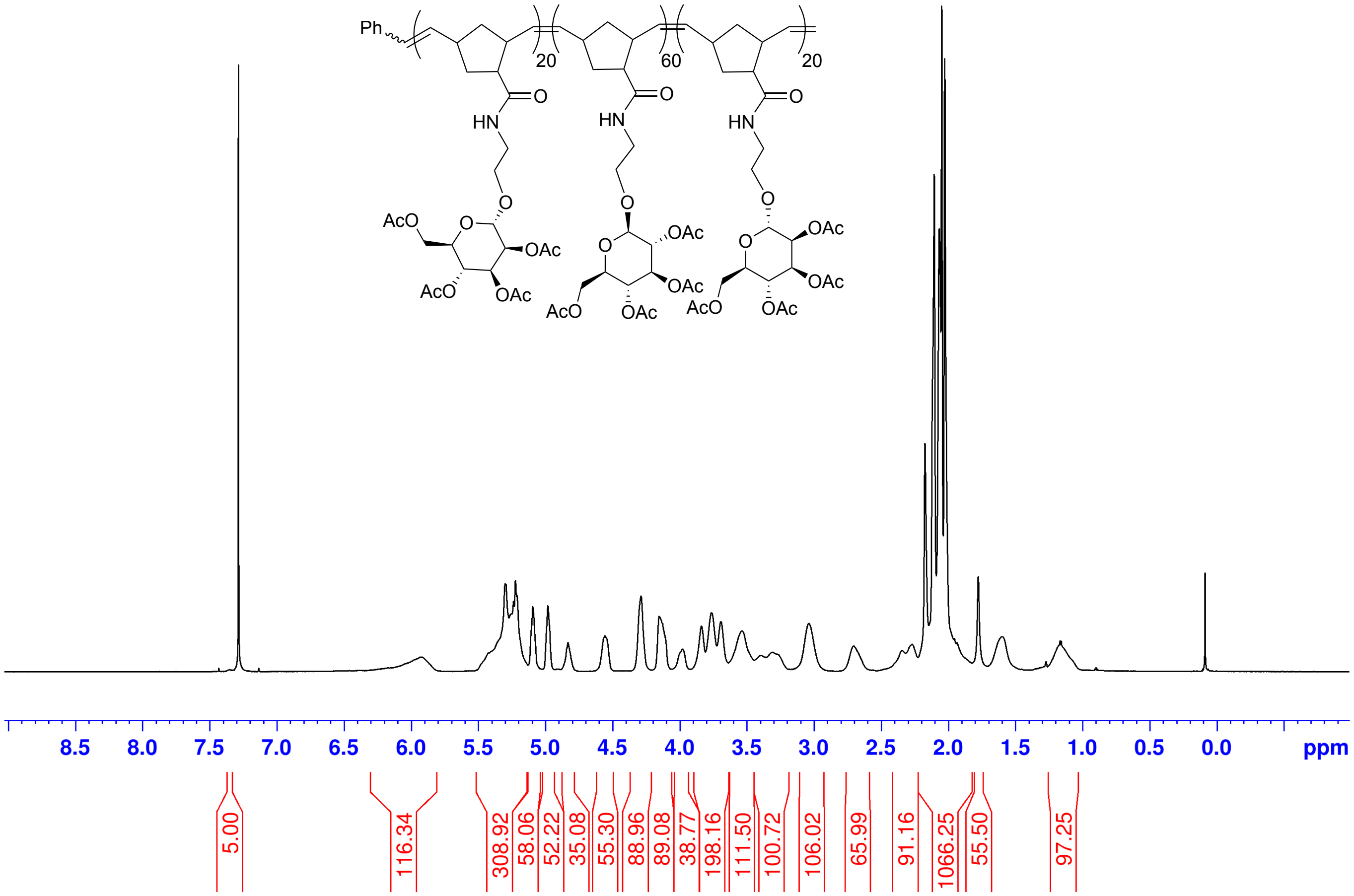


**Figure S23**. $^{1}$H NMR (700 MHz, $CDCl_3$) spectrum of poly(**1a′**)$_{20}$-*block*-poly(**1c′**)$_{60}$-*block*-poly(**1a′**)$_{20}$

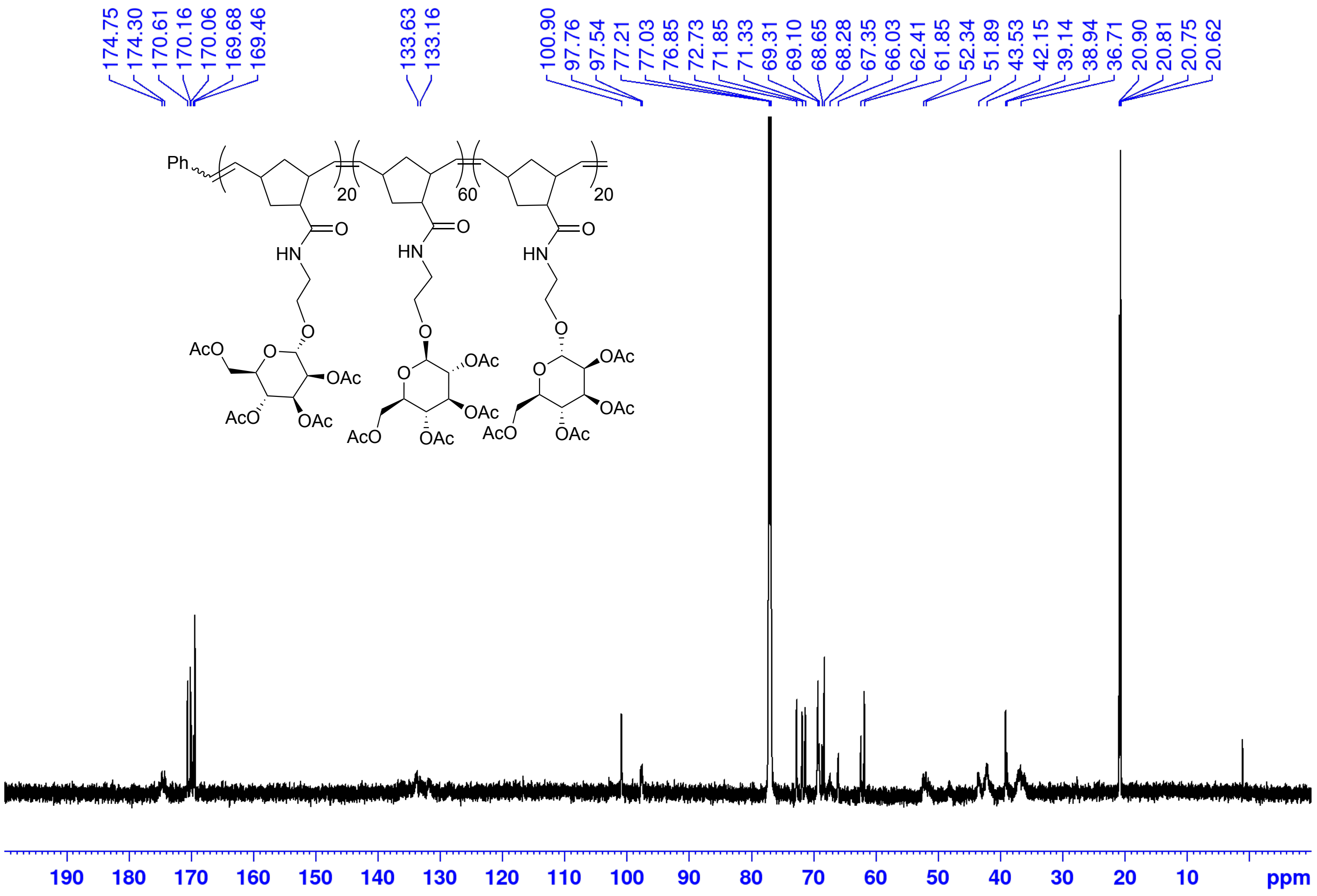


**Figure S24**. $^{13}$C NMR (176 MHz, $CDCl_3$) spectrum of poly(**1a′**)$_{20}$-*block*-poly(**1c′**)$_{60}$-*block*-poly(**1a′**)$_{20}$

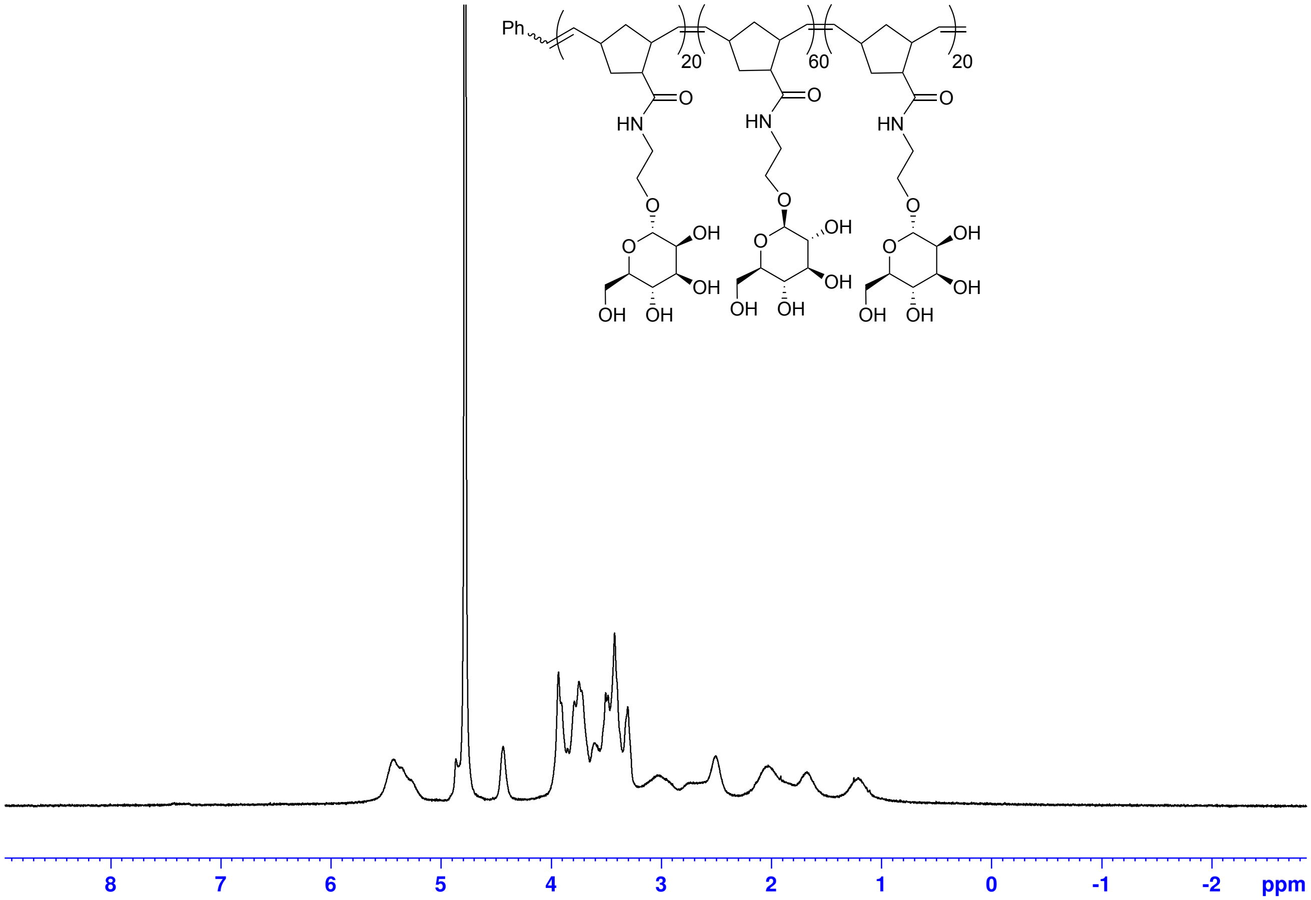


**Figure S25**. $^{1}$H NMR (400 MHz, $D_2O$) spectrum of poly(**1a**)$_{20}$-*block*-poly(**1c**)$_{60}$-*block*-poly(**1a**)$_{20}$

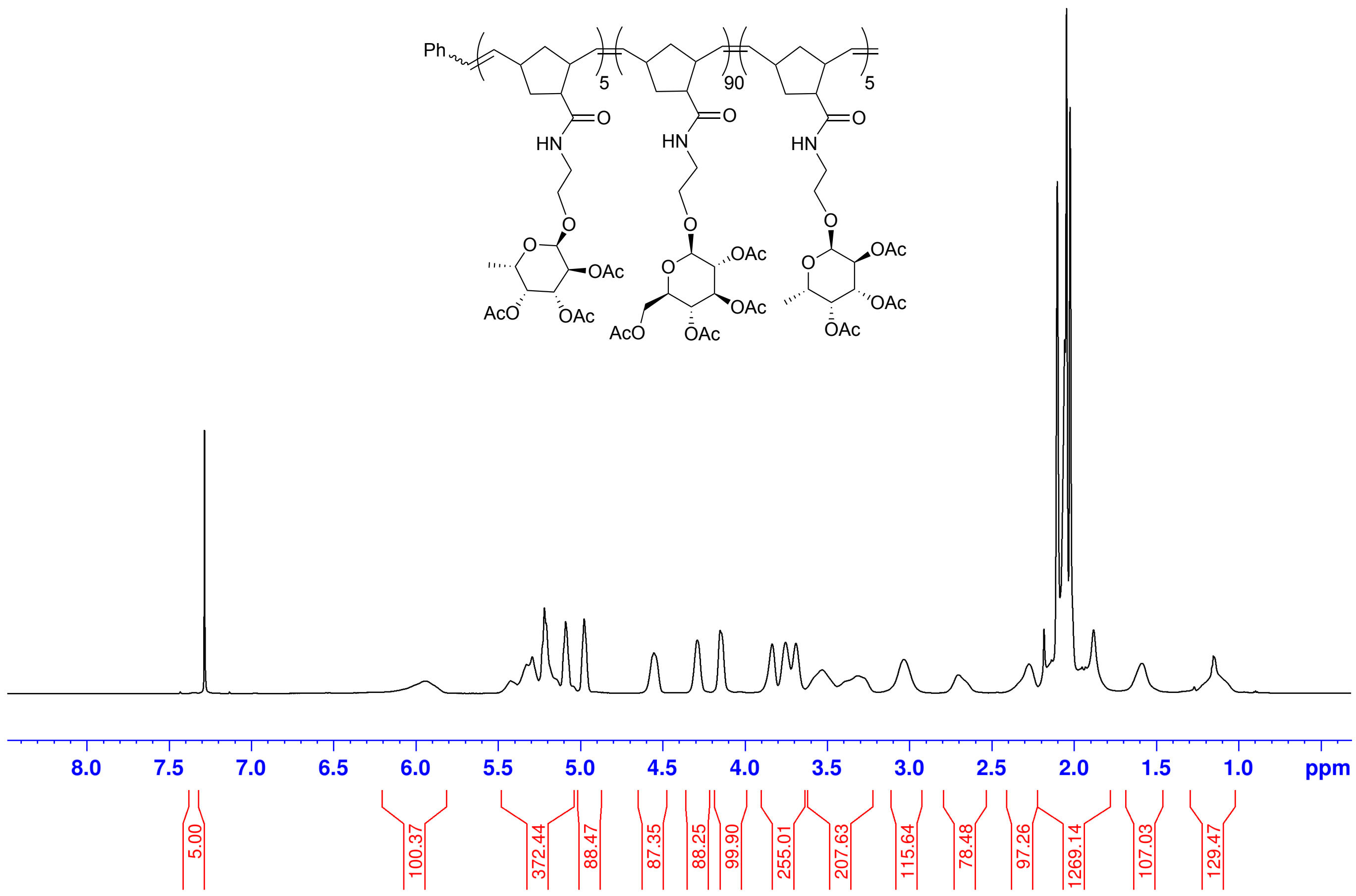


**Figure S26**. $^{1}$H NMR (700 MHz, $CDCl_3$) spectrum of poly(**1b′**)$_5$-*block*-poly(**1c′**)$_{90}$-*block*-poly(**1b′**)$_5$

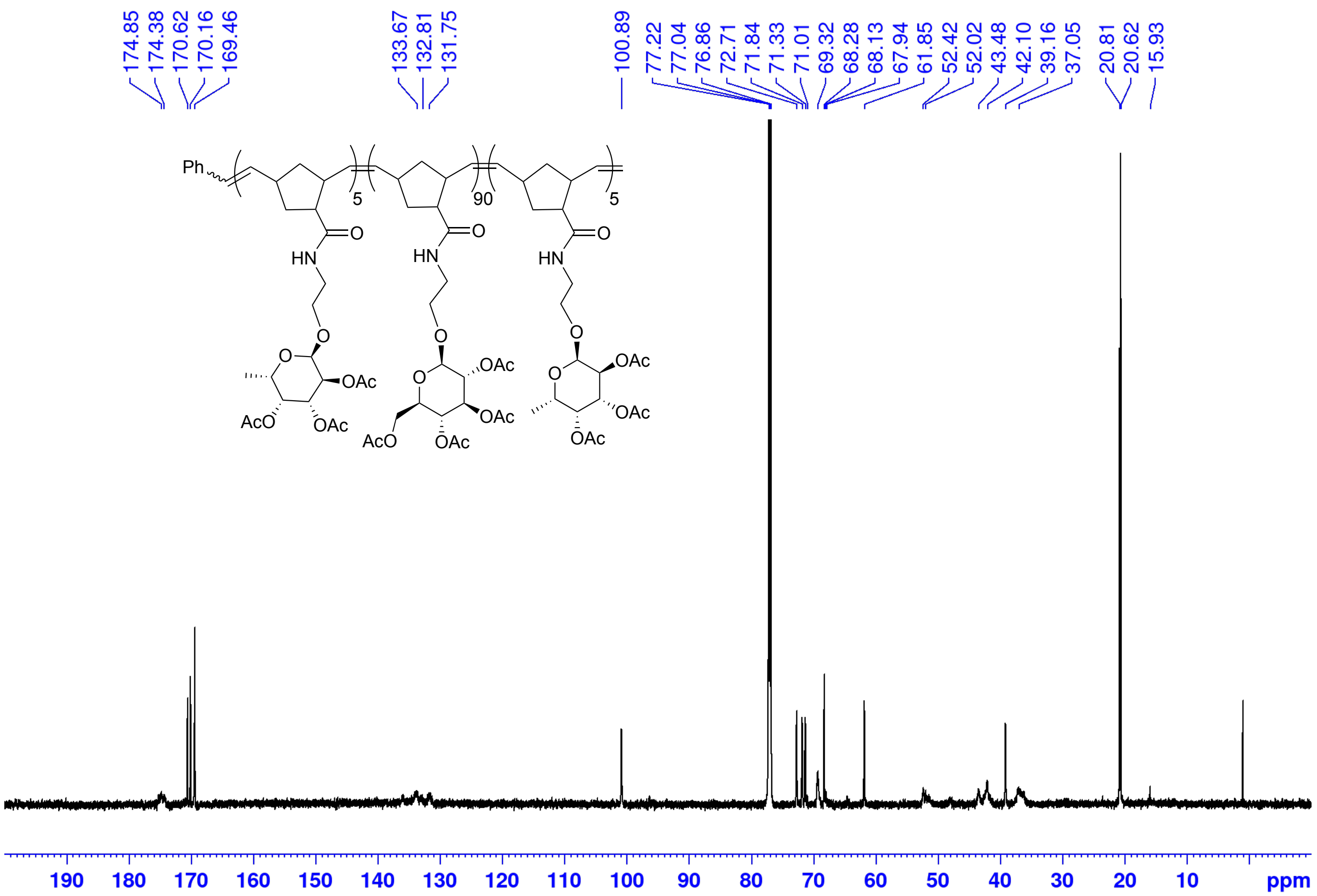


**Figure S27**. $^{13}$C NMR (176 MHz, $CDCl_3$) spectrum of poly(**1b′**)$_5$-*block*-poly(**1c′**)$_{90}$-*block*-poly(**1b′**)$_5$

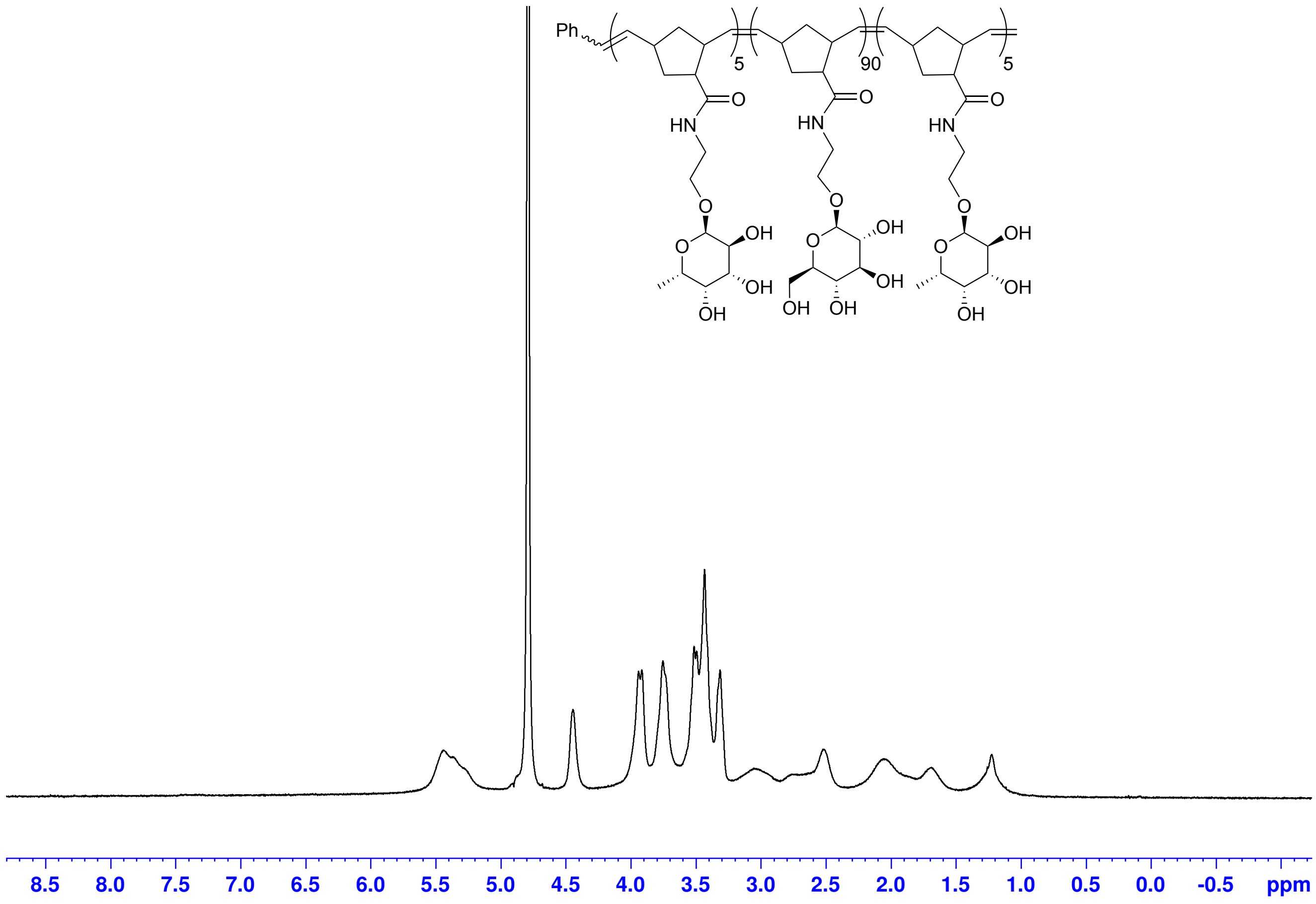


**Figure S28**. $^{1}$H NMR (400 MHz, $D_2O$) spectrum of poly(**1b**)$_5$-*block*-poly(**1c**)$_{90}$-*block*-poly(**1b**)$_5$

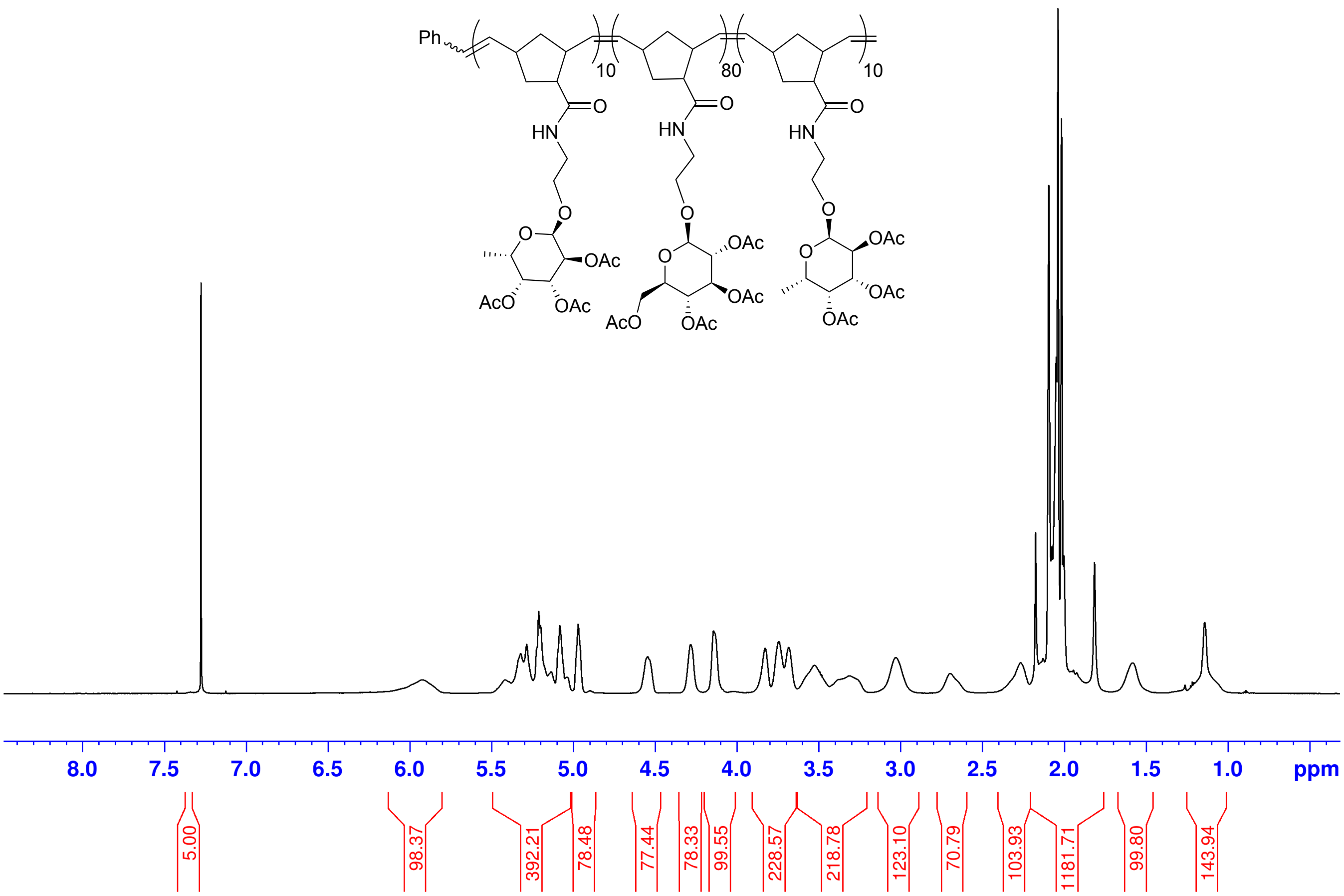


**Figure S29**. $^{1}$H NMR (700 MHz, $CDCl_3$) spectrum of poly(**1b′**)$_{10}$-*block*-poly(**1c′**)$_{80}$-*block*-poly(**1b′**)$_{10}$

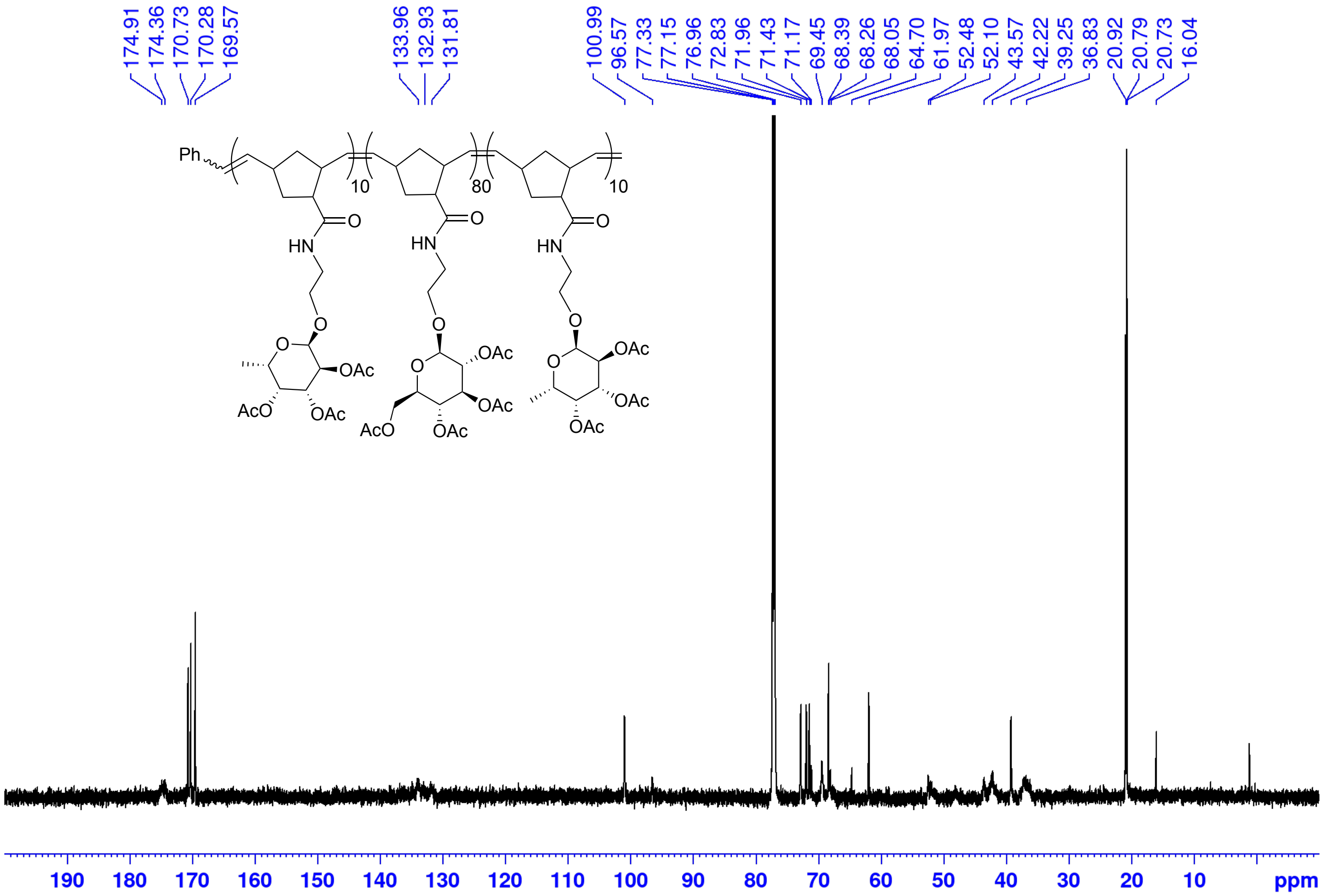


**Figure S30**. $^{13}$C NMR (176 MHz, $CDCl_3$) spectrum of poly(**1b′**)$_{10}$-*block*-poly(**1c′**)$_{80}$-*block*-poly(**1b′**)$_{10}$

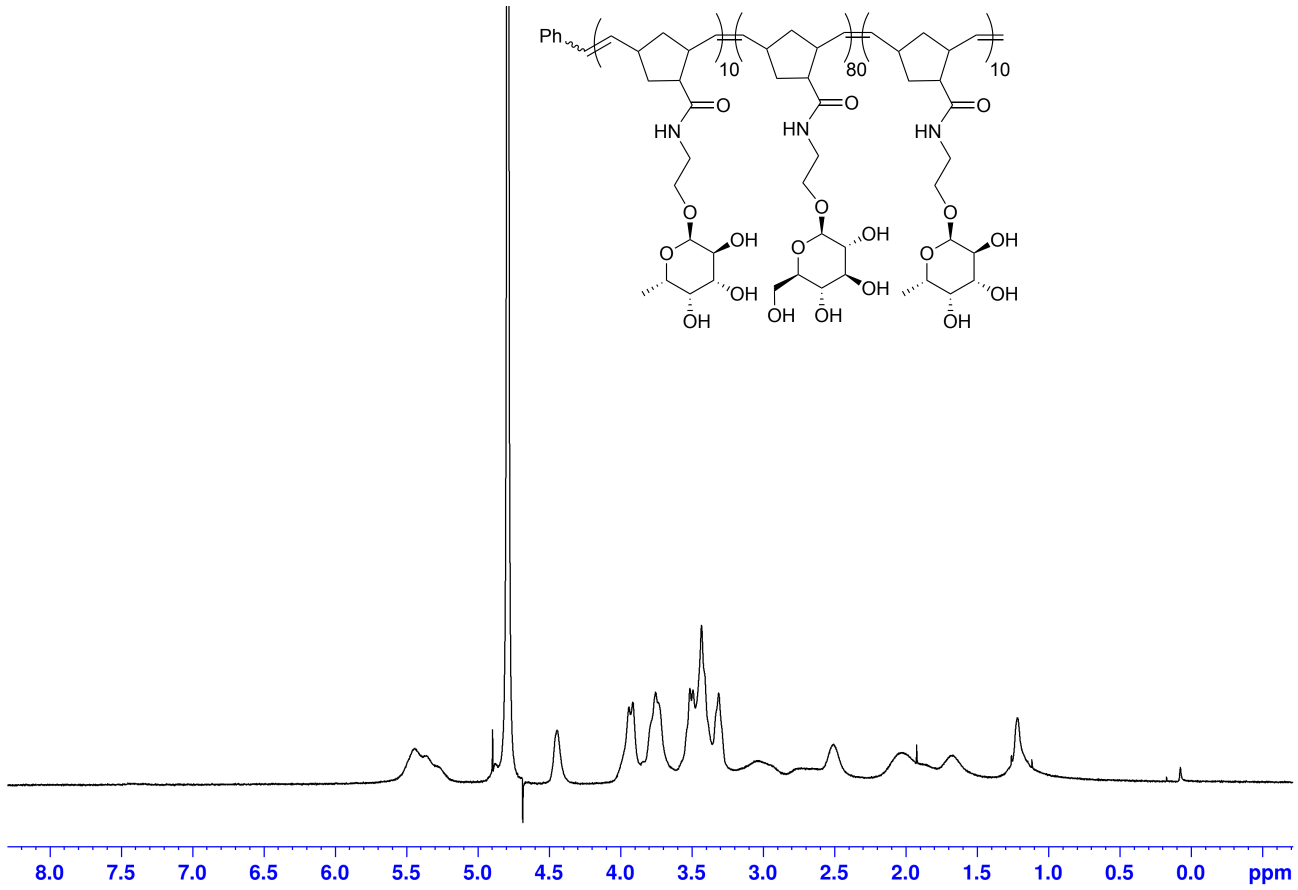


**Figure S31**. $^{1}$H NMR (400 MHz, $D_2O$) spectrum of poly(**1b**)$_{10}$-*block*-poly(**1c**)$_{80}$-*block*-poly(**1b**)$_{10}$

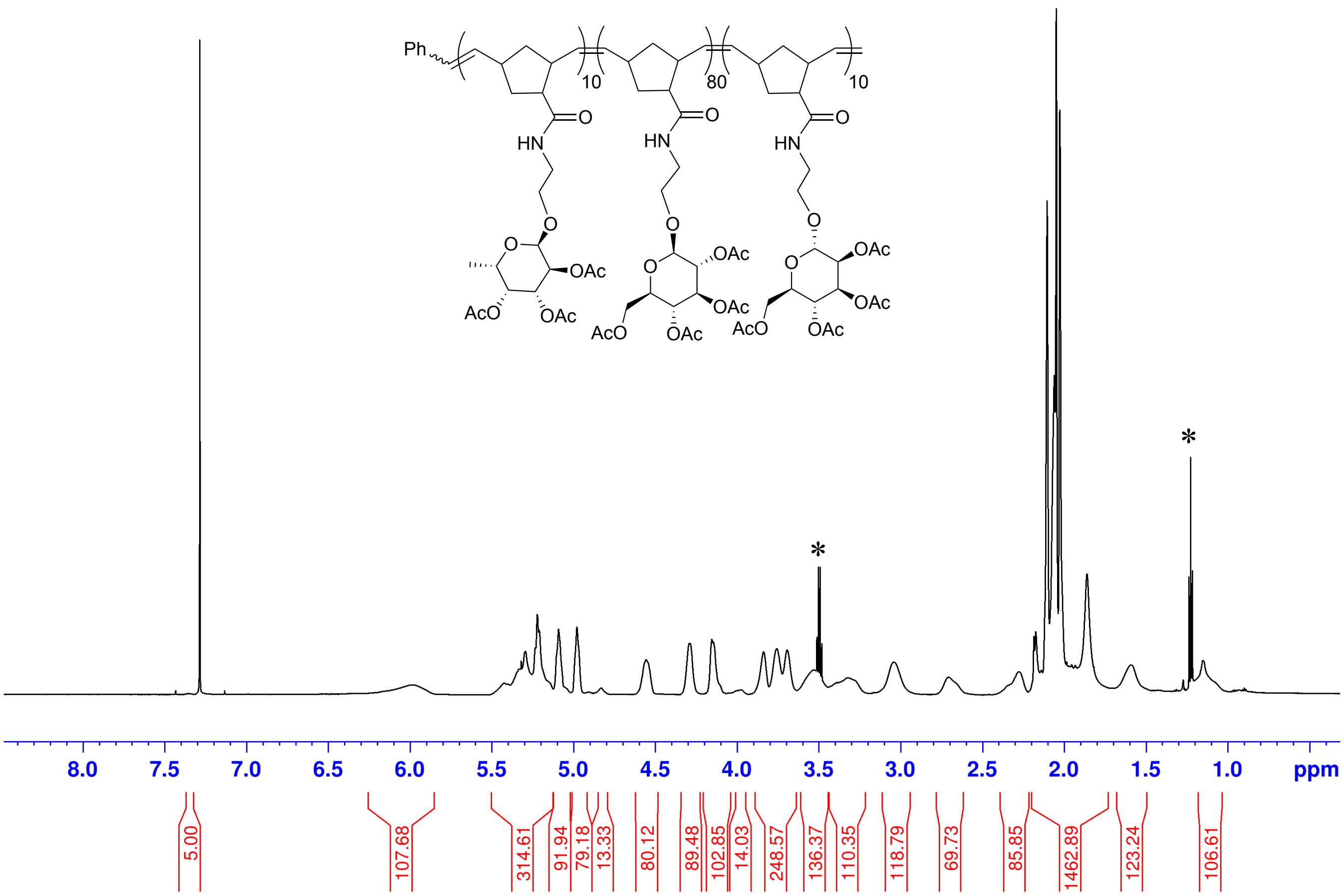


**Figure S32**. $^1H$ NMR (700 MHz, $CDCl_3$) spectrum of poly(**1b′**)$_{10}$-*block*-poly(**1c′**)$_{80}$-*block*-poly(**1a′**)$_{10}$

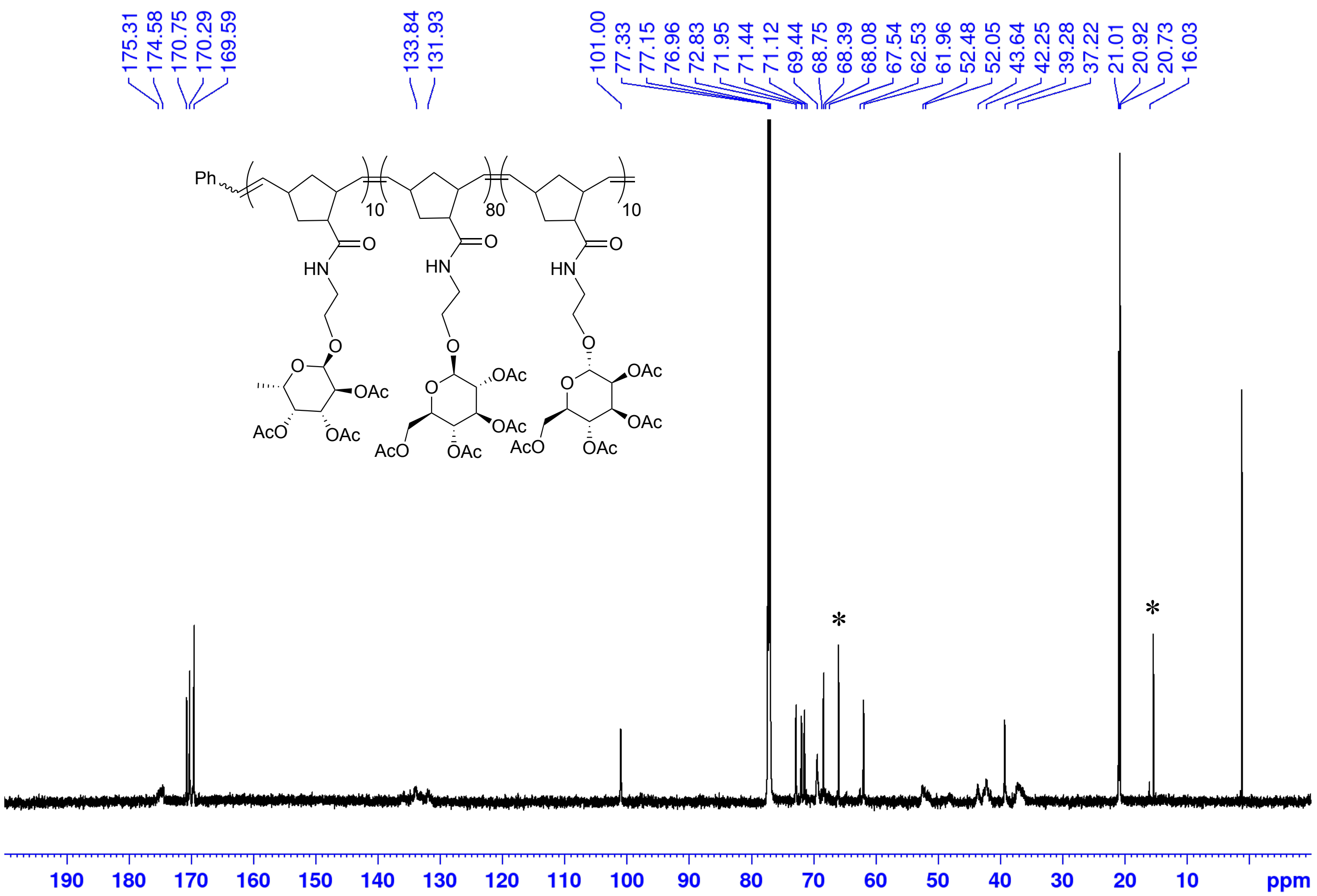


**Figure S33**. $^{13}$C NMR (176 MHz, $CDCl_3$) spectrum of poly(**1b′**)$_{10}$-*block*-poly(**1c′**)$_{80}$-*block*-poly(**1a′**)$_{10}$

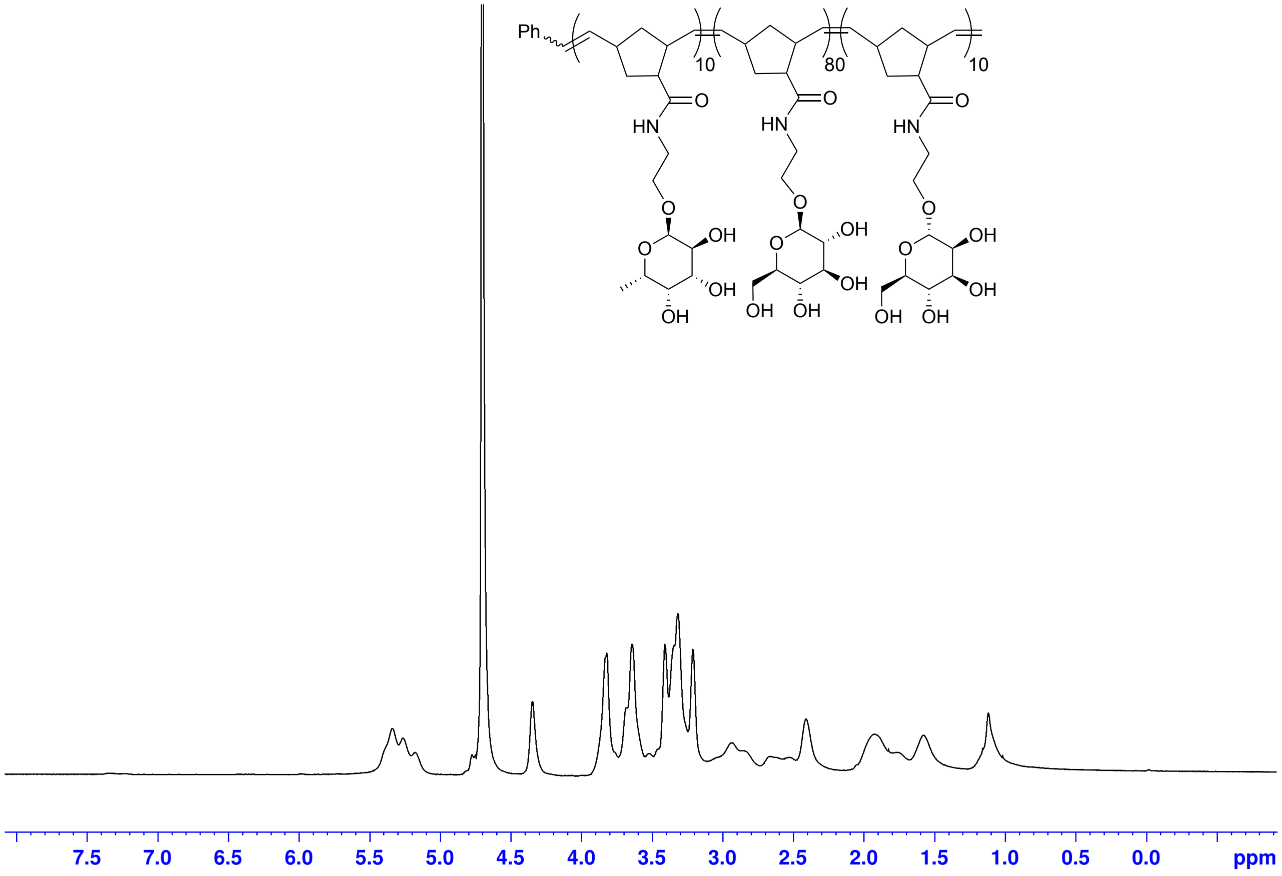


**Figure S34**. $^{1}$H NMR (700 MHz, $D_2O$) spectrum of poly(**1b**)$_{10}$-*block*-poly(**1c**)$_{80}$-*block*-poly(**1a**)$_{10}$

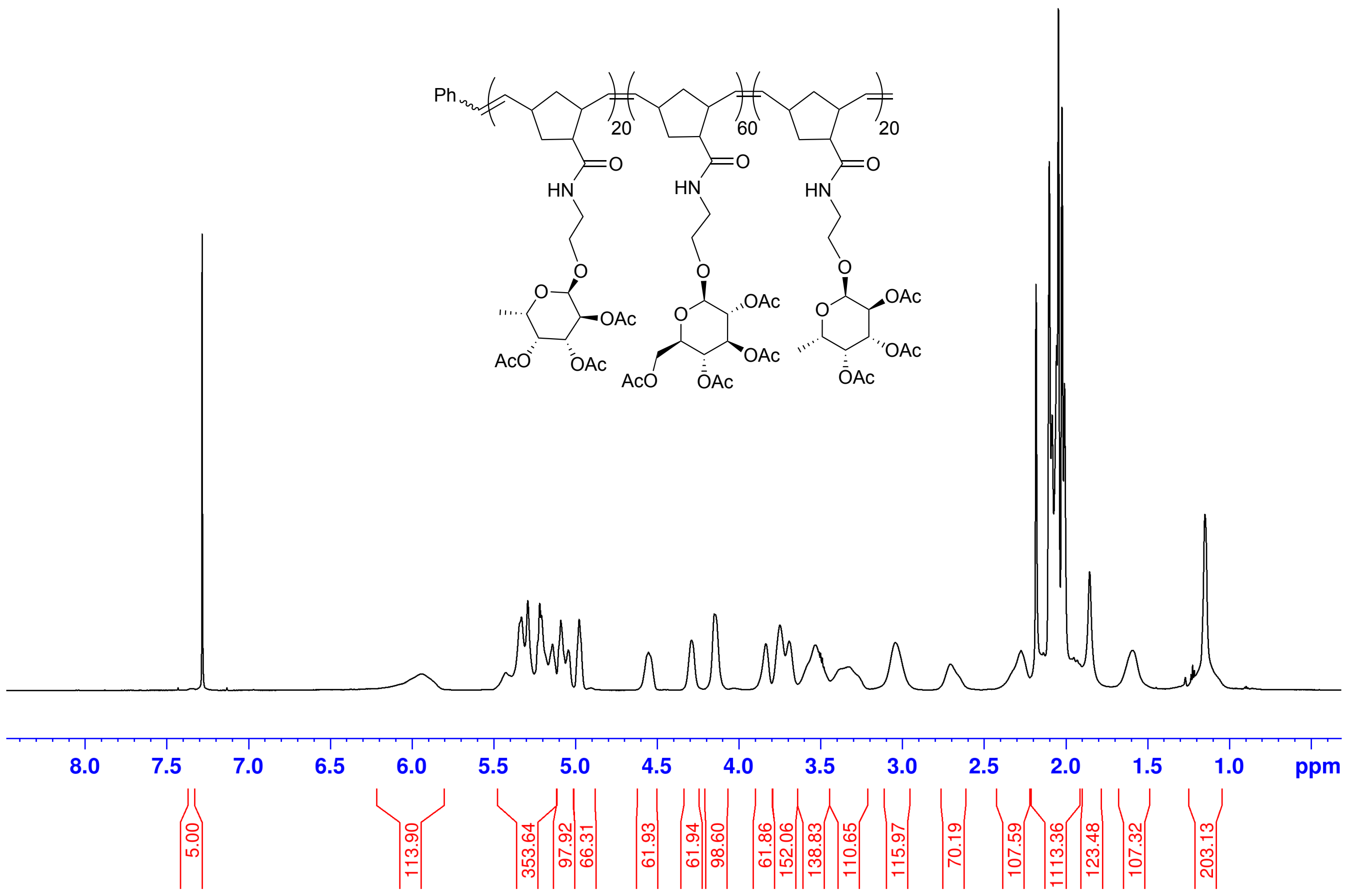


**Figure S35**. $^{1}$H NMR (700 MHz, $CDCl_3$) spectrum of poly(**1b′**)$_{20}$-*block*-poly(**1c′**)$_{60}$-*block*-poly(**1b′**)$_{20}$

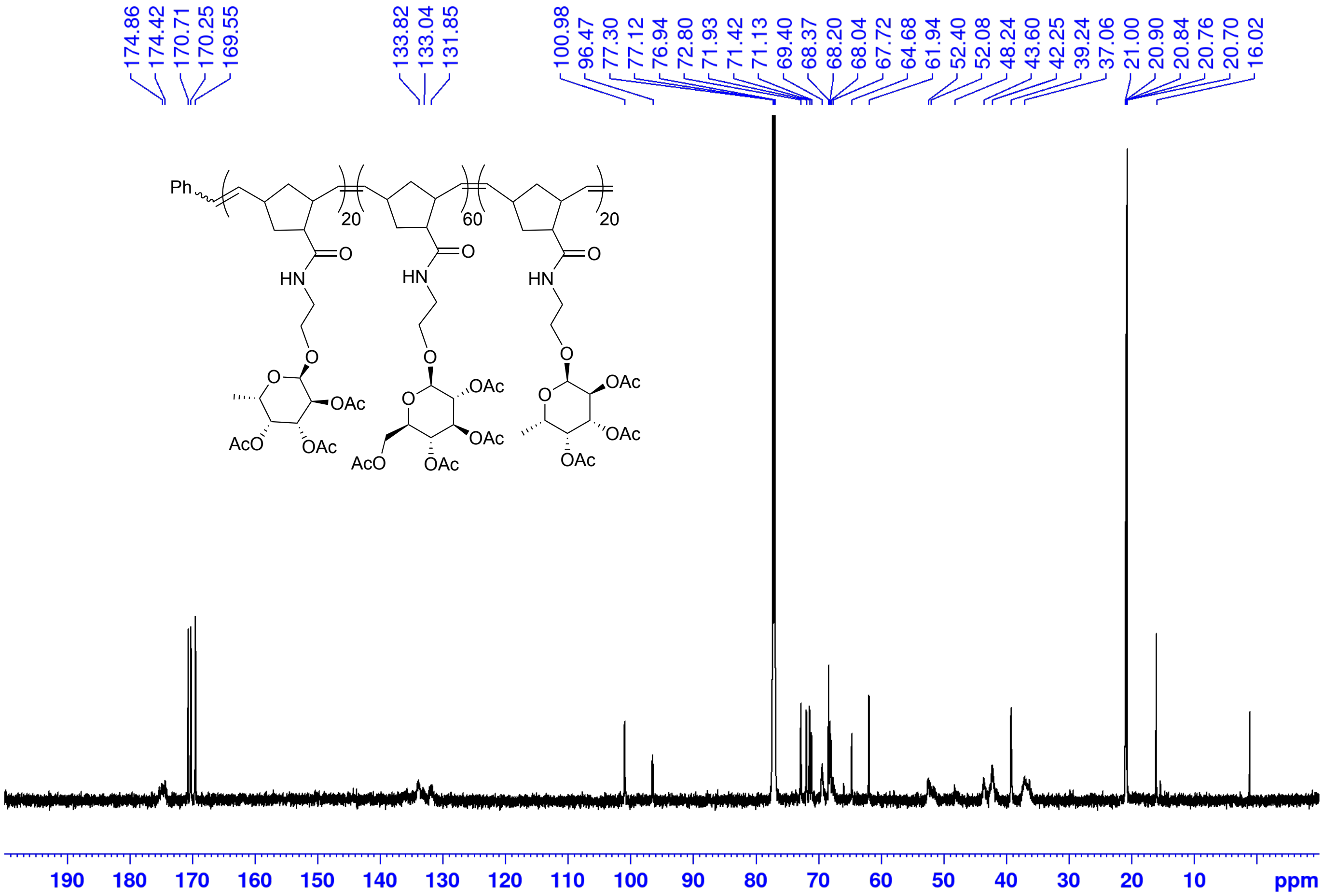


**Figure S36**. $^{13}$C NMR (176 MHz, $CDCl_3$) spectrum of poly(**1b′**)$_{20}$-*block*-poly(**1c′**)$_{60}$-*block*-poly(**1b′**)$_{20}$

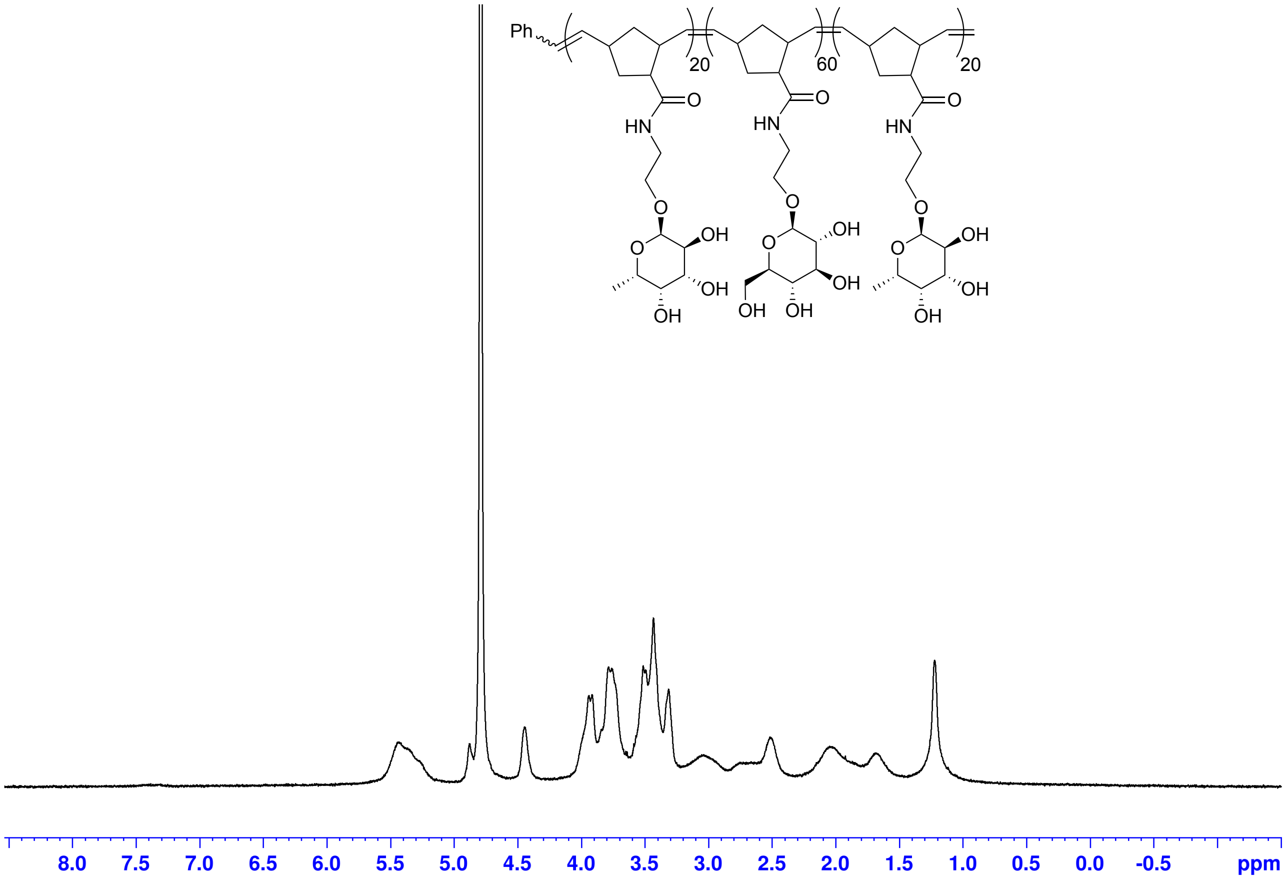


**Figure S37**. $^{1}$H NMR (400 MHz, $D_2O$) spectrum of poly(**1b**)$_{20}$-*block*-poly(**1c**)$_{60}$-*block*-poly(**1b**)$_{20}$
